# Prediction of activity and selectivity profiles of human Carbonic Anhydrase inhibitors using machine learning classification models

**DOI:** 10.1186/s13321-021-00499-y

**Published:** 2021-03-06

**Authors:** Annachiara Tinivella, Luca Pinzi, Giulio Rastelli

**Affiliations:** 1 Department of Life Sciences, University of Modena and Reggio Emilia, Via Giuseppe Campi 103, 41125 Modena, Italy; 2 Clinical and Experimental Medicine PhD Program, University of Modena and Reggio Emilia, Modena, Italy

**Keywords:** Machine learning, Carbonic anhydrase, Selectivity

## Abstract

The development of selective inhibitors of the clinically relevant human Carbonic Anhydrase (hCA) isoforms IX and XII has become a major topic in drug research, due to their deregulation in several types of cancer. Indeed, the selective inhibition of these two isoforms, especially with respect to the homeostatic isoform II, holds great promise to develop anticancer drugs with limited side effects. Therefore, the development of in silico models able to predict the activity and selectivity against the desired isoform(s) is of central interest. In this work, we have developed a series of machine learning classification models, trained on high confidence data extracted from ChEMBL, able to predict the activity and selectivity profiles of ligands for human Carbonic Anhydrase isoforms II, IX and XII. The training datasets were built with a procedure that made use of flexible bioactivity thresholds to obtain well-balanced active and inactive classes. We used multiple algorithms and sampling sizes to finally select activity models able to classify active or inactive molecules with excellent performances. Remarkably, the results herein reported turned out to be better than those obtained by models built with the classic approach of selecting an a priori activity threshold. The sequential application of such validated models enables virtual screening to be performed in a fast and more reliable way to predict the activity and selectivity profiles against the investigated isoforms.

**Supplementary Information:**

The online version contains supplementary material available at 10.1186/s13321-021-00499-y.

## Introduction

Human Carbonic Anhydrases (hCA) represent a family of targets widely studied for their role both in homeostasis and in a number of pathological conditions [1]. In particular, hCA are metalloenzymes, belonging to the class of lyases, which catalyze the reversible hydration of carbon dioxide (CO_2_) to bicarbonate ion (${\text{HCO}}_{3}^{ - }$), with one proton release (H^+^). To date, 15 different isoforms of hCA have been identified, 12 of which display catalytic activity [1]. All catalytic hCAs present a highly conserved inner binding cavity coordinating a zinc ion (Zn^2+^), necessary for the hydration of carbon dioxide [1]. Accordingly, the vast majority of known hCA inhibitors present a zinc binding group (ZBG), which is very often a primary sulfonamide [2]. The first hCA inhibitors bearing a sulfonamide-based ZBG were developed in the 1940s, with acetazolamide being the first drug approved in 1954 [2]. Unfortunately, these molecules tended to have short half-lives and to also be active on other isoforms with a physiological role in homeostasis, resulting in undesirable side effects [2]. In recent years, a considerable interest has arisen for the clinically relevant isoforms IX and XII (hCA IX and hCA XII, respectively), which have been found to be overexpressed in several types of cancers, and especially in hypoxic tumors [3, 4].

The therapeutic relevance of hCA IX and hCA XII as potential drug targets against hypoxic tumors can be explained as follows. In cancer cells with a hypoxic phenotype, the metabolic balance is shifted towards glycolysis under anaerobic conditions, as opposed to oxidative phosphorylation (Warbug effect) [5, 6]. This would normally result in a massive extrusion of lactic acid lowering the extracellular pH, and thus providing unfavorable conditions for cell proliferation. However, tumor cells with a hypoxic phenotype can overexpress hCA IX and/or hCA XII as an adaptive response, which convert carbon dioxide produced inside the cell to bicarbonate ion, to reduce the acidity in the extracellular space. This allows tumor cells to become highly proliferative, invasive, and resistant to several therapies, making them difficult to treat with current clinical approaches [5].

Computational approaches have already been applied to design hCA inhibitors [7–9]. For example, the FDA approved dorzolamid was designed through the application of structure-based (SB) approaches [10]. These approaches, either alone or in combination, enable the modelling of ligands according to their complementary with the binding site of the investigated target [11]. However, although being among the most used screening techniques in the computational field, SB methods are not exempt from limitations. For example, the number of crystallographic structures of the clinically relevant isoforms hCA IX and hCA XII is significantly lower compared to those of hCA II. Likewise, ligand-based (LB) methods such as similarity searching approaches might present some limitations. For example, they might be affected by the adopted similarity measure and the selected reference molecule(s), as well as the algorithm used for the similarity evaluation [12]. Interestingly, the number of small molecules developed and tested against these isoforms and made available in public repositories is steadily increasing. Such a large amount of data makes it possible to use more sophisticated LB techniques. For example, very recently Poli et al. reported a fingerprint-based cheminformatics platform able to successfully cluster known hCA inhibitors from PubChem and to highlight structure-selectivity relationships [13]. On a different note, the use of machine learning approaches has become extremely attractive, as testified by the growing number of studies reported on this topic [14–21], but so far, to the best of our knowledge, these methods have not been applied to investigate hCA activity and isoform selectivity.

In this study, we aimed at training machine learning models able to predict the activity and selectivity profiles of hCA inhibitors, by using a set of molecular descriptors. In particular, models were trained on classifying groups of molecules with high difference in activity between the homeostatic hCA isoform II and the tumor related isoforms hCA IX and hCA XII. We used ten different classification algorithms to build models, which were then tested and validated against previously unseen datasets. We obtained excellent levels of performance according to different, validated metrics. In particular, we found that the use of a probability score as a ranking method led to a decrease in the number of false positives, yielding models that outperformed those built using pre-established activity thresholds, built on larger datasets.

Finally, we selected the best models, which were able to correctly classify active *vs* inactive instances in the training, testing and validations phases. Moreover, from the combination of validated activity labels we could predict and discuss the selectivity profile of specific examples out of the validation dataset. In conclusion, this study provides evidence that the application of sequential binary classification models, combined with the use of probability scores, can be used for virtual screening campaigns able to recognize with high confidence the most likely active and selective molecules against the investigated isoforms.

## Results and discussion

### Activity profiling

In this study, we trained and tested machine learning models based on molecular descriptors to predict activity and selectivity profiles of a set of reported human Carbonic Anhydrases (hCAs) inhibitors. To this aim, we first generated a curated dataset of bioactivities on the human Carbonic Anhydrase targets. In particular, compounds with activity reported for hCA II, IX and XII were downloaded from the ChEMBL database (release 26, accessed on March 20th, 2020) [22]. To ensure that the dataset contained curated and comparable data, we took into account only annotations that derived from tests on single proteins and activities expressed as K_i_ and IC_50_. This procedure enabled the collection of 6,396 unique inhibitors with 18,857 activity records (the dataset downloaded from ChEMBL is given as Additional file 1).

**Fig. 1:**
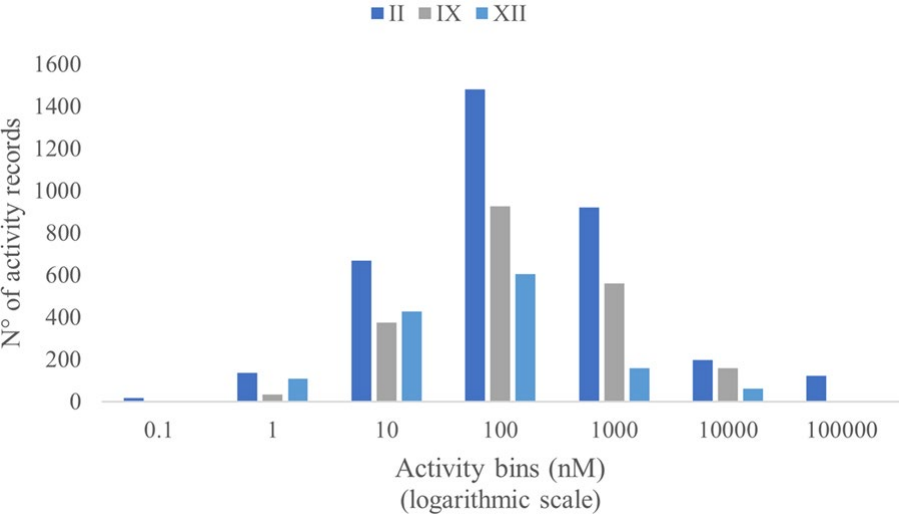
Activity distributions for the hCA II, hCA IX and hCA XII ChEMBL inhibitors with a primary sulfonamide ZBG, after merging and filtration of the outliers

Additional filtering was performed on the initial dataset to retain only molecules with a primary sulfonamide zinc binding group (ZBG), which are expected to modulate hCAs through the same mechanism of action. This operation allowed us to exclude allosteric inhibitors (often binding to the outermost part of the binding pocket) and compounds bearing uncommon ZBGs, which are likely to be less validated. Indeed, the vast majority of hCA inhibitors reported in the literature present a ZBG based on a primary sulfonamide [2]. Preliminary analyses showed that around 10% of the compounds in the initial dataset have multiple activity records for the same target(s), occasionally with different outcomes. To remove data that would affect the prediction performances of the training models, we first processed molecules with multiple activity records on the same target. In particular, molecules whose standard deviation was lower than 20% of the original mean value were retained. The activity of compounds with more than 5 activity records on the same target and a standard deviation higher than 20% was reported in the dataset as the mode of the observed ChEMBL values (see “Methods” section). This procedure allowed us to collect an appropriate number of compounds for the development of the machine learning models. The KNIME workflow used to filter and prepare ChEMBL data and the resulting processed dataset are given as Additional file 2 and Additional file 3, respectively. The total number of molecules for each isoform and their activity distributions are reported in Table 1 and Fig. 1, respectively.

**Table 1:** Number of bioactivities per hCA isoform in the processed dataset

ChEMBL Target ID	Target name	Target organism	Molecules in the initial dataset
CHEMBL205	Carbonic anhydrase II	Homo sapiens	4166
CHEMBL3594	Carbonic anhydrase IX	Homo sapiens	2310
CHEMBL3242	Carbonic anhydrase XII	Homo sapiens	1654

As shown in Fig. 1, the number of bioactivities for the three isoforms and their distribution are rather uneven. In addition, many of the reported activities are shifted to values below 100 nM for hCA II, while the number of activity data with higher values (“inactive” compounds) is considerably low. This issue is even more pronounced for isoforms IX and XII, most likely because of the tendency not to publish negative results.

As reported in previous studies on other targets [23–26], the first step to develop accurate machine learning models for binary classification should be the definition of an activity threshold to split active and inactive classes, or alternatively two thresholds to further separate a class of intermediate activities. In the absence of specific activity thresholds reported in previous studies on hCA isoforms, we initially classified molecules as active or inactive by observing the activities distribution for the three isoforms. We established two thresholds, i.e. a molecule was classified as active when its activity is below 20 nM, and inactive when the activity is above 100 nM. As reported in Table 2, the resulting classes appeared to be highly unbalanced, both within the same isoform and across the different isoforms. Additional explored activity thresholds are reported in Additional file 4: Table S1. It has been previously reported that training on such unbalanced datasets would negatively affect the ability of the machine learning models in predicting both classes (“active” *vs* “inactive”) with equal performance [27].

**Table 2:** Number of active and inactive compounds for each isoform, according to fixed activity thresholds

Activity values	hCA II (count)	hCA IX (count)	hCA XII (count)
< 20 nM (active)	1853	880	942
20–100 nM (intermediate)	1068	697	466
≥ nM (inactive)	1245	733	246

We considered active compounds those with reported activity in the processed dataset below 20 nM, while those with activity above 100 nM were considered as inactives

Therefore, we applied a sampling procedure, unrelated to the choice of fixed bioactivity thresholds, to address this issue. In particular, the activities obtained through the merge and filtration process were first ranked in ascending order. Then, we performed sampling of groups formed by equal size of the first* N* molecules (active class) and the last* N* molecules (inactive class) for each of the three isoforms. Table 3 shows how maximum and minimum values for each class vary with the sampling size* N* values.

**Table 3:** Number of instances in "active" and "inactive" classes by sampling groups of equal size (group size = *N*)

*N*	hCA II			hCA IX			hCA XII		
	Max Active (nM)	Min Inactive (nM)	Closest ratio	Max Active (nM)	Min Inactive (nM)	Closest ratio	Max Active (nM)	Min Inactive (nM)	Closest ratio
150	0.83	10,000	*12,048.19*	2.8	1213	*433.21*	1.8	397	*220.56*
200	1	4550	*4550.00*	3.429	710	*207.06*	2.7	200	*74.07*
250	1.4	2675	*1910.71*	4.5	464	*103.11*	3.4	96.7	*28.44*
300	1.7	1290	*758.82*	5.2	360	*69.23*	3.94	82.3	*20.89*
350	2	860	*430.00*	6	282	*47.00*	4.7	73.5	*15.64*
400	2.1	711	*338.57*	6.6	248	*37.58*	5.4	62.1	*11.50*
450	2.6	626	*240.77*	7.3	220	*30.14*	5.9	53.1	*9.00*
500	3	550	*183.33*	7.9	190.5	*24.11*	6.4	45.4	*7.09*
550	3.3	484	*146.67*	8.5	162	*19.06*	7	39.1	*5.59*
600	3.845	431	*112.09*	9.1	137.1	*15.07*	7.5	33.25	*4.43*
650	4.2	390	*92.86*	9.8	121	*12.35*	8.1	27.8	*3.43*
700	4.7	354	*75.32*	11.7	106	*9.06*	8.6	21	*2.44*
750	5	314	*62.80*	13.5	95	*7.04*	9.2	15.6	*1.70*
800	5.2	280	*53.85*	16	86.4	*5.40*	10	12	*1.20*
850	5.6	258	*46.07*	18	78.1	*4.34*	–	–	–
900	6	235	*39.17*	21	70.3	*3.35*	–	–	–
950	6.3	210	*33.33*	23.2	63.5	*2.74*	–	–	–
1000	6.8	180.2	*26.50*	25.4	54	*2.13*	–	–	–
1100	7.5	133	*17.73*	31.6	41	*1.30*	–	–	–
1200	8.1	106	*13.09*	–	–	–	–	–	–
1300	9	92	*10.22*	–	–	–	–	–	–
1400	10	80	*8.00*	–	–	–	–	–	–
1500	11	70	*6.36*	–	–	–	–	–	–
1600	13	60	*4.62*	–	–	–	–	–	–

The ratio between the last active and first inactive compounds is reported in italics in the “Closest ratio” column

For instance, a marked difference of activity between active and the inactive classes at all sampling sizes was noted for isoform II. Although less evident, the difference is also present in the sampled groups for isoform IX. On the contrary, when *N* sampling sizes higher than 250 were used for isoform XII, the flexible threshold to define a compound as inactive became less than 100 nM, owing to the limited number of compounds with high reported K_i_ or IC_50_ values on ChEMBL.

**Fig. 2:**
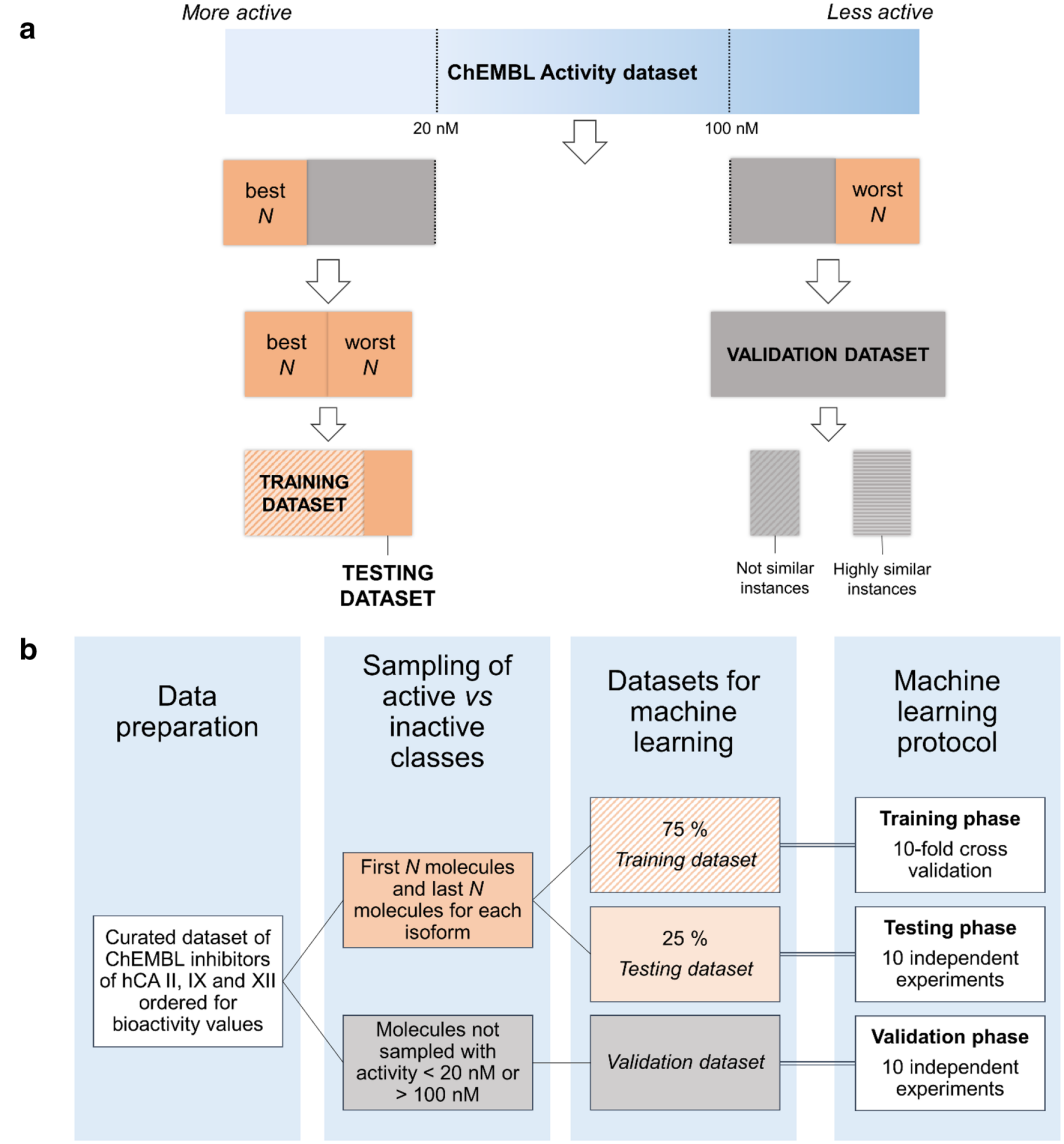
**a** Partitioning of initial data into training, testing and validation datasets. For each isoform, the best *N* molecules were sampled in the “active” class, and the worst *N* in the “inactive” class, according to flexible bioactivity thresholds. Molecules not sampled in the training or testing dataset, and with bioactivities lower than 20 nM or higher than 100 nM were used as a validation dataset. **b** Workflow for machine learning experiments: 75% of the initial dataset was subjected to a tenfold cross validation (training phase) and used to train models against a previously unseen 25% (testing phase). Finally, the whole initial dataset was used to train models to predict the validation dataset (validation phase)

We then built machine learning models using Python *scikit-learn* modules [28]. RDKit molecular descriptors were used to describe each molecule of the initial dataset [29]. The baseline dataset included 118 descriptors (features). Two additional datasets were created by filtering the most correlated features, resulting in the *PCC* = *0.95* dataset (92 descriptors) and *PCC* = *0.75* dataset (57 descriptors) (see Methods). For each isoform, an automated procedure was developed to create a dataset containing the first* N* and the last* N* molecules according to the reported bioactivity data. We set apart 25% of the initial dataset to be used in the testing phase, preserving the distribution of the two classes (see Fig. 2). The remaining 75% was subjected to a cross-validation procedure for each of the selected 10 classification algorithms. The prediction performances were estimated as averages of *accuracy* and *Matthews Correlation Coefficient* (MCC), and the standard deviation related to obtained results was also determined. Moreover, we also considered *precision* and *recall* scores as additional indexes to further evaluate the predictive performances of the models. We investigated *N* sampling sizes ranging from 150 to 700, with increments of 50 units for all isoforms.

The overall results of the training phase are reported in Additional file 4: Tables S2 and S3, in terms of averaged *accuracy* and *MCC* values, respectively. Then, the ability of the models to correctly predict the previously unseen data was assessed (testing phase). In this phase, models were trained on the 75% of the initial dataset, and predictions were made on the remaining 25%, which was set apart at the beginning. The complete results of the testing phase are reported in the Additional file 4: Tables S4 and S5. Models built with the application of a Pearson Correlation Coefficient threshold of 0.95 (herein referred as “*PCC* = *0.95”*) to filter the most correlated features yielded slightly, but consistently better results with respect to the baseline and *PCC* = *0.75* models (see Additional file 4: Tables S2–S5). This shows that several features in the initial dataset were redundant and only provided noise to the training models, while others were significant and informative. Similar results were also obtained when considering *precision* and *recall* score indexes (Additional file 4: Figure S1).

**Fig. 3:**
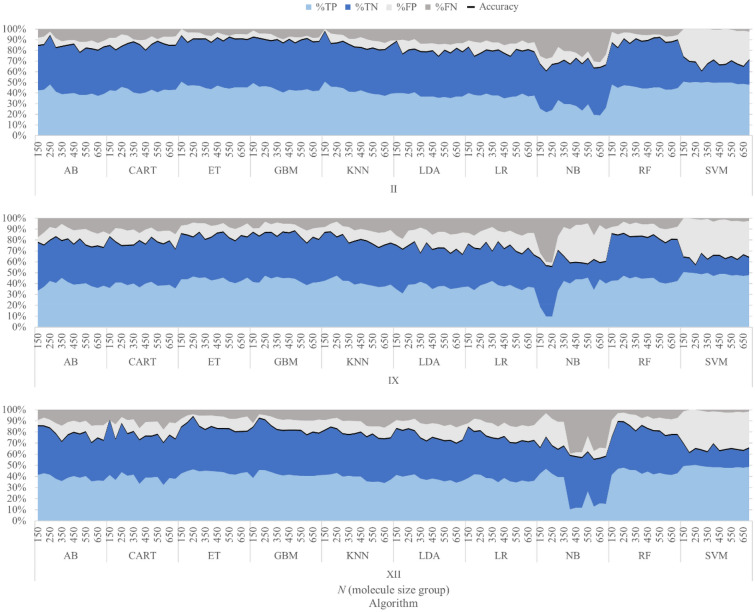
Building models to predict activity classes: representation of variations of TP, TN, FP, and FN percentages as group size (*N*), the algorithm used, and the investigated isoform vary for trained models in the testing phase. All models were built applying a Pearson Correlation Coefficient threshold of 0.95 to filter the most correlated features. The black line represents overall accuracy

A graphical visualization of the results obtained in the testing phase on the *PCC* = *0.95* dataset is shown for each hCA isoform in Fig. 3. In each plot of Fig. 3, the True Positives (correctly predicted actives, %TP), True Negatives (correctly predicted inactives, %TN), False Positives (inactives mislabeled as actives, %FP) and False Negatives (actives mislabeled as inactives, %FN) rates are reported in terms of percentages variation, *per* group size (*N*) and algorithm employed. Moreover, Fig. 3 also reports the variation in the *accuracy* score across the different models (the black line in the plot) and group sizes, as resulting from the sum of the %TP and %TN.

As shown in Fig. 3 and Additional file 4: Table S4, the accuracy values obtained in the testing phase were on average higher than 70% for most methods, with the exception of Support Vector Machines (SVM) and Naïve Bayes (NB) algorithms. Tree-based algorithms, and especially ensemble methods (Random Forest, Extra Tree, Gradient Boosting), proved to be the most efficient, with accuracy scores ranging from 0.77 to 0.97.

For isoforms II and IX the ability to correctly discriminate active from inactive compounds was only marginally affected by the increasing size of the sampling classes. On the contrary, isoform XII experienced a progressive decrease in the performance for all algorithms as the sampling size increased. This was consistent with the fact that an extensive sampling on the dataset curated for this isoform, provided “active” and “inactive” classes with low activity differences (see Table 3).

Similar trends were observed in the prediction performances during the training phase of the models (Additional file 4). Interestingly, in some cases the models built in the testing phase provided slightly better results than those of the training phase. The chemical similarity between the molecules in the training and testing datasets, evaluated by means of the RDKit Atom Pair fingerprints [30], provided Tanimoto coefficients (Tc) below 0.336 (a commonly accepted similarity threshold according to the RDKit documentation [31]). Thus, the higher performances in correctly classifying molecules of the test dataset appear to be more dependent on the quantity of data the models can learn from, rather than to a higher chemical similarity between the molecules in the datasets. Notably, *MCC* values (Additional file 4: Table S5) calculated for both training and testing phases confirmed the previously observed trends, and allowed us to identify Random Forest (RF), and its more randomized variant Extra Tree (ET), as the best algorithms.

Afterwards, we compared the obtained results with those resulting from the use of pre-established activity thresholds (herein referred as “traditional method”) [26]. To this aim, we repeated the same training–testing procedure on 10 algorithms, this time by labeling as “active” the molecules with activity below 20 nM, and as “inactive” molecules with activity above 100 nM (Table 2). These analyses showed that the Extra Tree algorithm was able to provide satisfactory performances also in the “traditional method”, as reported in Additional file 4: Tables S6–S7, but the *accuracy* and *MCC* metrics evaluated for the three best models were considerably lower with respect to those obtained with the flexible bioactivity thresholds proposed here (Table 4). Additional calculations using the fixed threshold method for the Extra Tree algorithm can be found in Additional file 4: Table S8, where molecules were labeled as “active” or “inactive”, according to different activity thresholds.

**Table 4:** Comparison of testing phase results between different sampling approaches

Isoform	Flexible threshold method		Fixed threshold method	
	Accuracy (Max)	MCC (Max)	Accuracy (Max)	MCC (Max)
hCA II	0.86 ± 0.08	0.74 ± 0.15	0.73 ± 0.10	0.45 ± 0.17
hCA IX	0.80 ± 0.10	0.62 ± 0.18	0.73 ± 0.07	0.41 ± 0.18
hCA XII	0.81 ± 0.08	0.92 ± 0.03	0.82 ± 0.15	0.47 ± 0.16

The “flexible threshold method” refers to the sampling method proposed in this work. The “fixed threshold method” refers to the traditional method of sampling the active vs inactive class by choosing fixed bioactivity thresholds (in this case, activity < 20 nM (active class) or ≥ 100 nM (inactive class)

A further validation experiment (herein referred to as “external validation”) was also performed on a set of molecules, not present in the initial training dataset, having reported activity values below 20 nM, or above 100 nM (see Fig. 2). This allowed us to evaluate how the developed models would perform under screening-like conditions. In particular, we first trained the models on the initial dataset, sampled with the first *N* and the last *N* molecules. Then, models were asked to classify the validation dataset by using the Extra Tree algorithm, which performed best in the previous tasks. In this phase, we used a probability score to assess the confidence level of the label predictions (“active” or “inactive”), as implemented in *scikit-learn* (see Methods). In our analyses, probability scores close to 1.0 corresponded to highly confident model predictions, while values closer to 0.5 were considered less reliable. Moreover, as done in the previous analyses, we repeated the predictions for 10 independent experiments and then we averaged the predicted labels and probability scores to further strengthen the results (see Methods). Interestingly, results of these analyses provided low standard deviations (see Additional file 4: Table S9), suggesting that the randomness inherent in the Extra Tree algorithm itself is well balanced, and that the predicted outcomes should be considered of high confidence.

**Fig. 4:**
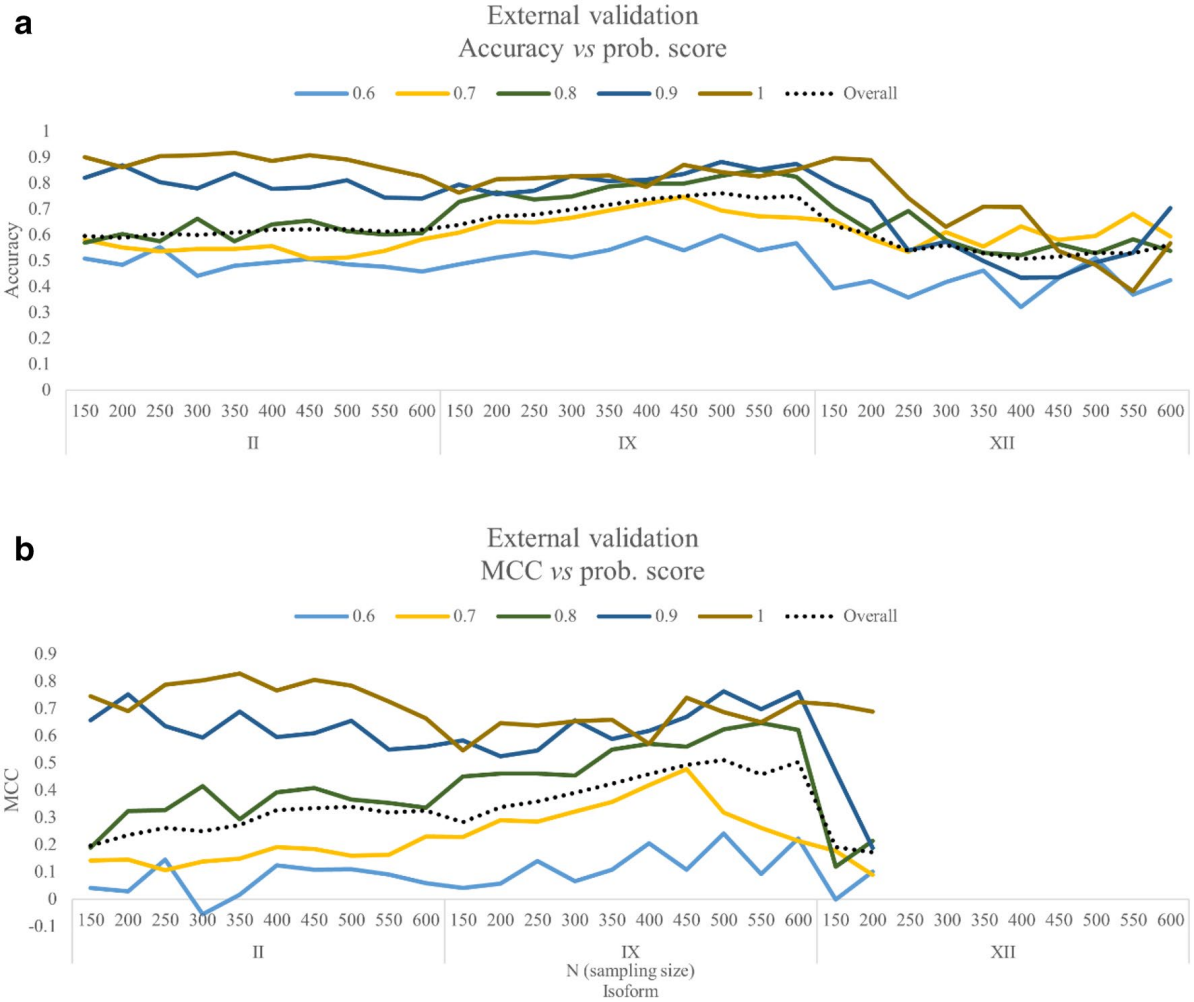
External validation of the Extra Tree models: graphical representation of **a** *accuracy* and **b** *MCC* at different values of the probability score. The overall *accuracy* and *MCC* values for all probability label are reported as a black dotted line. Missing datapoints in panel B correspond to validation datasets were no “inactive” instance could be found, and therefore *MCC* could not be calculated

For each isoform, the validation results at different *N* sampling sizes (black dotted line), and the values stratified *per* the different probability score (colored lines) are reported in terms of *accuracy* and *MCC* in Fig. 4 (panels a and b), respectively. Data points for the hCA XII isoform are missing at sampling sizes (*N*) higher than 250 due to the absence of molecules with bioactivity higher than 100 nM (“inactive” instances) in the validation dataset.

Interestingly, we observed that the *accuracy* and *MCC* values increase for all isoforms and groups, according to confidence level in the label prediction. The same trends could be observed also for *precision* and *recall* metrics (Additional file 4: Figure S2). This clearly shows that the use of probability scores to estimate the reliability of the predictions significantly reduced the number of FP and FN in the performed study.

As shown in Fig. 4 (panels a and b), models validation provided similar trends for hCA II and IX in terms of *accuracy* and *MCC*, the best results being at sampling sizes *N* = 350 and *N* = 450, respectively (see Table 5). Different results were obtained for hCA XII, in which the low number of activity records labeled as “inactive” forced us to choose small values of *N* for this isoform. Notwithstanding, hCA XII models trained with a sampling size equal to 150 allowed us to obtain *accuracy* and *MCC* scores of 0.90 and 0.71, respectively, when predictions with the highest probability score were considered (see Table 5). Remarkably, these values are superior to those obtained with the “traditional method” (see Table 4). These results show that although the proposed model was built with only 300 instances, with activities below 1.98 nM or above 397 nM (*N* = 150, see Table 3), it was able to outperform “traditional models” built on a larger dataset of molecules.

**Table 5:** *Accuracy*, *MCC*, *precision* and *recall* values for best models in the external validation phase

Probability score	Overall	0.6	0.7	0.8	0.9	1
*hCA II, N* *= 350*						
TP	782	151	223	131	150	127
FN	741	279	228	167	48	18
FP	204	70	103	17	5	9
TN	683	185	192	110	125	70
Accuracy	*0.61*	*0.49*	*0.56*	*0.57*	*0.84*	*0.88*
MCC	*0.28*	*0.08*	*0.14*	*0.29*	*0.70*	*0.75*
Precision	*0.79*	*0.68*	*0.68*	*0.89*	*0.97*	*0.93*
Recall	*0.51*	*0.35*	*0.49*	*0.44*	*0.76*	*0.88*
*hCA IX, N* *= 450*						
TP	326	46	103	75	59	43
FN	110	46	29	15	13	6
FP	61	15	20	11	8	7
TN	212	38	58	32	46	37
Accuracy	*0.76*	*0.58*	*0.77*	*0.80*	*0.83*	*0.86*
MCC	*0.51*	*0.21*	*0.51*	*0.57*	*0.67*	*0.72*
Precision	*0.84*	*0.75*	*0.84*	*0.87*	*0.88*	*0.86*
Recall	*0.75*	*0.50*	*0.78*	*0.83*	*0.82*	*0.88*
*hCA XII*, *N* *= 150*						
TP	524	88	183	134	55	64
FN	271	94	116	35	20	6
FP	32	7	13	7	4	1
TN	63	20	12	4	19	8
Accuracy	*0.66*	*0.52*	*0.60*	*0.77*	*0.76*	*0.91*
MCC	*0.21*	*0.15*	*0.05*	*0.09*	*0.48*	*0.67*
Precision	*0.94*	*0.93*	*0.93*	*0.95*	*0.93*	*0.98*
Recall	*0.66*	*0.48*	*0.61*	*0.79*	*0.73*	*0.91*

Results are averaged on 10 independent experiments and then rounded to the nearest integer value. Performance metrics are reported in italics

*TP* true positives, *FN* false negatives, *FP* false positives and *TN* true negatives

Finally, we investigated whether the obtained performances could be dependent on the chemical similarity between molecules in the training and validation datasets. To do this, we first extracted two subsets of molecules from the validation dataset, according to the degree of 2D similarity with the compounds in the training dataset. Ligands similarity was calculated by using the RDKit Atom Pair fingerprints (*APfp*) (see Methods), which are considered among the best types of fingerprints to correctly rank closely related analogues [32]. A Tanimoto coefficient (Tc) equal to 0.336 was used as a threshold to define the subsets containing molecules with either high (Tc_*APfp*_ ≥ 0.336) or low (Tc_*APfp*_ < 0.336) similarity to training dataset instances, respectively, as reported in the RDKit documentation. Then, we compared the *accuracy*, *MCC*, *precision* and *recall* values at probability scores equal to 1.0 for each subset, as shown in Table 6. The complete results of this analysis can be found in Additional file 4: Table S10. Interestingly, these analyses provided similar levels of prediction performance for both datasets on hCA II models, the obtained *MCC* scores being 0.75 for both subsets. Different results were obtained for hCA IX models, which provided *MCC* scores of 0.58 and 0.89 in the predictions of “similar” and “not similar” datasets, respectively, and for hCA XII with *MCC* scores of 0.48 and 0.76 in the “similar” and “not similar” dataset, respectively.

**Table 6:** Results of the validation phase with probability score equal to 1.0. Performance prediction statistics are reported for the two subsets (“similar” and “not similar”) extracted from the validation dataset

	TP	FN	FP	TN	Accuracy	MCC	Precision	Recall
*hCA II, N* = *350*								
Not similar	112	17	6	58	*0.88*	*0.75*	*0.87*	*0.95*
Similar	15	1	3	12	*0.87*	*0.75*	*0.94*	*0.83*
*hCA IX, N* = *450*								
Not similar	26	1	1	12	*0.95*	*0.89*	*0.96*	*0.96*
Similar	17	5	6	25	*0.79*	*0.58*	*0.77*	*0.74*
*hCA XII, N* = *150*								
Not similar	19	3	0	6	*0.89*	*0.76*	*0.86*	*1.00*
Similar	45	3	1	2	*0.92*	*0.48*	*0.94*	*0.98*

Similarity with respect to training dataset was calculated to select similar (“similar”) or dissimilar (“not similar”) subsets. Performance metrics are reported in italics

Altogether, results of the performed analyses show that models built with the flexible bioactivity threshold sampling method performed well, both in the training and testing phases. Moreover, the accuracy and *MCC* scores achieved in the prediction performances proves that models are able to generalize outside the initial dataset characteristics, and that the use of probability scores allows to refine results and drastically reduce the number of inaccurate predictions. The application of probability scores can also help assessing model confidence in the predictions of molecules with moderate or intermediate activities against agiven isoform, which are more difficult to classify. In the light of this, we envisage that such a score can in principle improve the prediction performance also in virtual screening conditions, and thus help prioritizing compounds for experimental testing.

Python scripts used to perform all training, testing and validation phases are made available as Additional file 5 and Additional file 6. The KNIME workflow used to analyze results is available as Additional file 7.

### Selectivity profiling

Having developed a model able to accurately discriminate active from inactive compounds, we then moved on evaluating whether the sequential application of our models was able to predict the known selectivity profile of ligands for the three hCA isoforms, *i.e.* II *vs* IX and II *vs* XII. In line with previous investigations [26, 33], we defined the selectivity profile of a compound by combining activity labels predicted on binary models on each hCA isoform.

Table 7 shows the outcomes of activity models and the final selectivity profiles thus determined. Therapeutically relevant classes (molecules predicted to be selective for either hCA IX or XII over hCA II) are highlighted in bold.

**Table 7:** Activity predictions and the corresponding selectivity profiles, potentially obtainable by using the binary activity models

Predicted label	hCA II	
	Active	Inactive
*hCA IX*		
Active	Non selective	Selective for hCA IX over hCA II
Inactive	Selective for hCA II over hCA IX	Non selective
*hCA XII*		
Active	Non selective	Selective for hCA XII over hCA II
Inactive	Selective for hCA II over hCA XII	Non selective

**Fig. 5:**
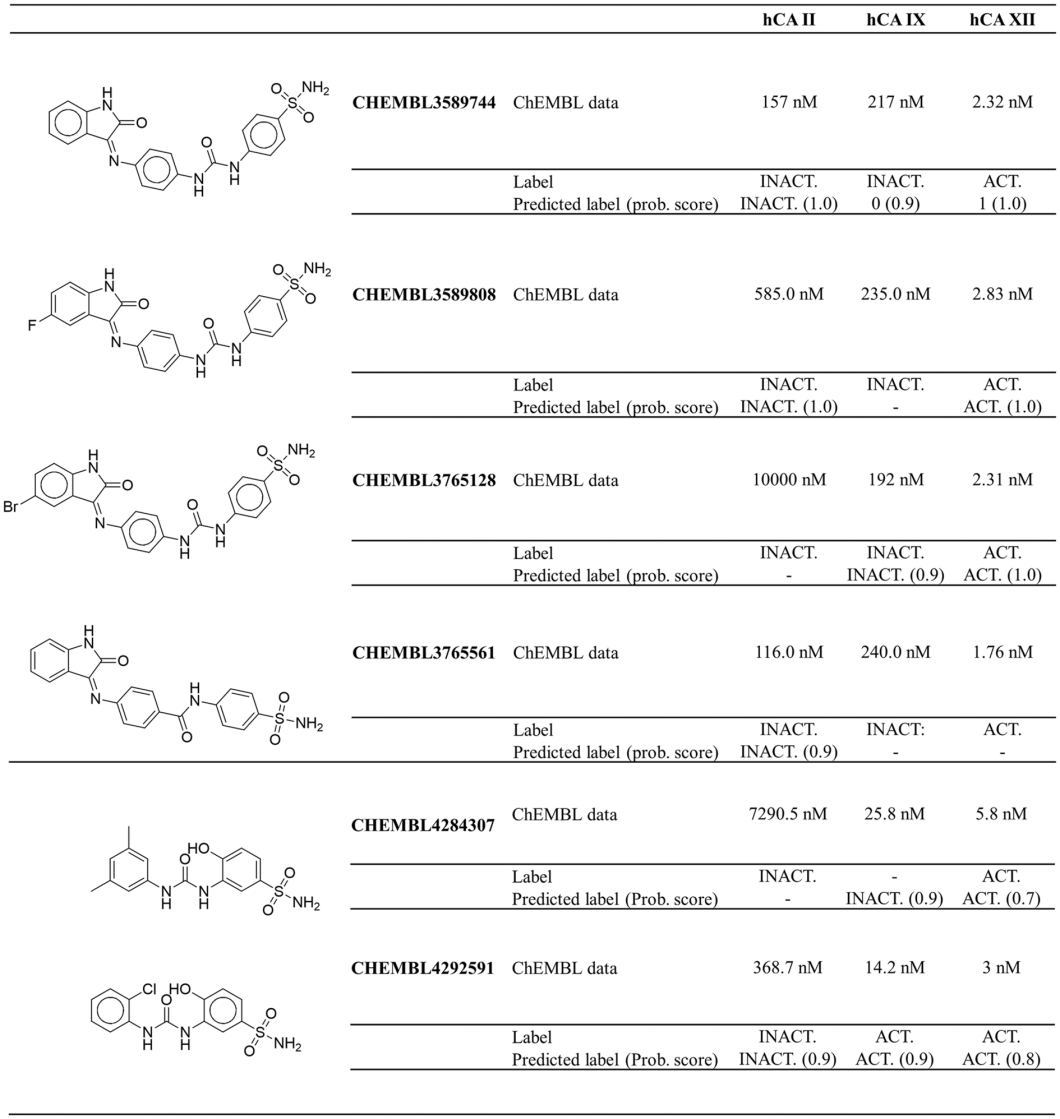
Examples of the activity predictions on a test set of molecules with reported activities on all investigated isoforms

To evaluate whether the sequential application of separate hCA binary models was able to correctly predict the selectivity profile of the ligands in the dataset, the results previously obtained in the validation phase (see Table 5) were analyzed. Figure 5 reports few examples of the compounds resulting from the applied machine learning models. In the reported examples, the ability of the Extra Tree models to recognize selective inhibitors out of a series of related compounds is highlighted. In particular, the first four examples (*i.e.*, CHEMBL3589744, CHEMBL3589808, CHEMBL3765128 and CHEMBL3765561) in Fig. 5 were correctly recognized as weak hCA II inhibitors, *i.e.* belonging to the inactive class, according to the defined thresholds. Moreover, activity predictions made for hCA IX and hCA XII were also correct, identifying these molecules as potent and selective hCA XII inhibitors. Missing predictions for some models are due to the fact that the molecules were sampled in the training dataset for that specific isoform.

Interestingly, our models were also able to correctly predict the different activity profile of molecules sharing a common scaffold, as reported for the pair of compounds CHEMBL4284307 and CHEMBL4292591. As shown in the lower part of Fig. 5, these molecules present different substituents on the phenyl ring, which in turn account for different molecular descriptors. Nevertheless, the molecules were correctly predicted to be selective for isoforms IX and XII, in agreement with experimental data reported in the ChEMBL database.

These examples show that our models, although being trained on very focused and small groups, are not strictly related to the activity values of the initial datasets (see Table 3). Indeed, they proved to be able to generalize and identify the features that make the "active" molecules (*i.e.*, strong binders), different from the "inactives" (*i.e.*, very weak, or not binders). Such a feature makes our approach appealing to be potentially applied also to other target families with a limited number of reported modulators, and for screening commercial databases to retain molecules that are predicted to be active on the isoform(s), or target(s), of interest.

## Conclusions

In this work, we trained a series of predictive machine learning classification models from bioactivity data reported on hCA II, hCA IX and hCA XII within ChEMBL. In particular, we first downloaded activity records related to the human Carbonic anhydrase isoforms II, IX and XII from the ChEMBL database (release 26, accessed on March 20th, 2020), and then processed them to obtain an initial dataset of high confidence data. We calculated a set of 92 low-correlated molecular descriptors to characterize each molecule. We then designed a sampling procedure to build balanced active/inactive classes with the *N* best and worst compounds according to activity values. A total of 360 models were built for each of the three hCA isoforms, using 12 sampling sizes, 10 different classification algorithms and three feature selection methods. Afterwards, we evaluated the model performances using a tenfold cross-validation training and testing phases on a dataset previously unseen by the model, repeated for 10 independent experiments to ensure consistent labels. Moreover, the models built using the best performing algorithm (Extra Tree) were trained and used to classify a validation dataset based on molecules not sampled by the chosen flexible sampling method.

A probability score, which was calculated and averaged for each label, proved to be an efficient scoring metrics to rank results and to reduce the percentage of mispredictions. Afterwards, we identified the best sampling sizes for models built on each isoform, which ensured excellent performances, both on molecules of the training and test datasets, but most importantly on molecules of the validation dataset whose bioactivity values fall outside of the ranges sampled by our flexible threshold method.

Finally, we discussed the possibility to use the binary activity models built on each separated isoform to predict the selectivity profile of a set of previously unseen molecules, and discussed six examples from our validation dataset based on different chemical scaffolds. This allowed us to demonstrate that the appropriate combination of activity labels enabled us to predict in the correct selectivity class similar molecules bearing different substituents. Such a feature is, for example, highly relevant in view of screening novel ligands with the desired selectivity profile, whether they be close analogues or more chemically diverse.

These results show that the problem of predicting the selectivity profile against the three hCA isoforms can be broken down into a succession of binary models trained on highly focused data. The best-performing models trained to predict the activity labels can be in principle employed in an ultra-fast virtual screening, where molecules predicted with the highest probability scores can be confidently selected for further experimental testing. Altogether, these results allow us to conclude that the combination of activity labels to predict the selectivity profile, together with probability scoring of the label confidence, allows for informative and accurate predictions.

All datasets and workflows used in the analysis are made available within this article. This approach, which can be applied also to other targets with reported known ligands, can be therefore easily implemented in a workflow suitable for large virtual screening purposes.

## Methods

### Dataset preparation

The initial dataset of hCA bioactivities was extracted from ChEMBL release 26 (accessed on March 20th, 2020) [22]. A total of 36,832 K_i_, IC_50_ and K_d_ activity records on 12 different isoforms of hCA was downloaded. A KNIME [34] workflow was used to create the initial dataset. In particular, activity records were filtered to retain only values reported with nanomolar standard unit, and a standard relation type corresponding to “ = ” (certain data) or “ > ” (automatically classified as inactive). Moreover, records with activity values not reported as IC_50_ or K_i_ for the hCA isoforms II, IX and XII were removed. The applied filtering criteria allowed the generation of a dataset of 18,857 records for 6396 unique molecules.

The RDKit library was used to preprocess molecules [29], by converting them to SMILES strings. Finally, the “*RDKit Structure Filter*” node was used to retain only molecules with a primary sulfonamide as Zinc Binding Group (ZBG). Mean and standard deviation were calculated for the molecules with more than one activity value reported on the same target. Molecules were retained if the standard deviation resulted less than 20% of original mean value. Molecules with a standard deviation greater than 20% of the mean were also kept if they were reported with more than 5 records on the same target, but their activity was replaced with the mode of the reported values, in the final dataset.

For each molecule, RDKit was used to calculate 118 different molecular descriptors [35]. Moreover, a procedure to eliminate redundant features was implemented. In particular, a correlation matrix was calculated with the *corr()* function in Python. Different values of *Pearson Correlation Coefficient* (PCC) were investigated, and two additional datasets were created retaining only features that provided PCCs lower than 0.95 (*PCC* = *0.95* dataset), and 0.75 (*PCC* = *0.75* dataset), with 92 and 57 descriptors included, respectively. A list of the original 118 molecular descriptors, and the filtered *PCC* = *0.95* and *PCC* = *0.75* subsets can be found in Additional file 4: Table S11.

### Implementation of machine learning algorithms

For each isoform (hCA II, hCA IX, hCA XII), the activities were sorted in ascending order. Afterwards, “active” and “inactive” classes were built by sampling the first* N* (active class) and the last* N* (inactive class) molecules. Sampling size* N* was varied from 150 to 700, using a 50 unit increment. An additional label was assigned using pre-established fixed activity thresholds. In this case, molecules with an activity below 20 nM were labeled as “active”, while molecules with activities above 100 nM were labeled as “inactive”. These labels were then used to build a comparative set of models to evaluate differences in performance against the proposed flexible bioactivity threshold method used in this work.

Python *scikit-learn* modules [28] were used to build, fine-tune and validate all machine learning models. The list of 10 supervised classification algorithms chosen in this work is reported in Table 8.

**Table 8:** The 10 classification algorithms implemented in Python *scikit-learn* modules used in this study

Method	Model abbreviation
Logistic Regression	LR
Linear Discriminant Analysis	LDA
K-Nearest Neighbor	KNN
Decision Tree	CART
Naïve Bayes	NB
Support Vector Machine	SVM
Ada Boost	AB
Gradient Boosting	GBM
Random Forest	RF
Extra Trees	ET

The initial dataset was divided into the training and test datasets, using a 75:25 ratio. In the training phase, 75% of the total database was subjected to a tenfold cross-validation to test the baseline prediction. In this phase 10 stratified subsets were created, 9 of which were used to train the model and 1 to test results. This procedure was repeated by shuffling the subset used as test. Then, in the testing phase, the whole training dataset was used to train models, which were subsequently used to predict the 25% previously withheld testing dataset. 10 independent experiments were calculated and averaged in the final results.

Metrics used to validate model performance were *accuracy*, *precision*, *recall* (ranging from 0 to 1) and *Matthews Correlation Coefficient* (MCC) [36], ranging from − 1 to 1.

$$Accuracy = \frac{TP + TN}{{TP + TN + FP + FN}}$$

$$Precision = \frac{TP}{{TP + FP}}$$

$$Recall = \frac{TP}{{TP + FN}}$$

$$MCC = \frac{TP \times TN - FP \times FN}{{\sqrt {\left( {TP + FP} \right)\left( {TP + FN} \right)\left( {TN + FP} \right)\left( {TN + FN} \right)} }}$$

A value of *MCC* higher than 0.6 was considered to be indicative of a good performance of the applied machine learning model.

In order to calculate the number of true positives (TP), true negatives (TN), false positives (FP) and false negatives (FN), the positive class was set to be correspondent to the “active” class in the activity models.

Moreover, a probability value was calculated for every prediction, as implemented in algorithm functions in *scikit-learn, i.e.,* using the *predict_proba* function to estimate the confidence level of predicted results. The function returns a tuple of the type *[probability score for class A; probability score for class B]* for each label, where the total sum is 1. Only the highest value for the predicted label was retained. Probability scores range from 0 (lowest confidence) to 1 (highest confidence on the predicted label).

In the final step of external validation, models were trained on the 100% of the initial dataset, and then used to predict the activity label of an additional set of molecules extracted from ChEMBL with activities below 20 nM or above 100 nM and not present in the training or testing dataset. 10 independent experiments were carried out, each of which had a numerical label ("1" corresponding to the active class, and "0" corresponding to the inactive class) and a probability confidence score. Mean and standard deviations were calculated for both the activity labels and probability scores, which were then rounded to obtain the final prediction. Predicted classification labels were compared with the bioactivity values reported in the validation dataset. Molecules with a reported bioactivity below 20 nM were considered as TP if classified as active by the models, and as FN if classified as inactive. Conversely, molecules with a reported bioactivity above 100 nM were considered as FP if classified as active, and as TN if classified as inactive. Based on these values, *accuracy* and *MCC* scores were calculated.

Finally, two subsets were extracted from the validation dataset. First, molecules in the training and validation datasets were converted to Atom Pair fingerprints (*APfp*), using the RDKit Python modules [30]. Then, the 2D similarity between the molecules in the training and validation dataset was estimated through the use of the Tanimoto coefficient (Tc). For each isoform and *N* sampling size, the averaged Tc (Tc_*APfp*_) and standard deviations were calculated. Then, the validation dataset was grouped in two sets of similar (“*similar*” subset: mean Tc_*APfp*_  ≥ 0.336) and dissimilar (“*not similar*” subset: mean Tc_*APfp*_ < 0.336) molecules, according to the averaged similarity with ligands in the training set. The selected threshold of Tc_*APfp*_ allowed to select as similar only 10% of “drug-like” molecules, randomly selected from ChEMBL, according to RDKit documentation [31].

## Supplementary Information

**Additional file 1.** Dataset with original downloaded ChEMBL bioactivities for hCA II, IX and XII.

**Additional file 2.** KNIME workflow to generate the initial dataset.

**Additional file 3.** Initial dataset used for all machine learning experiments.

**Additional file 4.** Additional Tables and Figures.

**Additional file 5.** Python script to perform training and testing phases.

**Additional file 6.** Python script to perform the validation phase with similarity calculations.

**Additional file 7.** KNIME workflow to analyze results and generate output tables.

## Data Availability

The datasets supporting the conclusions of this article are included within the article (and its additional files).

## Abbreviations

AB: Ada Boost
APfp: Atom Pair fingerprint
CART: Decision Tree
CO_2_: Carbon dioxide
ET: Extra Trees
FN: False Negative
FP: False Positive
fp: Fingerprint
GBM: Gradient Boosting
hCA: Human Carbonic Anhydrase
IC_50_: Half maximal inhibitory concentration
K_i_: Inhibition constant
KNN: K-Nearest Neighbor
LB: Ligand-based
LDA: Linear Discriminant Analysis
LR: Logistic Regression
MCC: Matthews Correlation Coefficient
NB: Naïve Bayes
nM: Nanomolar
PCC: Pearson Correlation Coefficient
RF: Random Forest
SB: Structure-based
SelA: Selective for target A
SelB: Selective for target B
SVM: Support Vector Machine
Tc: Tanimoto coefficient
TN: True Negative
TP: True Positive
ZBG: Zinc Binding Group
