# Supplementary material for: Prediction of activity and selectivity profiles of human Carbonic Anhydrase inhibitors using machine learning classification models
Source: J Cheminform. 2021 Mar 6;13:18. doi: 10.1186/s13321-021-00499-y (PMC7937250; doi:10.1186/s13321-021-00499-y)
Supplement: Supplementary file 4 — Additional file 4. Additional Tables and Figures. [file 13321_2021_499_MOESM4_ESM.pdf]

Supporting Information

Prediction of activity and selectivity profiles of human Carbonic Anhydrase inhibitors using machine learning classification models

Annachiara Tinivella[1,2], Luca Pinzi[1], Giulio Rastelli[1]*

[1] *Department of Life Sciences, University of Modena and Reggio Emilia, Via Giuseppe Campi 103, 41125 Modena, Italy.*

[2] *Clinical and Experimental Medicine PhD Program, University of Modena and Reggio Emilia, Modena, Italy*

Table of contents

Additional tables

Table S1 pag. S2
Table S2 pag. S3
Table S3 pag. S6
Table S4 pag. S9
Table S5 pag. S12
Table S6 pag. S15
Table S7 pag. S16
Table S8 pag. S17
Table S9 pag. S18
Table S10 pag. S19
Table S11 pag. S20

Additional figures

Figure S1 pag S22
Figure S2 pag S23

Table S1. *Number of active and inactive compounds reported for each isoform, according to different activity thresholds.*

Class	hCA II	hCA IX	hCA XII
Active (< 10 nM)	1449	662	806
Intermediate (10-100 nM)	1472	915	602
Inctive (>100 nM)	1245	733	246
Active (< 20 nM)	1873	886	945
Intermediate (20-100 nM)	1048	691	463
Inctive (>100 nM)	1245	733	246
Active (< 20 nM)	1873	886	945
Intermediate (20-200 nM)	1319	943	509
Inctive (>200 nM)	974	481	200
Active (< 50 nM)	2425	1291	1185
Intermediate (50-250 nM)	871	622	286
Inctive (>250 nM)	870	397	183
Active (< 50 nM)	2425	1291	1185
Intermediate (50-500 nM)	1205	778	334
Inctive (>500 nM)	536	241	135
Active (< 100 nM)	2929	1587	1409
Intermediate (100-500 nM)	701	482	110
Inctive (>500 nM)	536	241	135
Active (< 100 nM)	2929	1587	1409
Intermediate (100-1000 nM)	918	558	155
Inctive (>1000 nM)	319	165	90

Table S2. *Results of the training phase on hCA isoforms II, IX and XII, obtained by using the flexible threshold sampling approach. The results are expressed as averaged accuracy on a 10-fold cross validation (with standard deviations). Different Pearson Correlation Coefficient (PCC) values were used to generate the models (no PCC, PCC = 0.95, PCC = 0.75).*

PCC	N	AB	CART	ET	GBM	KNN	LDA	LR	NB	RF	SVM
						hCA II					
-	150	0.87 ± 0.07	0.79 ± 0.09	0.91 ± 0.04	0.88 ± 0.04	0.82 ± 0.08	0.85 ± 0.06	0.78 ± 0.07	0.71 ± 0.11	0.89 ± 0.05	0.65 ± 0.07
-	200	0.87 ± 0.05	0.87 ± 0.04	0.87 ± 0.04	0.9 ± 0.05	0.81 ± 0.06	0.86 ± 0.05	0.82 ± 0.05	0.67 ± 0.07	0.86 ± 0.04	0.63 ± 0.04
-	250	0.86 ± 0.07	0.82 ± 0.07	0.89 ± 0.05	0.9 ± 0.04	0.82 ± 0.09	0.82 ± 0.06	0.83 ± 0.07	0.66 ± 0.07	0.86 ± 0.06	0.62 ± 0.03
-	300	0.86 ± 0.05	0.87 ± 0.05	0.88 ± 0.04	0.91 ± 0.04	0.77 ± 0.06	0.84 ± 0.06	0.85 ± 0.04	0.68 ± 0.08	0.87 ± 0.05	0.63 ± 0.05
-	350	0.85 ± 0.06	0.84 ± 0.08	0.9 ± 0.05	0.91 ± 0.06	0.8 ± 0.05	0.82 ± 0.07	0.83 ± 0.04	0.67 ± 0.06	0.88 ± 0.04	0.6 ± 0.04
-	400	0.85 ± 0.04	0.81 ± 0.03	0.89 ± 0.05	0.89 ± 0.05	0.77 ± 0.07	0.82 ± 0.05	0.84 ± 0.05	0.7 ± 0.06	0.89 ± 0.04	0.6 ± 0.04
-	450	0.83 ± 0.04	0.82 ± 0.04	0.9 ± 0.03	0.89 ± 0.04	0.77 ± 0.05	0.82 ± 0.04	0.8 ± 0.05	0.68 ± 0.06	0.85 ± 0.05	0.6 ± 0.04
-	500	0.84 ± 0.03	0.84 ± 0.06	0.89 ± 0.03	0.89 ± 0.04	0.78 ± 0.05	0.81 ± 0.04	0.82 ± 0.04	0.65 ± 0.06	0.87 ± 0.04	0.61 ± 0.02
-	550	0.85 ± 0.02	0.84 ± 0.03	0.9 ± 0.03	0.9 ± 0.03	0.81 ± 0.03	0.81 ± 0.05	0.83 ± 0.04	0.69 ± 0.06	0.87 ± 0.03	0.61 ± 0.04
-	600	0.83 ± 0.04	0.85 ± 0.05	0.88 ± 0.03	0.9 ± 0.04	0.79 ± 0.05	0.81 ± 0.04	0.82 ± 0.04	0.65 ± 0.05	0.88 ± 0.04	0.61 ± 0.04
-	650	0.82 ± 0.04	0.83 ± 0.04	0.89 ± 0.03	0.88 ± 0.02	0.78 ± 0.04	0.82 ± 0.03	0.82 ± 0.04	0.66 ± 0.04	0.87 ± 0.04	0.61 ± 0.04
-	700	0.79 ± 0.02	0.83 ± 0.03	0.88 ± 0.04	0.87 ± 0.03	0.76 ± 0.05	0.81 ± 0.03	0.8 ± 0.03	0.68 ± 0.04	0.86 ± 0.03	0.6 ± 0.03
0.75	150	0.85 ± 0.06	0.88 ± 0.08	0.89 ± 0.06	0.91 ± 0.06	0.83 ± 0.11	0.79 ± 0.06	0.78 ± 0.07	0.69 ± 0.12	0.9 ± 0.05	0.68 ± 0.07
0.75	200	0.85 ± 0.05	0.84 ± 0.07	0.89 ± 0.05	0.89 ± 0.04	0.79 ± 0.04	0.81 ± 0.06	0.79 ± 0.06	0.72 ± 0.06	0.87 ± 0.04	0.7 ± 0.08
0.75	250	0.84 ± 0.05	0.85 ± 0.06	0.91 ± 0.05	0.9 ± 0.06	0.84 ± 0.08	0.79 ± 0.06	0.78 ± 0.04	0.69 ± 0.05	0.89 ± 0.05	0.67 ± 0.03
0.75	300	0.84 ± 0.04	0.85 ± 0.08	0.9 ± 0.06	0.9 ± 0.03	0.8 ± 0.06	0.77 ± 0.05	0.77 ± 0.04	0.7 ± 0.04	0.91 ± 0.04	0.69 ± 0.05
0.75	350	0.84 ± 0.05	0.85 ± 0.06	0.9 ± 0.04	0.89 ± 0.06	0.83 ± 0.06	0.78 ± 0.07	0.79 ± 0.07	0.69 ± 0.05	0.89 ± 0.03	0.67 ± 0.05
0.75	400	0.85 ± 0.04	0.83 ± 0.05	0.9 ± 0.03	0.89 ± 0.04	0.81 ± 0.05	0.79 ± 0.06	0.79 ± 0.06	0.71 ± 0.04	0.88 ± 0.04	0.65 ± 0.04
0.75	450	0.81 ± 0.04	0.85 ± 0.04	0.9 ± 0.03	0.87 ± 0.03	0.83 ± 0.03	0.75 ± 0.04	0.76 ± 0.04	0.69 ± 0.05	0.87 ± 0.03	0.66 ± 0.05
0.75	500	0.8 ± 0.04	0.85 ± 0.05	0.9 ± 0.03	0.87 ± 0.04	0.83 ± 0.05	0.76 ± 0.05	0.77 ± 0.05	0.69 ± 0.06	0.87 ± 0.04	0.66 ± 0.02
0.75	550	0.8 ± 0.03	0.83 ± 0.04	0.89 ± 0.04	0.88 ± 0.03	0.81 ± 0.04	0.78 ± 0.06	0.78 ± 0.04	0.73 ± 0.05	0.88 ± 0.03	0.67 ± 0.06
0.75	600	0.81 ± 0.04	0.85 ± 0.03	0.9 ± 0.03	0.88 ± 0.03	0.82 ± 0.03	0.78 ± 0.04	0.78 ± 0.04	0.72 ± 0.04	0.87 ± 0.04	0.67 ± 0.04
0.75	650	0.8 ± 0.05	0.84 ± 0.04	0.9 ± 0.03	0.87 ± 0.03	0.8 ± 0.05	0.77 ± 0.04	0.77 ± 0.03	0.73 ± 0.05	0.89 ± 0.02	0.68 ± 0.05
0.75	700	0.79 ± 0.03	0.83 ± 0.02	0.88 ± 0.04	0.87 ± 0.03	0.8 ± 0.03	0.78 ± 0.02	0.77 ± 0.03	0.71 ± 0.03	0.86 ± 0.04	0.68 ± 0.03
0.95	150	0.86 ± 0.08	0.79 ± 0.07	0.91 ± 0.06	0.9 ± 0.04	0.89 ± 0.06	0.81 ± 0.09	0.8 ± 0.07	0.71 ± 0.11	0.91 ± 0.04	0.66 ± 0.08
0.95	200	0.89 ± 0.08	0.83 ± 0.06	0.9 ± 0.04	0.91 ± 0.04	0.8 ± 0.06	0.84 ± 0.06	0.82 ± 0.05	0.68 ± 0.06	0.86 ± 0.05	0.69 ± 0.07
0.95	250	0.85 ± 0.06	0.82 ± 0.06	0.91 ± 0.04	0.9 ± 0.05	0.82 ± 0.07	0.83 ± 0.09	0.82 ± 0.07	0.66 ± 0.06	0.92 ± 0.04	0.67 ± 0.04
0.95	300	0.85 ± 0.05	0.88 ± 0.05	0.9 ± 0.05	0.91 ± 0.04	0.82 ± 0.06	0.84 ± 0.05	0.83 ± 0.05	0.69 ± 0.07	0.89 ± 0.05	0.68 ± 0.05
0.95	350	0.86 ± 0.04	0.84 ± 0.07	0.9 ± 0.03	0.9 ± 0.06	0.83 ± 0.05	0.8 ± 0.05	0.81 ± 0.05	0.66 ± 0.07	0.88 ± 0.06	0.67 ± 0.05
0.95	400	0.85 ± 0.05	0.82 ± 0.04	0.92 ± 0.04	0.91 ± 0.03	0.84 ± 0.05	0.79 ± 0.05	0.82 ± 0.05	0.71 ± 0.04	0.89 ± 0.05	0.66 ± 0.05
0.95	450	0.82 ± 0.04	0.83 ± 0.03	0.89 ± 0.02	0.9 ± 0.04	0.84 ± 0.05	0.77 ± 0.05	0.79 ± 0.04	0.68 ± 0.05	0.88 ± 0.03	0.65 ± 0.05
0.95	500	0.82 ± 0.04	0.86 ± 0.04	0.89 ± 0.04	0.89 ± 0.04	0.83 ± 0.05	0.79 ± 0.03	0.8 ± 0.03	0.67 ± 0.06	0.87 ± 0.04	0.65 ± 0.02
0.95	550	0.84 ± 0.03	0.84 ± 0.03	0.9 ± 0.03	0.89 ± 0.04	0.83 ± 0.05	0.78 ± 0.05	0.8 ± 0.06	0.7 ± 0.06	0.86 ± 0.05	0.67 ± 0.05
0.95	600	0.83 ± 0.04	0.85 ± 0.03	0.9 ± 0.04	0.9 ± 0.04	0.83 ± 0.02	0.8 ± 0.03	0.8 ± 0.03	0.67 ± 0.04	0.88 ± 0.04	0.67 ± 0.04
0.95	650	0.84 ± 0.04	0.85 ± 0.04	0.89 ± 0.04	0.89 ± 0.03	0.83 ± 0.04	0.8 ± 0.04	0.79 ± 0.05	0.69 ± 0.05	0.87 ± 0.03	0.68 ± 0.05

PCC	N	AB	CART	ET	GBM	KNN	LDA	LR	NB	RF	SVM
0.95	700	0.8 ± 0.05	0.83 ± 0.03	0.89 ± 0.04	0.89 ± 0.04	0.81 ± 0.04	0.79 ± 0.03	0.8 ± 0.02	0.69 ± 0.04	0.88 ± 0.03	0.68 ± 0.03
						hCA IX					
-	150	0.8 ± 0.06	0.77 ± 0.07	0.84 ± 0.09	0.87 ± 0.06	0.8 ± 0.09	0.83 ± 0.07	0.77 ± 0.09	0.54 ± 0.04	0.81 ± 0.11	0.54 ± 0.04
-	200	0.8 ± 0.08	0.77 ± 0.07	0.88 ± 0.05	0.83 ± 0.05	0.79 ± 0.06	0.82 ± 0.06	0.79 ± 0.05	0.5 ± 0.07	0.85 ± 0.07	0.57 ± 0.03
-	250	0.8 ± 0.05	0.79 ± 0.08	0.86 ± 0.06	0.84 ± 0.07	0.81 ± 0.08	0.79 ± 0.07	0.8 ± 0.05	0.61 ± 0.07	0.85 ± 0.05	0.63 ± 0.06
-	300	0.8 ± 0.04	0.74 ± 0.07	0.84 ± 0.05	0.83 ± 0.04	0.75 ± 0.1	0.76 ± 0.07	0.77 ± 0.05	0.68 ± 0.05	0.79 ± 0.06	0.57 ± 0.03
-	350	0.78 ± 0.07	0.8 ± 0.08	0.81 ± 0.04	0.84 ± 0.06	0.73 ± 0.06	0.72 ± 0.05	0.73 ± 0.05	0.6 ± 0.04	0.8 ± 0.02	0.59 ± 0.03
-	400	0.74 ± 0.06	0.74 ± 0.06	0.8 ± 0.07	0.81 ± 0.08	0.71 ± 0.06	0.67 ± 0.07	0.66 ± 0.08	0.62 ± 0.06	0.75 ± 0.06	0.57 ± 0.03
-	450	0.75 ± 0.04	0.73 ± 0.06	0.79 ± 0.05	0.78 ± 0.04	0.7 ± 0.04	0.7 ± 0.05	0.71 ± 0.06	0.59 ± 0.06	0.75 ± 0.03	0.56 ± 0.04
-	500	0.73 ± 0.04	0.71 ± 0.03	0.82 ± 0.05	0.79 ± 0.04	0.67 ± 0.04	0.66 ± 0.04	0.66 ± 0.04	0.58 ± 0.04	0.77 ± 0.02	0.58 ± 0.01
-	550	0.73 ± 0.05	0.76 ± 0.03	0.8 ± 0.06	0.82 ± 0.05	0.72 ± 0.05	0.68 ± 0.07	0.68 ± 0.07	0.56 ± 0.05	0.79 ± 0.06	0.59 ± 0.03
-	600	0.75 ± 0.04	0.75 ± 0.03	0.82 ± 0.04	0.81 ± 0.05	0.7 ± 0.03	0.69 ± 0.02	0.69 ± 0.03	0.62 ± 0.04	0.78 ± 0.02	0.58 ± 0.04
-	650	0.75 ± 0.04	0.78 ± 0.04	0.82 ± 0.04	0.81 ± 0.02	0.72 ± 0.06	0.68 ± 0.04	0.69 ± 0.05	0.58 ± 0.04	0.8 ± 0.03	0.57 ± 0.03
-	700	0.75 ± 0.02	0.74 ± 0.05	0.81 ± 0.03	0.82 ± 0.03	0.71 ± 0.03	0.68 ± 0.03	0.68 ± 0.03	0.64 ± 0.03	0.8 ± 0.03	0.59 ± 0.02
0.75	150	0.78 ± 0.09	0.78 ± 0.08	0.84 ± 0.07	0.83 ± 0.08	0.83 ± 0.1	0.79 ± 0.08	0.78 ± 0.08	0.67 ± 0.11	0.81 ± 0.08	0.62 ± 0.05
0.75	200	0.81 ± 0.05	0.78 ± 0.05	0.86 ± 0.06	0.84 ± 0.06	0.82 ± 0.08	0.73 ± 0.08	0.74 ± 0.08	0.59 ± 0.05	0.81 ± 0.07	0.62 ± 0.04
0.75	250	0.78 ± 0.05	0.79 ± 0.06	0.86 ± 0.07	0.86 ± 0.05	0.83 ± 0.06	0.75 ± 0.06	0.74 ± 0.06	0.54 ± 0.04	0.83 ± 0.06	0.69 ± 0.05
0.75	300	0.78 ± 0.06	0.77 ± 0.06	0.82 ± 0.05	0.81 ± 0.06	0.78 ± 0.08	0.72 ± 0.07	0.71 ± 0.06	0.66 ± 0.04	0.82 ± 0.06	0.63 ± 0.05
0.75	350	0.76 ± 0.07	0.79 ± 0.05	0.83 ± 0.03	0.81 ± 0.06	0.77 ± 0.05	0.69 ± 0.07	0.7 ± 0.08	0.64 ± 0.07	0.82 ± 0.04	0.65 ± 0.04
0.75	400	0.74 ± 0.05	0.73 ± 0.07	0.83 ± 0.06	0.78 ± 0.06	0.73 ± 0.07	0.64 ± 0.07	0.64 ± 0.08	0.58 ± 0.06	0.77 ± 0.08	0.63 ± 0.04
0.75	450	0.73 ± 0.04	0.73 ± 0.04	0.8 ± 0.06	0.78 ± 0.05	0.75 ± 0.06	0.66 ± 0.08	0.67 ± 0.06	0.59 ± 0.07	0.77 ± 0.04	0.61 ± 0.04
0.75	500	0.7 ± 0.04	0.72 ± 0.03	0.79 ± 0.04	0.76 ± 0.02	0.71 ± 0.04	0.65 ± 0.03	0.65 ± 0.03	0.56 ± 0.08	0.77 ± 0.04	0.64 ± 0.03
0.75	550	0.73 ± 0.07	0.75 ± 0.06	0.8 ± 0.04	0.8 ± 0.06	0.75 ± 0.07	0.64 ± 0.07	0.65 ± 0.06	0.53 ± 0.03	0.79 ± 0.07	0.64 ± 0.04
0.75	600	0.74 ± 0.06	0.74 ± 0.04	0.81 ± 0.04	0.79 ± 0.06	0.74 ± 0.03	0.68 ± 0.03	0.67 ± 0.03	0.6 ± 0.03	0.79 ± 0.04	0.64 ± 0.05
0.75	650	0.73 ± 0.04	0.76 ± 0.05	0.83 ± 0.03	0.8 ± 0.02	0.75 ± 0.05	0.66 ± 0.06	0.65 ± 0.06	0.56 ± 0.06	0.79 ± 0.03	0.63 ± 0.03
0.75	700	0.75 ± 0.03	0.73 ± 0.05	0.81 ± 0.03	0.8 ± 0.04	0.73 ± 0.03	0.68 ± 0.04	0.68 ± 0.02	0.58 ± 0.05	0.8 ± 0.03	0.63 ± 0.02
0.95	150	0.81 ± 0.07	0.78 ± 0.12	0.83 ± 0.08	0.86 ± 0.06	0.84 ± 0.08	0.76 ± 0.09	0.78 ± 0.11	0.57 ± 0.06	0.82 ± 0.08	0.61 ± 0.05
0.95	200	0.82 ± 0.07	0.77 ± 0.07	0.86 ± 0.05	0.84 ± 0.04	0.83 ± 0.06	0.79 ± 0.06	0.77 ± 0.08	0.56 ± 0.08	0.83 ± 0.06	0.62 ± 0.04
0.95	250	0.8 ± 0.06	0.77 ± 0.05	0.86 ± 0.03	0.85 ± 0.05	0.84 ± 0.06	0.78 ± 0.05	0.77 ± 0.04	0.58 ± 0.07	0.84 ± 0.04	0.68 ± 0.06
0.95	300	0.77 ± 0.04	0.77 ± 0.05	0.84 ± 0.05	0.8 ± 0.05	0.81 ± 0.06	0.74 ± 0.06	0.75 ± 0.05	0.67 ± 0.05	0.8 ± 0.06	0.63 ± 0.04
0.95	350	0.77 ± 0.05	0.8 ± 0.07	0.84 ± 0.03	0.86 ± 0.05	0.8 ± 0.06	0.71 ± 0.04	0.72 ± 0.06	0.59 ± 0.04	0.83 ± 0.04	0.63 ± 0.05
0.95	400	0.78 ± 0.05	0.77 ± 0.06	0.84 ± 0.06	0.83 ± 0.05	0.76 ± 0.08	0.66 ± 0.08	0.65 ± 0.09	0.61 ± 0.07	0.8 ± 0.06	0.62 ± 0.03
0.95	450	0.76 ± 0.05	0.76 ± 0.04	0.82 ± 0.03	0.79 ± 0.04	0.78 ± 0.06	0.69 ± 0.07	0.69 ± 0.08	0.59 ± 0.07	0.78 ± 0.05	0.61 ± 0.03
0.95	500	0.73 ± 0.04	0.74 ± 0.05	0.81 ± 0.05	0.78 ± 0.04	0.74 ± 0.04	0.65 ± 0.04	0.65 ± 0.03	0.59 ± 0.06	0.78 ± 0.04	0.64 ± 0.03
0.95	550	0.75 ± 0.07	0.75 ± 0.03	0.82 ± 0.04	0.82 ± 0.05	0.77 ± 0.06	0.65 ± 0.07	0.66 ± 0.07	0.56 ± 0.04	0.81 ± 0.06	0.64 ± 0.04
0.95	600	0.73 ± 0.05	0.74 ± 0.04	0.82 ± 0.04	0.82 ± 0.06	0.76 ± 0.04	0.69 ± 0.03	0.69 ± 0.05	0.64 ± 0.04	0.82 ± 0.03	0.65 ± 0.04
0.95	650	0.74 ± 0.03	0.77 ± 0.05	0.83 ± 0.03	0.83 ± 0.03	0.76 ± 0.07	0.68 ± 0.06	0.66 ± 0.06	0.59 ± 0.06	0.81 ± 0.02	0.64 ± 0.02
0.95	700	0.76 ± 0.03	0.77 ± 0.04	0.82 ± 0.03	0.83 ± 0.04	0.76 ± 0.04	0.68 ± 0.04	0.68 ± 0.04	0.63 ± 0.03	0.81 ± 0.03	0.64 ± 0.02
						hCA XII					
-	150	0.9 ± 0.05	0.79 ± 0.07	0.9 ± 0.05	0.91 ± 0.05	0.81 ± 0.07	0.84 ± 0.06	0.91 ± 0.08	0.72 ± 0.06	0.88 ± 0.08	0.56 ± 0.04
-	200	0.84 ± 0.07	0.82 ± 0.09	0.87 ± 0.05	0.89 ± 0.05	0.78 ± 0.05	0.84 ± 0.06	0.83 ± 0.06	0.62 ± 0.07	0.86 ± 0.06	0.58 ± 0.03
-	250	0.8 ± 0.05	0.78 ± 0.04	0.85 ± 0.04	0.85 ± 0.06	0.75 ± 0.05	0.79 ± 0.07	0.83 ± 0.09	0.66 ± 0.07	0.81 ± 0.06	0.56 ± 0.06

PCC	N	AB	CART	ET	GBM	KNN	LDA	LR	NB	RF	SVM
-	300	0.79 ± 0.11	0.8 ± 0.06	0.86 ± 0.06	0.86 ± 0.07	0.76 ± 0.06	0.78 ± 0.05	0.76 ± 0.08	0.58 ± 0.11	0.82 ± 0.08	0.6 ± 0.04
-	350	0.76 ± 0.06	0.78 ± 0.08	0.85 ± 0.06	0.82 ± 0.05	0.73 ± 0.08	0.75 ± 0.06	0.76 ± 0.06	0.56 ± 0.06	0.82 ± 0.06	0.58 ± 0.04
-	400	0.75 ± 0.04	0.75 ± 0.05	0.8 ± 0.04	0.83 ± 0.03	0.72 ± 0.06	0.75 ± 0.05	0.77 ± 0.06	0.51 ± 0.04	0.78 ± 0.05	0.56 ± 0.04
-	450	0.72 ± 0.05	0.74 ± 0.05	0.81 ± 0.04	0.81 ± 0.05	0.72 ± 0.06	0.74 ± 0.04	0.75 ± 0.05	0.52 ± 0.04	0.78 ± 0.05	0.57 ± 0.04
-	500	0.74 ± 0.05	0.76 ± 0.05	0.8 ± 0.04	0.8 ± 0.05	0.73 ± 0.04	0.75 ± 0.04	0.77 ± 0.04	0.55 ± 0.04	0.76 ± 0.04	0.57 ± 0.02
-	550	0.74 ± 0.07	0.72 ± 0.05	0.78 ± 0.05	0.79 ± 0.06	0.69 ± 0.02	0.73 ± 0.05	0.76 ± 0.04	0.55 ± 0.05	0.78 ± 0.05	0.58 ± 0.03
-	600	0.77 ± 0.02	0.72 ± 0.05	0.79 ± 0.05	0.8 ± 0.03	0.71 ± 0.04	0.74 ± 0.03	0.76 ± 0.03	0.54 ± 0.04	0.75 ± 0.05	0.57 ± 0.03
-	650	0.73 ± 0.04	0.72 ± 0.04	0.77 ± 0.03	0.79 ± 0.03	0.72 ± 0.05	0.73 ± 0.04	0.74 ± 0.03	0.53 ± 0.03	0.77 ± 0.01	0.58 ± 0.03
-	700	0.7 ± 0.04	0.71 ± 0.03	0.78 ± 0.04	0.76 ± 0.03	0.71 ± 0.02	0.69 ± 0.02	0.71 ± 0.04	0.52 ± 0.04	0.74 ± 0.03	0.58 ± 0.02
0.75	150	0.88 ± 0.06	0.84 ± 0.06	0.92 ± 0.06	0.91 ± 0.07	0.86 ± 0.06	0.87 ± 0.04	0.84 ± 0.06	0.72 ± 0.09	0.88 ± 0.07	0.62 ± 0.06
0.75	200	0.85 ± 0.06	0.81 ± 0.09	0.89 ± 0.05	0.88 ± 0.05	0.85 ± 0.04	0.79 ± 0.04	0.8 ± 0.07	0.68 ± 0.05	0.88 ± 0.06	0.65 ± 0.05
0.75	250	0.82 ± 0.09	0.8 ± 0.08	0.82 ± 0.05	0.86 ± 0.05	0.78 ± 0.07	0.75 ± 0.07	0.76 ± 0.08	0.68 ± 0.06	0.84 ± 0.05	0.62 ± 0.06
0.75	300	0.75 ± 0.1	0.78 ± 0.08	0.89 ± 0.07	0.86 ± 0.07	0.81 ± 0.08	0.75 ± 0.11	0.77 ± 0.1	0.62 ± 0.05	0.86 ± 0.07	0.64 ± 0.05
0.75	350	0.74 ± 0.05	0.79 ± 0.05	0.83 ± 0.05	0.82 ± 0.05	0.78 ± 0.05	0.71 ± 0.07	0.71 ± 0.07	0.6 ± 0.05	0.83 ± 0.05	0.63 ± 0.05
0.75	400	0.74 ± 0.05	0.79 ± 0.06	0.84 ± 0.05	0.84 ± 0.04	0.76 ± 0.05	0.74 ± 0.05	0.75 ± 0.05	0.63 ± 0.06	0.81 ± 0.05	0.62 ± 0.04
0.75	450	0.73 ± 0.04	0.74 ± 0.05	0.81 ± 0.03	0.81 ± 0.04	0.73 ± 0.05	0.71 ± 0.07	0.72 ± 0.07	0.63 ± 0.03	0.78 ± 0.04	0.64 ± 0.05
0.75	500	0.74 ± 0.04	0.73 ± 0.02	0.82 ± 0.03	0.79 ± 0.03	0.73 ± 0.03	0.74 ± 0.06	0.76 ± 0.05	0.62 ± 0.04	0.82 ± 0.03	0.63 ± 0.03
0.75	550	0.72 ± 0.04	0.72 ± 0.07	0.8 ± 0.04	0.78 ± 0.06	0.71 ± 0.06	0.72 ± 0.04	0.71 ± 0.05	0.58 ± 0.03	0.78 ± 0.05	0.63 ± 0.05
0.75	600	0.71 ± 0.05	0.72 ± 0.04	0.79 ± 0.04	0.79 ± 0.03	0.72 ± 0.04	0.74 ± 0.04	0.74 ± 0.04	0.58 ± 0.04	0.79 ± 0.04	0.64 ± 0.04
0.75	650	0.71 ± 0.03	0.71 ± 0.03	0.77 ± 0.03	0.77 ± 0.03	0.71 ± 0.03	0.72 ± 0.05	0.73 ± 0.04	0.58 ± 0.03	0.78 ± 0.02	0.62 ± 0.03
0.75	700	0.69 ± 0.04	0.72 ± 0.04	0.78 ± 0.03	0.76 ± 0.04	0.73 ± 0.03	0.69 ± 0.04	0.69 ± 0.03	0.54 ± 0.03	0.76 ± 0.04	0.64 ± 0.02
0.95	150	0.87 ± 0.05	0.79 ± 0.08	0.89 ± 0.06	0.91 ± 0.06	0.85 ± 0.09	0.89 ± 0.08	0.87 ± 0.09	0.74 ± 0.06	0.88 ± 0.05	0.6 ± 0.05
0.95	200	0.86 ± 0.06	0.81 ± 0.08	0.92 ± 0.03	0.91 ± 0.05	0.84 ± 0.07	0.83 ± 0.05	0.81 ± 0.05	0.67 ± 0.04	0.85 ± 0.05	0.64 ± 0.05
0.95	250	0.79 ± 0.07	0.77 ± 0.07	0.83 ± 0.05	0.84 ± 0.06	0.8 ± 0.05	0.79 ± 0.06	0.79 ± 0.07	0.65 ± 0.04	0.83 ± 0.06	0.61 ± 0.09
0.95	300	0.78 ± 0.08	0.78 ± 0.07	0.87 ± 0.09	0.84 ± 0.06	0.82 ± 0.07	0.77 ± 0.07	0.77 ± 0.09	0.63 ± 0.08	0.84 ± 0.07	0.63 ± 0.06
0.95	350	0.76 ± 0.06	0.8 ± 0.03	0.82 ± 0.07	0.82 ± 0.05	0.79 ± 0.07	0.76 ± 0.05	0.73 ± 0.05	0.58 ± 0.06	0.82 ± 0.03	0.62 ± 0.04
0.95	400	0.76 ± 0.04	0.77 ± 0.05	0.82 ± 0.03	0.84 ± 0.04	0.76 ± 0.07	0.76 ± 0.03	0.75 ± 0.04	0.53 ± 0.05	0.79 ± 0.03	0.62 ± 0.05
0.95	450	0.72 ± 0.04	0.74 ± 0.05	0.79 ± 0.06	0.82 ± 0.05	0.77 ± 0.04	0.75 ± 0.04	0.75 ± 0.04	0.54 ± 0.04	0.78 ± 0.06	0.62 ± 0.06
0.95	500	0.76 ± 0.05	0.77 ± 0.04	0.79 ± 0.02	0.79 ± 0.03	0.78 ± 0.04	0.74 ± 0.04	0.75 ± 0.04	0.58 ± 0.04	0.79 ± 0.05	0.63 ± 0.03
0.95	550	0.74 ± 0.05	0.72 ± 0.05	0.79 ± 0.05	0.78 ± 0.05	0.73 ± 0.06	0.73 ± 0.04	0.73 ± 0.05	0.57 ± 0.04	0.78 ± 0.05	0.63 ± 0.04
0.95	600	0.75 ± 0.04	0.71 ± 0.04	0.79 ± 0.04	0.8 ± 0.02	0.75 ± 0.05	0.74 ± 0.03	0.76 ± 0.03	0.57 ± 0.06	0.77 ± 0.03	0.63 ± 0.04
0.95	650	0.74 ± 0.06	0.72 ± 0.03	0.78 ± 0.05	0.78 ± 0.02	0.73 ± 0.02	0.73 ± 0.04	0.74 ± 0.04	0.57 ± 0.04	0.78 ± 0.03	0.62 ± 0.03
0.95	700	0.72 ± 0.04	0.74 ± 0.03	0.78 ± 0.02	0.78 ± 0.03	0.75 ± 0.03	0.69 ± 0.02	0.71 ± 0.03	0.53 ± 0.03	0.77 ± 0.04	0.64 ± 0.02

Table S3. *Results of the training phase on hCA isoforms II, IX and XII, expressed as averaged Matthews Correlation Coefficient (MCC) on a 10-fold cross validation (with standard deviations). Different Pearson Correlation Coefficient (PCC) values were used to generate the models (no PCC, PCC = 0.95, PCC = 0.75).*

PCC	N	AB	CART	ET	GBM	KNN	LDA	LR	NB	RF	SVM
						hCA II					
-	150	0.75 ± 0.13	0.6 ± 0.19	0.84 ± 0.08	0.78 ± 0.09	0.65 ± 0.16	0.71 ± 0.12	0.56 ± 0.15	0.46 ± 0.21	0.8 ± 0.11	0.41 ± 0.12
-	200	0.75 ± 0.1	0.74 ± 0.09	0.74 ± 0.07	0.81 ± 0.09	0.64 ± 0.12	0.73 ± 0.11	0.64 ± 0.11	0.36 ± 0.15	0.72 ± 0.09	0.38 ± 0.07
-	250	0.73 ± 0.14	0.64 ± 0.15	0.8 ± 0.09	0.81 ± 0.09	0.65 ± 0.18	0.64 ± 0.13	0.67 ± 0.14	0.36 ± 0.14	0.73 ± 0.13	0.37 ± 0.06
-	300	0.73 ± 0.1	0.75 ± 0.09	0.77 ± 0.08	0.82 ± 0.08	0.55 ± 0.13	0.69 ± 0.13	0.7 ± 0.09	0.37 ± 0.16	0.74 ± 0.11	0.38 ± 0.08
-	350	0.71 ± 0.12	0.69 ± 0.16	0.8 ± 0.1	0.81 ± 0.11	0.6 ± 0.11	0.63 ± 0.14	0.67 ± 0.09	0.35 ± 0.13	0.76 ± 0.08	0.33 ± 0.07
-	400	0.7 ± 0.08	0.63 ± 0.06	0.78 ± 0.09	0.79 ± 0.1	0.54 ± 0.14	0.64 ± 0.11	0.67 ± 0.1	0.41 ± 0.11	0.77 ± 0.09	0.32 ± 0.09
-	450	0.67 ± 0.08	0.65 ± 0.08	0.79 ± 0.06	0.78 ± 0.07	0.54 ± 0.09	0.64 ± 0.07	0.6 ± 0.1	0.36 ± 0.12	0.71 ± 0.11	0.32 ± 0.1
-	500	0.68 ± 0.06	0.68 ± 0.12	0.77 ± 0.05	0.77 ± 0.08	0.56 ± 0.11	0.62 ± 0.08	0.63 ± 0.08	0.33 ± 0.13	0.74 ± 0.09	0.35 ± 0.03
-	550	0.7 ± 0.05	0.67 ± 0.07	0.8 ± 0.06	0.8 ± 0.06	0.63 ± 0.05	0.62 ± 0.1	0.67 ± 0.07	0.38 ± 0.13	0.74 ± 0.07	0.34 ± 0.08
-	600	0.67 ± 0.07	0.7 ± 0.09	0.77 ± 0.06	0.8 ± 0.08	0.58 ± 0.1	0.63 ± 0.08	0.65 ± 0.07	0.34 ± 0.1	0.77 ± 0.08	0.34 ± 0.09
-	650	0.64 ± 0.09	0.67 ± 0.09	0.78 ± 0.05	0.75 ± 0.04	0.57 ± 0.08	0.64 ± 0.07	0.63 ± 0.08	0.34 ± 0.08	0.74 ± 0.08	0.35 ± 0.07
-	700	0.58 ± 0.03	0.67 ± 0.07	0.77 ± 0.07	0.74 ± 0.06	0.53 ± 0.1	0.63 ± 0.06	0.61 ± 0.05	0.37 ± 0.08	0.73 ± 0.06	0.32 ± 0.06
0.75	150	0.72 ± 0.12	0.76 ± 0.16	0.8 ± 0.13	0.82 ± 0.12	0.67 ± 0.23	0.59 ± 0.12	0.57 ± 0.14	0.42 ± 0.25	0.8 ± 0.11	0.47 ± 0.12
0.75	200	0.71 ± 0.09	0.69 ± 0.13	0.8 ± 0.1	0.78 ± 0.09	0.6 ± 0.09	0.63 ± 0.12	0.58 ± 0.12	0.44 ± 0.12	0.75 ± 0.07	0.48 ± 0.13
0.75	250	0.69 ± 0.1	0.7 ± 0.12	0.83 ± 0.1	0.8 ± 0.11	0.69 ± 0.17	0.58 ± 0.11	0.57 ± 0.09	0.4 ± 0.1	0.78 ± 0.1	0.44 ± 0.05
0.75	300	0.69 ± 0.09	0.71 ± 0.15	0.8 ± 0.12	0.81 ± 0.07	0.61 ± 0.11	0.54 ± 0.11	0.55 ± 0.08	0.4 ± 0.09	0.82 ± 0.07	0.47 ± 0.09
0.75	350	0.68 ± 0.09	0.7 ± 0.13	0.8 ± 0.09	0.78 ± 0.11	0.66 ± 0.11	0.56 ± 0.13	0.59 ± 0.14	0.39 ± 0.1	0.77 ± 0.06	0.44 ± 0.08
0.75	400	0.7 ± 0.08	0.65 ± 0.09	0.81 ± 0.07	0.78 ± 0.08	0.62 ± 0.11	0.58 ± 0.12	0.59 ± 0.12	0.43 ± 0.09	0.77 ± 0.07	0.4 ± 0.08
0.75	450	0.63 ± 0.08	0.71 ± 0.09	0.81 ± 0.06	0.74 ± 0.06	0.67 ± 0.06	0.49 ± 0.07	0.52 ± 0.08	0.41 ± 0.09	0.74 ± 0.07	0.4 ± 0.09
0.75	500	0.61 ± 0.08	0.71 ± 0.09	0.81 ± 0.05	0.74 ± 0.07	0.66 ± 0.1	0.53 ± 0.1	0.55 ± 0.11	0.4 ± 0.13	0.74 ± 0.08	0.43 ± 0.04
0.75	550	0.61 ± 0.06	0.67 ± 0.07	0.77 ± 0.08	0.77 ± 0.07	0.63 ± 0.08	0.56 ± 0.11	0.56 ± 0.08	0.47 ± 0.09	0.76 ± 0.07	0.44 ± 0.12
0.75	600	0.62 ± 0.09	0.69 ± 0.07	0.8 ± 0.05	0.76 ± 0.06	0.64 ± 0.06	0.56 ± 0.08	0.57 ± 0.07	0.47 ± 0.08	0.75 ± 0.08	0.45 ± 0.08
0.75	650	0.59 ± 0.1	0.68 ± 0.07	0.8 ± 0.06	0.75 ± 0.06	0.61 ± 0.11	0.55 ± 0.08	0.55 ± 0.06	0.48 ± 0.09	0.77 ± 0.05	0.47 ± 0.08
0.75	700	0.58 ± 0.06	0.67 ± 0.05	0.77 ± 0.09	0.73 ± 0.05	0.6 ± 0.06	0.56 ± 0.05	0.55 ± 0.06	0.43 ± 0.07	0.72 ± 0.08	0.45 ± 0.06
0.95	150	0.73 ± 0.17	0.59 ± 0.13	0.82 ± 0.12	0.81 ± 0.08	0.78 ± 0.12	0.63 ± 0.19	0.6 ± 0.15	0.45 ± 0.23	0.82 ± 0.08	0.43 ± 0.14
0.95	200	0.78 ± 0.15	0.67 ± 0.13	0.81 ± 0.08	0.82 ± 0.09	0.62 ± 0.12	0.68 ± 0.13	0.64 ± 0.1	0.38 ± 0.13	0.73 ± 0.1	0.47 ± 0.12
0.95	250	0.71 ± 0.13	0.65 ± 0.12	0.84 ± 0.08	0.82 ± 0.09	0.64 ± 0.14	0.67 ± 0.18	0.65 ± 0.14	0.35 ± 0.12	0.85 ± 0.09	0.44 ± 0.06
0.95	300	0.71 ± 0.1	0.77 ± 0.09	0.8 ± 0.1	0.83 ± 0.08	0.65 ± 0.12	0.68 ± 0.09	0.66 ± 0.1	0.39 ± 0.13	0.79 ± 0.09	0.47 ± 0.08
0.95	350	0.72 ± 0.08	0.68 ± 0.13	0.81 ± 0.06	0.81 ± 0.12	0.67 ± 0.1	0.61 ± 0.09	0.63 ± 0.1	0.33 ± 0.15	0.76 ± 0.12	0.44 ± 0.09
0.95	400	0.69 ± 0.1	0.63 ± 0.08	0.84 ± 0.08	0.82 ± 0.06	0.68 ± 0.11	0.59 ± 0.11	0.63 ± 0.11	0.44 ± 0.08	0.77 ± 0.09	0.42 ± 0.09
0.95	450	0.65 ± 0.08	0.66 ± 0.06	0.78 ± 0.04	0.8 ± 0.07	0.69 ± 0.1	0.55 ± 0.1	0.57 ± 0.08	0.38 ± 0.1	0.77 ± 0.07	0.4 ± 0.1
0.95	500	0.64 ± 0.07	0.72 ± 0.07	0.78 ± 0.07	0.77 ± 0.08	0.66 ± 0.09	0.58 ± 0.05	0.6 ± 0.07	0.39 ± 0.11	0.75 ± 0.09	0.42 ± 0.05
0.95	550	0.68 ± 0.07	0.68 ± 0.07	0.8 ± 0.06	0.78 ± 0.08	0.66 ± 0.1	0.56 ± 0.1	0.6 ± 0.11	0.42 ± 0.11	0.71 ± 0.11	0.45 ± 0.1
0.95	600	0.67 ± 0.08	0.71 ± 0.07	0.79 ± 0.09	0.8 ± 0.07	0.66 ± 0.05	0.6 ± 0.07	0.59 ± 0.07	0.39 ± 0.1	0.77 ± 0.07	0.45 ± 0.07
0.95	650	0.67 ± 0.07	0.71 ± 0.08	0.79 ± 0.07	0.77 ± 0.06	0.66 ± 0.08	0.6 ± 0.09	0.59 ± 0.09	0.4 ± 0.1	0.75 ± 0.05	0.47 ± 0.08

PCC	N	AB	CART	ET	GBM	KNN	LDA	LR	NB	RF	SVM
0.95	700	0.6 ± 0.1	0.67 ± 0.06	0.78 ± 0.08	0.78 ± 0.08	0.62 ± 0.08	0.58 ± 0.05	0.6 ± 0.05	0.4 ± 0.07	0.76 ± 0.06	0.45 ± 0.05
						hCA IX					
-	150	0.62 ± 0.13	0.54 ± 0.14	0.7 ± 0.18	0.74 ± 0.12	0.6 ± 0.17	0.67 ± 0.14	0.56 ± 0.18	0.12 ± 0.12	0.63 ± 0.21	0.15 ± 0.15
-	200	0.6 ± 0.15	0.54 ± 0.15	0.76 ± 0.1	0.67 ± 0.1	0.59 ± 0.12	0.65 ± 0.12	0.6 ± 0.09	0.01 ± 0.21	0.7 ± 0.13	0.26 ± 0.08
-	250	0.6 ± 0.1	0.58 ± 0.16	0.72 ± 0.11	0.69 ± 0.13	0.62 ± 0.15	0.59 ± 0.14	0.6 ± 0.09	0.27 ± 0.13	0.71 ± 0.1	0.37 ± 0.11
-	300	0.6 ± 0.08	0.49 ± 0.14	0.69 ± 0.11	0.66 ± 0.09	0.5 ± 0.21	0.53 ± 0.14	0.54 ± 0.1	0.38 ± 0.09	0.59 ± 0.12	0.24 ± 0.08
-	350	0.55 ± 0.13	0.59 ± 0.16	0.63 ± 0.08	0.69 ± 0.11	0.46 ± 0.11	0.45 ± 0.11	0.47 ± 0.09	0.22 ± 0.08	0.6 ± 0.05	0.29 ± 0.09
-	400	0.49 ± 0.12	0.49 ± 0.11	0.6 ± 0.13	0.63 ± 0.15	0.43 ± 0.13	0.35 ± 0.13	0.33 ± 0.15	0.26 ± 0.14	0.5 ± 0.13	0.25 ± 0.08
-	450	0.5 ± 0.08	0.47 ± 0.12	0.58 ± 0.09	0.56 ± 0.08	0.4 ± 0.09	0.4 ± 0.1	0.41 ± 0.13	0.19 ± 0.13	0.51 ± 0.07	0.2 ± 0.11
-	500	0.46 ± 0.08	0.42 ± 0.07	0.63 ± 0.1	0.59 ± 0.07	0.35 ± 0.07	0.32 ± 0.08	0.32 ± 0.08	0.19 ± 0.09	0.54 ± 0.05	0.25 ± 0.03
-	550	0.46 ± 0.1	0.53 ± 0.07	0.61 ± 0.11	0.64 ± 0.1	0.44 ± 0.1	0.36 ± 0.14	0.37 ± 0.13	0.16 ± 0.12	0.57 ± 0.11	0.29 ± 0.07
-	600	0.5 ± 0.08	0.49 ± 0.06	0.65 ± 0.07	0.62 ± 0.1	0.4 ± 0.06	0.38 ± 0.04	0.38 ± 0.05	0.25 ± 0.09	0.57 ± 0.04	0.25 ± 0.1
-	650	0.5 ± 0.08	0.57 ± 0.09	0.65 ± 0.08	0.61 ± 0.04	0.44 ± 0.12	0.35 ± 0.07	0.37 ± 0.09	0.2 ± 0.1	0.6 ± 0.06	0.23 ± 0.07
-	700	0.5 ± 0.03	0.48 ± 0.09	0.62 ± 0.07	0.64 ± 0.06	0.42 ± 0.06	0.36 ± 0.07	0.37 ± 0.07	0.28 ± 0.07	0.61 ± 0.06	0.28 ± 0.06
0.75	150	0.58 ± 0.18	0.57 ± 0.15	0.7 ± 0.13	0.67 ± 0.16	0.67 ± 0.21	0.58 ± 0.17	0.57 ± 0.16	0.35 ± 0.23	0.64 ± 0.16	0.34 ± 0.11
0.75	200	0.63 ± 0.11	0.57 ± 0.11	0.73 ± 0.13	0.68 ± 0.12	0.65 ± 0.15	0.46 ± 0.15	0.49 ± 0.17	0.23 ± 0.13	0.63 ± 0.15	0.36 ± 0.05
0.75	250	0.57 ± 0.1	0.58 ± 0.12	0.73 ± 0.14	0.72 ± 0.11	0.68 ± 0.12	0.51 ± 0.11	0.49 ± 0.12	0.14 ± 0.12	0.67 ± 0.12	0.48 ± 0.08
0.75	300	0.56 ± 0.12	0.54 ± 0.11	0.65 ± 0.11	0.63 ± 0.12	0.57 ± 0.17	0.44 ± 0.15	0.42 ± 0.11	0.33 ± 0.07	0.65 ± 0.11	0.35 ± 0.1
0.75	350	0.53 ± 0.13	0.58 ± 0.09	0.67 ± 0.06	0.62 ± 0.12	0.54 ± 0.11	0.39 ± 0.14	0.41 ± 0.15	0.28 ± 0.14	0.64 ± 0.09	0.39 ± 0.09
0.75	400	0.48 ± 0.1	0.46 ± 0.13	0.66 ± 0.12	0.57 ± 0.12	0.47 ± 0.15	0.29 ± 0.14	0.28 ± 0.17	0.19 ± 0.17	0.54 ± 0.16	0.35 ± 0.07
0.75	450	0.46 ± 0.08	0.46 ± 0.09	0.61 ± 0.12	0.57 ± 0.1	0.5 ± 0.12	0.33 ± 0.16	0.34 ± 0.12	0.2 ± 0.14	0.55 ± 0.08	0.3 ± 0.11
0.75	500	0.41 ± 0.08	0.45 ± 0.06	0.58 ± 0.08	0.53 ± 0.04	0.43 ± 0.07	0.29 ± 0.06	0.3 ± 0.05	0.15 ± 0.17	0.54 ± 0.08	0.35 ± 0.05
0.75	550	0.46 ± 0.13	0.5 ± 0.12	0.61 ± 0.09	0.61 ± 0.12	0.5 ± 0.15	0.28 ± 0.14	0.3 ± 0.11	0.11 ± 0.13	0.58 ± 0.13	0.37 ± 0.09
0.75	600	0.47 ± 0.11	0.49 ± 0.08	0.63 ± 0.08	0.59 ± 0.13	0.48 ± 0.07	0.36 ± 0.06	0.35 ± 0.06	0.23 ± 0.08	0.59 ± 0.07	0.35 ± 0.09
0.75	650	0.47 ± 0.07	0.52 ± 0.1	0.66 ± 0.06	0.6 ± 0.05	0.49 ± 0.1	0.33 ± 0.12	0.3 ± 0.11	0.17 ± 0.13	0.58 ± 0.06	0.35 ± 0.06
0.75	700	0.49 ± 0.06	0.46 ± 0.1	0.63 ± 0.06	0.61 ± 0.08	0.47 ± 0.07	0.36 ± 0.07	0.36 ± 0.05	0.2 ± 0.11	0.61 ± 0.06	0.35 ± 0.06
0.95	150	0.62 ± 0.14	0.56 ± 0.23	0.68 ± 0.17	0.72 ± 0.11	0.68 ± 0.16	0.54 ± 0.18	0.57 ± 0.23	0.2 ± 0.17	0.64 ± 0.16	0.33 ± 0.11
0.95	200	0.64 ± 0.15	0.54 ± 0.14	0.73 ± 0.1	0.69 ± 0.09	0.67 ± 0.12	0.59 ± 0.12	0.54 ± 0.16	0.17 ± 0.22	0.67 ± 0.13	0.35 ± 0.07
0.95	250	0.61 ± 0.12	0.55 ± 0.1	0.72 ± 0.07	0.7 ± 0.1	0.69 ± 0.13	0.58 ± 0.1	0.54 ± 0.08	0.24 ± 0.15	0.7 ± 0.08	0.46 ± 0.09
0.95	300	0.54 ± 0.08	0.55 ± 0.1	0.69 ± 0.1	0.6 ± 0.1	0.61 ± 0.12	0.48 ± 0.11	0.51 ± 0.09	0.36 ± 0.1	0.61 ± 0.11	0.35 ± 0.09
0.95	350	0.55 ± 0.11	0.61 ± 0.14	0.68 ± 0.05	0.72 ± 0.09	0.6 ± 0.11	0.43 ± 0.08	0.44 ± 0.12	0.2 ± 0.09	0.67 ± 0.07	0.36 ± 0.12
0.95	400	0.56 ± 0.1	0.55 ± 0.13	0.68 ± 0.12	0.65 ± 0.11	0.52 ± 0.15	0.32 ± 0.16	0.31 ± 0.19	0.25 ± 0.16	0.59 ± 0.13	0.35 ± 0.06
0.95	450	0.52 ± 0.09	0.52 ± 0.08	0.65 ± 0.07	0.58 ± 0.08	0.56 ± 0.13	0.38 ± 0.13	0.39 ± 0.16	0.2 ± 0.15	0.57 ± 0.09	0.31 ± 0.08
0.95	500	0.46 ± 0.08	0.49 ± 0.09	0.63 ± 0.1	0.56 ± 0.08	0.48 ± 0.08	0.31 ± 0.08	0.3 ± 0.06	0.21 ± 0.13	0.57 ± 0.08	0.36 ± 0.04
0.95	550	0.5 ± 0.13	0.51 ± 0.07	0.65 ± 0.07	0.64 ± 0.11	0.53 ± 0.11	0.31 ± 0.14	0.32 ± 0.14	0.15 ± 0.1	0.62 ± 0.13	0.36 ± 0.09
0.95	600	0.47 ± 0.11	0.49 ± 0.08	0.65 ± 0.08	0.64 ± 0.12	0.53 ± 0.07	0.38 ± 0.05	0.38 ± 0.09	0.28 ± 0.08	0.64 ± 0.06	0.38 ± 0.08
0.95	650	0.48 ± 0.06	0.54 ± 0.11	0.67 ± 0.06	0.66 ± 0.06	0.51 ± 0.13	0.36 ± 0.11	0.33 ± 0.11	0.21 ± 0.13	0.63 ± 0.05	0.37 ± 0.05
0.95	700	0.52 ± 0.06	0.54 ± 0.07	0.64 ± 0.07	0.65 ± 0.08	0.52 ± 0.07	0.36 ± 0.08	0.37 ± 0.08	0.29 ± 0.05	0.63 ± 0.06	0.37 ± 0.05
						hCA XII					
-	150	0.81 ± 0.11	0.59 ± 0.15	0.81 ± 0.1	0.82 ± 0.11	0.63 ± 0.14	0.68 ± 0.12	0.83 ± 0.14	0.45 ± 0.13	0.77 ± 0.16	0.21 ± 0.14
-	200	0.69 ± 0.15	0.65 ± 0.17	0.75 ± 0.1	0.79 ± 0.11	0.56 ± 0.1	0.68 ± 0.13	0.65 ± 0.11	0.29 ± 0.14	0.73 ± 0.13	0.28 ± 0.06
-	250	0.61 ± 0.1	0.56 ± 0.07	0.71 ± 0.07	0.71 ± 0.11	0.51 ± 0.1	0.59 ± 0.14	0.66 ± 0.18	0.34 ± 0.14	0.62 ± 0.12	0.19 ± 0.2

PCC	N	AB	CART	ET	GBM	KNN	LDA	LR	NB	RF	SVM
-	300	0.58 ± 0.22	0.61 ± 0.12	0.74 ± 0.12	0.73 ± 0.14	0.53 ± 0.12	0.56 ± 0.1	0.53 ± 0.16	0.18 ± 0.23	0.65 ± 0.16	0.33 ± 0.08
-	350	0.53 ± 0.11	0.56 ± 0.15	0.71 ± 0.12	0.65 ± 0.1	0.46 ± 0.17	0.51 ± 0.12	0.52 ± 0.11	0.14 ± 0.15	0.65 ± 0.12	0.27 ± 0.11
-	400	0.5 ± 0.08	0.51 ± 0.1	0.61 ± 0.07	0.66 ± 0.07	0.45 ± 0.13	0.5 ± 0.11	0.55 ± 0.12	0.02 ± 0.14	0.57 ± 0.09	0.23 ± 0.11
-	450	0.45 ± 0.1	0.49 ± 0.1	0.62 ± 0.08	0.62 ± 0.1	0.44 ± 0.13	0.49 ± 0.08	0.5 ± 0.1	0.07 ± 0.1	0.56 ± 0.1	0.24 ± 0.09
-	500	0.49 ± 0.09	0.52 ± 0.1	0.61 ± 0.08	0.6 ± 0.09	0.46 ± 0.08	0.5 ± 0.07	0.54 ± 0.09	0.13 ± 0.11	0.52 ± 0.08	0.26 ± 0.05
-	550	0.48 ± 0.14	0.44 ± 0.11	0.57 ± 0.11	0.58 ± 0.13	0.38 ± 0.05	0.47 ± 0.1	0.51 ± 0.08	0.12 ± 0.1	0.56 ± 0.11	0.26 ± 0.06
-	600	0.53 ± 0.03	0.44 ± 0.11	0.58 ± 0.1	0.6 ± 0.05	0.43 ± 0.08	0.48 ± 0.06	0.52 ± 0.07	0.09 ± 0.11	0.51 ± 0.1	0.22 ± 0.09
-	650	0.46 ± 0.09	0.45 ± 0.07	0.55 ± 0.05	0.58 ± 0.07	0.44 ± 0.09	0.47 ± 0.07	0.48 ± 0.06	0.08 ± 0.07	0.55 ± 0.01	0.24 ± 0.08
-	700	0.41 ± 0.07	0.42 ± 0.06	0.57 ± 0.08	0.53 ± 0.06	0.42 ± 0.05	0.39 ± 0.05	0.43 ± 0.07	0.06 ± 0.11	0.49 ± 0.06	0.24 ± 0.05
0.75	150	0.76 ± 0.12	0.68 ± 0.13	0.84 ± 0.11	0.83 ± 0.14	0.73 ± 0.12	0.74 ± 0.08	0.69 ± 0.11	0.45 ± 0.17	0.77 ± 0.14	0.36 ± 0.14
0.75	200	0.7 ± 0.12	0.63 ± 0.18	0.78 ± 0.1	0.77 ± 0.09	0.7 ± 0.08	0.59 ± 0.09	0.6 ± 0.15	0.41 ± 0.09	0.76 ± 0.12	0.41 ± 0.08
0.75	250	0.64 ± 0.17	0.61 ± 0.15	0.66 ± 0.11	0.73 ± 0.09	0.56 ± 0.15	0.5 ± 0.15	0.52 ± 0.16	0.38 ± 0.12	0.7 ± 0.11	0.34 ± 0.14
0.75	300	0.51 ± 0.19	0.56 ± 0.15	0.78 ± 0.13	0.73 ± 0.13	0.62 ± 0.16	0.5 ± 0.21	0.54 ± 0.2	0.28 ± 0.11	0.72 ± 0.14	0.38 ± 0.11
0.75	350	0.48 ± 0.09	0.58 ± 0.1	0.66 ± 0.1	0.65 ± 0.09	0.56 ± 0.11	0.42 ± 0.15	0.43 ± 0.15	0.28 ± 0.11	0.66 ± 0.09	0.37 ± 0.1
0.75	400	0.47 ± 0.09	0.6 ± 0.11	0.68 ± 0.09	0.69 ± 0.09	0.52 ± 0.1	0.48 ± 0.1	0.5 ± 0.11	0.28 ± 0.12	0.62 ± 0.11	0.34 ± 0.1
0.75	450	0.46 ± 0.07	0.48 ± 0.1	0.62 ± 0.06	0.62 ± 0.08	0.45 ± 0.1	0.42 ± 0.14	0.44 ± 0.14	0.32 ± 0.08	0.57 ± 0.09	0.37 ± 0.09
0.75	500	0.47 ± 0.08	0.46 ± 0.04	0.64 ± 0.06	0.59 ± 0.05	0.47 ± 0.07	0.49 ± 0.12	0.52 ± 0.09	0.26 ± 0.09	0.63 ± 0.07	0.34 ± 0.07
0.75	550	0.44 ± 0.07	0.44 ± 0.15	0.6 ± 0.09	0.56 ± 0.11	0.41 ± 0.11	0.44 ± 0.09	0.43 ± 0.11	0.21 ± 0.08	0.56 ± 0.11	0.34 ± 0.1
0.75	600	0.41 ± 0.09	0.44 ± 0.09	0.57 ± 0.07	0.59 ± 0.05	0.45 ± 0.07	0.48 ± 0.08	0.48 ± 0.09	0.19 ± 0.1	0.59 ± 0.09	0.35 ± 0.1
0.75	650	0.43 ± 0.06	0.43 ± 0.06	0.54 ± 0.06	0.53 ± 0.06	0.42 ± 0.05	0.44 ± 0.09	0.46 ± 0.08	0.22 ± 0.05	0.57 ± 0.05	0.31 ± 0.07
0.75	700	0.38 ± 0.07	0.44 ± 0.08	0.55 ± 0.06	0.52 ± 0.08	0.47 ± 0.06	0.37 ± 0.07	0.38 ± 0.06	0.13 ± 0.08	0.51 ± 0.07	0.34 ± 0.05
0.95	150	0.75 ± 0.11	0.6 ± 0.16	0.78 ± 0.12	0.83 ± 0.12	0.71 ± 0.18	0.78 ± 0.15	0.74 ± 0.18	0.51 ± 0.13	0.77 ± 0.11	0.32 ± 0.13
0.95	200	0.73 ± 0.11	0.63 ± 0.16	0.84 ± 0.05	0.82 ± 0.09	0.68 ± 0.15	0.66 ± 0.09	0.62 ± 0.1	0.4 ± 0.09	0.7 ± 0.1	0.39 ± 0.1
0.95	250	0.58 ± 0.14	0.55 ± 0.13	0.68 ± 0.1	0.69 ± 0.12	0.6 ± 0.09	0.6 ± 0.13	0.58 ± 0.13	0.34 ± 0.1	0.68 ± 0.12	0.32 ± 0.23
0.95	300	0.57 ± 0.17	0.56 ± 0.15	0.74 ± 0.18	0.69 ± 0.12	0.64 ± 0.14	0.55 ± 0.14	0.55 ± 0.17	0.27 ± 0.17	0.69 ± 0.14	0.37 ± 0.13
0.95	350	0.52 ± 0.12	0.61 ± 0.06	0.65 ± 0.13	0.65 ± 0.09	0.59 ± 0.14	0.53 ± 0.11	0.46 ± 0.1	0.17 ± 0.13	0.65 ± 0.07	0.36 ± 0.09
0.95	400	0.52 ± 0.09	0.54 ± 0.1	0.65 ± 0.06	0.68 ± 0.07	0.51 ± 0.14	0.52 ± 0.06	0.51 ± 0.09	0.08 ± 0.13	0.57 ± 0.05	0.34 ± 0.1
0.95	450	0.44 ± 0.08	0.48 ± 0.09	0.58 ± 0.12	0.64 ± 0.09	0.54 ± 0.09	0.51 ± 0.09	0.51 ± 0.09	0.1 ± 0.1	0.57 ± 0.12	0.34 ± 0.11
0.95	500	0.52 ± 0.11	0.53 ± 0.08	0.58 ± 0.05	0.59 ± 0.07	0.56 ± 0.08	0.49 ± 0.09	0.5 ± 0.08	0.23 ± 0.1	0.58 ± 0.1	0.37 ± 0.05
0.95	550	0.48 ± 0.11	0.45 ± 0.11	0.58 ± 0.1	0.57 ± 0.1	0.47 ± 0.12	0.46 ± 0.09	0.47 ± 0.09	0.16 ± 0.09	0.56 ± 0.1	0.35 ± 0.09
0.95	600	0.49 ± 0.09	0.43 ± 0.08	0.59 ± 0.07	0.6 ± 0.04	0.5 ± 0.1	0.48 ± 0.06	0.51 ± 0.06	0.17 ± 0.14	0.54 ± 0.06	0.33 ± 0.1
0.95	650	0.49 ± 0.11	0.44 ± 0.05	0.56 ± 0.1	0.57 ± 0.05	0.47 ± 0.04	0.45 ± 0.08	0.48 ± 0.08	0.15 ± 0.1	0.56 ± 0.06	0.32 ± 0.08
0.95	700	0.44 ± 0.07	0.48 ± 0.07	0.56 ± 0.03	0.57 ± 0.06	0.51 ± 0.05	0.39 ± 0.05	0.43 ± 0.07	0.07 ± 0.07	0.54 ± 0.07	0.36 ± 0.05

Table S4. *Results of the testing phase on hCA isoforms II, IX and XII, expressed as averaged accuracy on 10 experiments (with standard deviations). Different Pearson Correlation Coefficient (PCC) values were used to generate the models (no PCC, PCC = 0.95, PCC = 0.75).*

PCC	N	AB	CART	ET	GBM	KNN	LDA	LR	NB	RF	SVM
					hCA II						
-	150	0.83 ± 0.0	0.89 ± 0.01	0.91 ± 0.03	0.86 ± 0.01	0.84 ± 0.0	0.87 ± 0.0	0.83 ± 0.0	0.72 ± 0.0	0.88 ± 0.02	0.64 ± 0.0
-	200	0.88 ± 0.0	0.81 ± 0.02	0.86 ± 0.02	0.9 ± 0.01	0.78 ± 0.0	0.85 ± 0.0	0.78 ± 0.0	0.58 ± 0.0	0.86 ± 0.03	0.67 ± 0.0
-	250	0.9 ± 0.0	0.83 ± 0.01	0.92 ± 0.02	0.92 ± 0.0	0.82 ± 0.0	0.78 ± 0.0	0.8 ± 0.0	0.65 ± 0.0	0.9 ± 0.03	0.67 ± 0.0
-	300	0.84 ± 0.0	0.88 ± 0.01	0.88 ± 0.01	0.9 ± 0.0	0.81 ± 0.0	0.83 ± 0.0	0.83 ± 0.0	0.67 ± 0.0	0.86 ± 0.01	0.59 ± 0.0
-	350	0.84 ± 0.0	0.87 ± 0.01	0.91 ± 0.01	0.89 ± 0.01	0.83 ± 0.0	0.82 ± 0.0	0.78 ± 0.0	0.7 ± 0.0	0.89 ± 0.02	0.63 ± 0.0
-	400	0.85 ± 0.0	0.84 ± 0.01	0.88 ± 0.01	0.86 ± 0.0	0.81 ± 0.0	0.81 ± 0.0	0.81 ± 0.0	0.67 ± 0.0	0.87 ± 0.01	0.66 ± 0.0
-	450	0.86 ± 0.0	0.78 ± 0.01	0.9 ± 0.01	0.88 ± 0.0	0.8 ± 0.0	0.8 ± 0.0	0.78 ± 0.0	0.69 ± 0.0	0.88 ± 0.02	0.61 ± 0.0
-	500	0.79 ± 0.0	0.83 ± 0.01	0.87 ± 0.01	0.87 ± 0.0	0.75 ± 0.0	0.8 ± 0.0	0.78 ± 0.0	0.64 ± 0.0	0.87 ± 0.01	0.6 ± 0.0
-	550	0.85 ± 0.0	0.86 ± 0.02	0.92 ± 0.01	0.87 ± 0.0	0.76 ± 0.0	0.83 ± 0.0	0.82 ± 0.0	0.69 ± 0.0	0.89 ± 0.02	0.63 ± 0.0
-	600	0.81 ± 0.0	0.86 ± 0.01	0.88 ± 0.01	0.89 ± 0.0	0.75 ± 0.0	0.82 ± 0.0	0.81 ± 0.0	0.63 ± 0.0	0.86 ± 0.01	0.6 ± 0.0
-	650	0.84 ± 0.0	0.81 ± 0.01	0.88 ± 0.02	0.87 ± 0.0	0.78 ± 0.0	0.83 ± 0.0	0.82 ± 0.0	0.62 ± 0.0	0.86 ± 0.01	0.57 ± 0.0
-	700	0.77 ± 0.0	0.84 ± 0.01	0.89 ± 0.01	0.87 ± 0.0	0.79 ± 0.0	0.82 ± 0.0	0.8 ± 0.0	0.63 ± 0.0	0.87 ± 0.02	0.61 ± 0.0
0.75	150	0.87 ± 0.0	0.88 ± 0.01	0.9 ± 0.02	0.93 ± 0.0	0.89 ± 0.0	0.71 ± 0.0	0.77 ± 0.0	0.71 ± 0.0	0.87 ± 0.04	0.69 ± 0.0
0.75	200	0.82 ± 0.0	0.82 ± 0.0	0.87 ± 0.01	0.86 ± 0.01	0.83 ± 0.0	0.69 ± 0.0	0.67 ± 0.0	0.63 ± 0.0	0.86 ± 0.02	0.7 ± 0.0
0.75	250	0.9 ± 0.0	0.84 ± 0.01	0.91 ± 0.02	0.92 ± 0.0	0.85 ± 0.0	0.75 ± 0.0	0.78 ± 0.0	0.71 ± 0.0	0.9 ± 0.02	0.69 ± 0.0
0.75	300	0.81 ± 0.0	0.81 ± 0.01	0.9 ± 0.01	0.85 ± 0.0	0.83 ± 0.0	0.76 ± 0.0	0.77 ± 0.0	0.74 ± 0.0	0.86 ± 0.02	0.61 ± 0.0
0.75	350	0.82 ± 0.0	0.82 ± 0.01	0.91 ± 0.01	0.89 ± 0.0	0.85 ± 0.0	0.73 ± 0.0	0.73 ± 0.0	0.73 ± 0.0	0.9 ± 0.02	0.67 ± 0.0
0.75	400	0.81 ± 0.0	0.84 ± 0.01	0.89 ± 0.01	0.89 ± 0.0	0.83 ± 0.0	0.75 ± 0.0	0.76 ± 0.0	0.68 ± 0.0	0.88 ± 0.02	0.69 ± 0.0
0.75	450	0.84 ± 0.0	0.84 ± 0.01	0.91 ± 0.01	0.9 ± 0.0	0.81 ± 0.0	0.7 ± 0.0	0.71 ± 0.0	0.73 ± 0.0	0.89 ± 0.02	0.66 ± 0.0
0.75	500	0.79 ± 0.0	0.83 ± 0.01	0.87 ± 0.02	0.87 ± 0.0	0.78 ± 0.0	0.67 ± 0.0	0.69 ± 0.0	0.71 ± 0.0	0.85 ± 0.02	0.66 ± 0.0
0.75	550	0.8 ± 0.0	0.83 ± 0.01	0.91 ± 0.01	0.88 ± 0.0	0.79 ± 0.0	0.76 ± 0.0	0.76 ± 0.0	0.72 ± 0.0	0.89 ± 0.01	0.71 ± 0.0
0.75	600	0.79 ± 0.0	0.83 ± 0.01	0.89 ± 0.01	0.86 ± 0.0	0.79 ± 0.0	0.71 ± 0.0	0.73 ± 0.0	0.68 ± 0.0	0.87 ± 0.01	0.67 ± 0.0
0.75	650	0.82 ± 0.0	0.81 ± 0.01	0.88 ± 0.01	0.87 ± 0.0	0.76 ± 0.0	0.75 ± 0.0	0.75 ± 0.0	0.68 ± 0.0	0.87 ± 0.02	0.65 ± 0.0
0.75	700	0.78 ± 0.0	0.81 ± 0.01	0.89 ± 0.02	0.84 ± 0.0	0.8 ± 0.0	0.71 ± 0.0	0.71 ± 0.0	0.67 ± 0.0	0.87 ± 0.01	0.71 ± 0.0
0.95	150	0.84 ± 0.0	0.85 ± 0.02	0.89 ± 0.02	0.91 ± 0.0	0.97 ± 0.0	0.88 ± 0.0	0.83 ± 0.0	0.67 ± 0.0	0.87 ± 0.02	0.73 ± 0.0
0.95	200	0.85 ± 0.0	0.83 ± 0.03	0.9 ± 0.02	0.89 ± 0.02	0.86 ± 0.0	0.76 ± 0.0	0.74 ± 0.0	0.6 ± 0.0	0.84 ± 0.02	0.69 ± 0.0
0.95	250	0.94 ± 0.0	0.84 ± 0.02	0.91 ± 0.02	0.9 ± 0.0	0.86 ± 0.0	0.8 ± 0.0	0.77 ± 0.0	0.66 ± 0.0	0.89 ± 0.02	0.69 ± 0.0
0.95	300	0.82 ± 0.0	0.87 ± 0.01	0.88 ± 0.02	0.89 ± 0.0	0.88 ± 0.0	0.81 ± 0.0	0.8 ± 0.0	0.67 ± 0.0	0.86 ± 0.02	0.6 ± 0.0
0.95	350	0.83 ± 0.0	0.87 ± 0.01	0.91 ± 0.01	0.9 ± 0.0	0.85 ± 0.0	0.78 ± 0.0	0.79 ± 0.0	0.7 ± 0.0	0.89 ± 0.02	0.67 ± 0.0
0.95	400	0.85 ± 0.0	0.84 ± 0.01	0.89 ± 0.01	0.87 ± 0.0	0.83 ± 0.0	0.78 ± 0.0	0.8 ± 0.0	0.67 ± 0.0	0.87 ± 0.02	0.71 ± 0.0
0.95	450	0.85 ± 0.0	0.79 ± 0.01	0.91 ± 0.02	0.9 ± 0.0	0.82 ± 0.0	0.79 ± 0.0	0.77 ± 0.0	0.72 ± 0.0	0.89 ± 0.01	0.66 ± 0.0
0.95	500	0.78 ± 0.0	0.83 ± 0.01	0.88 ± 0.01	0.87 ± 0.0	0.8 ± 0.0	0.74 ± 0.0	0.74 ± 0.0	0.67 ± 0.0	0.87 ± 0.02	0.66 ± 0.0
0.95	550	0.82 ± 0.0	0.86 ± 0.01	0.91 ± 0.01	0.89 ± 0.0	0.82 ± 0.0	0.8 ± 0.0	0.8 ± 0.0	0.72 ± 0.0	0.9 ± 0.01	0.7 ± 0.0
0.95	600	0.81 ± 0.0	0.86 ± 0.01	0.89 ± 0.01	0.91 ± 0.0	0.8 ± 0.0	0.77 ± 0.0	0.79 ± 0.0	0.63 ± 0.0	0.86 ± 0.02	0.67 ± 0.0
0.95	650	0.8 ± 0.0	0.83 ± 0.01	0.89 ± 0.01	0.88 ± 0.0	0.8 ± 0.0	0.82 ± 0.0	0.8 ± 0.0	0.63 ± 0.0	0.87 ± 0.01	0.65 ± 0.0
0.95	700	0.83 ± 0.0	0.83 ± 0.01	0.89 ± 0.01	0.88 ± 0.0	0.84 ± 0.0	0.78 ± 0.0	0.78 ± 0.0	0.66 ± 0.0	0.88 ± 0.02	0.71 ± 0.0
					hCA IX						

PCC	N	AB	CART	ET	GBM	KNN	LDA	LR	NB	RF	SVM
-	150	0.81 ± 0.0	0.9 ± 0.02	0.88 ± 0.03	0.89 ± 0.01	0.88 ± 0.0	0.81 ± 0.0	0.79 ± 0.0	0.49 ± 0.0	0.85 ± 0.05	0.6 ± 0.0
-	200	0.77 ± 0.0	0.89 ± 0.01	0.86 ± 0.02	0.85 ± 0.01	0.78 ± 0.0	0.68 ± 0.0	0.72 ± 0.0	0.51 ± 0.0	0.85 ± 0.02	0.59 ± 0.0
-	250	0.82 ± 0.0	0.74 ± 0.01	0.82 ± 0.01	0.83 ± 0.0	0.77 ± 0.0	0.74 ± 0.0	0.74 ± 0.0	0.62 ± 0.0	0.8 ± 0.02	0.56 ± 0.0
-	300	0.81 ± 0.0	0.78 ± 0.02	0.87 ± 0.02	0.84 ± 0.0	0.78 ± 0.0	0.77 ± 0.0	0.78 ± 0.0	0.67 ± 0.0	0.83 ± 0.01	0.63 ± 0.0
-	350	0.79 ± 0.0	0.73 ± 0.02	0.8 ± 0.01	0.83 ± 0.0	0.73 ± 0.0	0.73 ± 0.0	0.69 ± 0.0	0.65 ± 0.0	0.78 ± 0.02	0.57 ± 0.0
-	400	0.78 ± 0.0	0.74 ± 0.01	0.82 ± 0.01	0.85 ± 0.0	0.78 ± 0.0	0.76 ± 0.0	0.79 ± 0.0	0.61 ± 0.0	0.8 ± 0.01	0.62 ± 0.0
-	450	0.79 ± 0.0	0.77 ± 0.01	0.84 ± 0.02	0.86 ± 0.01	0.77 ± 0.0	0.76 ± 0.0	0.75 ± 0.0	0.6 ± 0.0	0.82 ± 0.01	0.62 ± 0.0
-	500	0.81 ± 0.0	0.79 ± 0.01	0.86 ± 0.01	0.86 ± 0.0	0.77 ± 0.0	0.77 ± 0.0	0.76 ± 0.0	0.6 ± 0.0	0.84 ± 0.01	0.6 ± 0.0
-	550	0.77 ± 0.0	0.75 ± 0.01	0.81 ± 0.01	0.8 ± 0.0	0.69 ± 0.0	0.74 ± 0.0	0.73 ± 0.0	0.59 ± 0.0	0.78 ± 0.02	0.59 ± 0.0
-	600	0.74 ± 0.0	0.73 ± 0.01	0.78 ± 0.01	0.76 ± 0.0	0.67 ± 0.0	0.71 ± 0.0	0.71 ± 0.0	0.61 ± 0.0	0.76 ± 0.01	0.57 ± 0.0
-	650	0.74 ± 0.0	0.78 ± 0.01	0.81 ± 0.02	0.8 ± 0.0	0.69 ± 0.0	0.73 ± 0.0	0.73 ± 0.0	0.61 ± 0.0	0.78 ± 0.01	0.6 ± 0.0
-	700	0.71 ± 0.0	0.69 ± 0.01	0.8 ± 0.01	0.8 ± 0.0	0.68 ± 0.0	0.68 ± 0.0	0.69 ± 0.0	0.61 ± 0.0	0.78 ± 0.02	0.58 ± 0.0
0.75	150	0.79 ± 0.0	0.78 ± 0.03	0.89 ± 0.03	0.83 ± 0.01	0.77 ± 0.0	0.79 ± 0.0	0.75 ± 0.0	0.77 ± 0.0	0.84 ± 0.04	0.68 ± 0.0
0.75	200	0.81 ± 0.0	0.88 ± 0.01	0.87 ± 0.02	0.83 ± 0.0	0.8 ± 0.0	0.79 ± 0.0	0.76 ± 0.0	0.65 ± 0.0	0.84 ± 0.03	0.64 ± 0.0
0.75	250	0.77 ± 0.0	0.81 ± 0.02	0.82 ± 0.01	0.8 ± 0.01	0.8 ± 0.0	0.7 ± 0.0	0.69 ± 0.0	0.55 ± 0.0	0.81 ± 0.02	0.58 ± 0.0
0.75	300	0.79 ± 0.0	0.81 ± 0.0	0.87 ± 0.01	0.84 ± 0.0	0.83 ± 0.0	0.71 ± 0.0	0.77 ± 0.0	0.75 ± 0.0	0.84 ± 0.02	0.67 ± 0.0
0.75	350	0.75 ± 0.0	0.74 ± 0.01	0.8 ± 0.01	0.81 ± 0.0	0.74 ± 0.0	0.69 ± 0.0	0.69 ± 0.0	0.7 ± 0.0	0.8 ± 0.02	0.61 ± 0.0
0.75	400	0.75 ± 0.0	0.79 ± 0.01	0.83 ± 0.02	0.84 ± 0.0	0.78 ± 0.0	0.74 ± 0.0	0.73 ± 0.0	0.58 ± 0.0	0.82 ± 0.01	0.66 ± 0.0
0.75	450	0.74 ± 0.0	0.8 ± 0.01	0.83 ± 0.02	0.83 ± 0.0	0.84 ± 0.0	0.71 ± 0.0	0.71 ± 0.0	0.62 ± 0.0	0.84 ± 0.01	0.65 ± 0.0
0.75	500	0.76 ± 0.0	0.8 ± 0.01	0.87 ± 0.02	0.85 ± 0.0	0.81 ± 0.0	0.68 ± 0.0	0.68 ± 0.0	0.56 ± 0.0	0.86 ± 0.01	0.65 ± 0.0
0.75	550	0.77 ± 0.0	0.76 ± 0.01	0.84 ± 0.01	0.77 ± 0.0	0.75 ± 0.0	0.68 ± 0.0	0.66 ± 0.0	0.55 ± 0.0	0.82 ± 0.01	0.64 ± 0.0
0.75	600	0.68 ± 0.0	0.74 ± 0.01	0.79 ± 0.01	0.75 ± 0.0	0.75 ± 0.0	0.66 ± 0.0	0.67 ± 0.0	0.6 ± 0.0	0.78 ± 0.01	0.62 ± 0.0
0.75	650	0.73 ± 0.0	0.78 ± 0.01	0.81 ± 0.01	0.81 ± 0.0	0.76 ± 0.0	0.68 ± 0.0	0.66 ± 0.0	0.57 ± 0.0	0.8 ± 0.01	0.66 ± 0.0
0.75	700	0.69 ± 0.0	0.72 ± 0.0	0.81 ± 0.01	0.76 ± 0.0	0.73 ± 0.0	0.63 ± 0.0	0.63 ± 0.0	0.54 ± 0.0	0.78 ± 0.01	0.63 ± 0.0
0.95	150	0.77 ± 0.0	0.85 ± 0.03	0.89 ± 0.02	0.87 ± 0.01	0.87 ± 0.0	0.75 ± 0.0	0.76 ± 0.0	0.63 ± 0.0	0.88 ± 0.03	0.64 ± 0.0
0.95	200	0.75 ± 0.0	0.75 ± 0.03	0.88 ± 0.02	0.85 ± 0.01	0.87 ± 0.0	0.71 ± 0.0	0.72 ± 0.0	0.56 ± 0.0	0.85 ± 0.03	0.63 ± 0.0
0.95	250	0.79 ± 0.0	0.79 ± 0.02	0.84 ± 0.02	0.86 ± 0.01	0.82 ± 0.0	0.74 ± 0.0	0.71 ± 0.0	0.55 ± 0.0	0.81 ± 0.01	0.57 ± 0.0
0.95	300	0.83 ± 0.0	0.77 ± 0.01	0.87 ± 0.01	0.87 ± 0.0	0.85 ± 0.0	0.78 ± 0.0	0.77 ± 0.0	0.7 ± 0.0	0.85 ± 0.02	0.67 ± 0.0
0.95	350	0.79 ± 0.0	0.74 ± 0.01	0.82 ± 0.01	0.83 ± 0.0	0.77 ± 0.0	0.67 ± 0.0	0.69 ± 0.0	0.65 ± 0.0	0.82 ± 0.02	0.62 ± 0.0
0.95	400	0.81 ± 0.0	0.79 ± 0.01	0.83 ± 0.02	0.87 ± 0.0	0.79 ± 0.0	0.77 ± 0.0	0.78 ± 0.0	0.59 ± 0.0	0.82 ± 0.02	0.66 ± 0.0
0.95	450	0.76 ± 0.0	0.78 ± 0.01	0.84 ± 0.02	0.86 ± 0.0	0.8 ± 0.0	0.71 ± 0.0	0.72 ± 0.0	0.59 ± 0.0	0.83 ± 0.02	0.65 ± 0.0
0.95	500	0.8 ± 0.0	0.81 ± 0.01	0.87 ± 0.01	0.88 ± 0.0	0.79 ± 0.0	0.72 ± 0.0	0.75 ± 0.0	0.58 ± 0.0	0.87 ± 0.02	0.62 ± 0.0
0.95	550	0.75 ± 0.0	0.78 ± 0.01	0.84 ± 0.01	0.82 ± 0.0	0.76 ± 0.0	0.72 ± 0.0	0.69 ± 0.0	0.57 ± 0.0	0.81 ± 0.01	0.64 ± 0.0
0.95	600	0.73 ± 0.0	0.76 ± 0.01	0.79 ± 0.01	0.76 ± 0.0	0.73 ± 0.0	0.67 ± 0.0	0.67 ± 0.0	0.62 ± 0.0	0.78 ± 0.01	0.62 ± 0.0
0.95	650	0.74 ± 0.0	0.79 ± 0.01	0.81 ± 0.01	0.82 ± 0.0	0.75 ± 0.0	0.71 ± 0.0	0.72 ± 0.0	0.59 ± 0.0	0.8 ± 0.02	0.66 ± 0.0
0.95	700	0.73 ± 0.0	0.71 ± 0.01	0.81 ± 0.01	0.8 ± 0.0	0.77 ± 0.0	0.66 ± 0.0	0.66 ± 0.0	0.6 ± 0.0	0.79 ± 0.01	0.64 ± 0.0
						hCA XII					
-	150	0.84 ± 0.0	0.89 ± 0.03	0.83 ± 0.02	0.84 ± 0.01	0.75 ± 0.0	0.77 ± 0.0	0.81 ± 0.0	0.63 ± 0.0	0.83 ± 0.02	0.61 ± 0.0
-	200	0.81 ± 0.0	0.8 ± 0.01	0.87 ± 0.02	0.92 ± 0.0	0.78 ± 0.0	0.85 ± 0.0	0.8 ± 0.0	0.73 ± 0.0	0.85 ± 0.02	0.57 ± 0.0
-	250	0.82 ± 0.0	0.85 ± 0.02	0.91 ± 0.02	0.92 ± 0.0	0.76 ± 0.0	0.79 ± 0.0	0.84 ± 0.0	0.66 ± 0.0	0.88 ± 0.03	0.59 ± 0.0
-	300	0.74 ± 0.0	0.8 ± 0.01	0.84 ± 0.02	0.86 ± 0.01	0.72 ± 0.0	0.77 ± 0.0	0.82 ± 0.0	0.61 ± 0.0	0.82 ± 0.01	0.57 ± 0.0
-	350	0.73 ± 0.0	0.77 ± 0.01	0.83 ± 0.01	0.83 ± 0.0	0.75 ± 0.0	0.74 ± 0.0	0.77 ± 0.0	0.58 ± 0.0	0.8 ± 0.02	0.57 ± 0.0

PCC	N	AB	CART	ET	GBM	KNN	LDA	LR	NB	RF	SVM
-	400	0.73 ± 0.0	0.77 ± 0.02	0.85 ± 0.01	0.8 ± 0.0	0.72 ± 0.0	0.71 ± 0.0	0.75 ± 0.0	0.56 ± 0.0	0.83 ± 0.02	0.62 ± 0.0
-	450	0.76 ± 0.0	0.75 ± 0.01	0.83 ± 0.01	0.81 ± 0.0	0.74 ± 0.0	0.74 ± 0.0	0.75 ± 0.0	0.54 ± 0.0	0.81 ± 0.02	0.59 ± 0.0
-	500	0.74 ± 0.0	0.74 ± 0.01	0.81 ± 0.01	0.82 ± 0.0	0.76 ± 0.0	0.72 ± 0.0	0.71 ± 0.0	0.55 ± 0.0	0.8 ± 0.01	0.58 ± 0.0
-	550	0.76 ± 0.0	0.77 ± 0.01	0.82 ± 0.01	0.81 ± 0.01	0.74 ± 0.0	0.72 ± 0.0	0.71 ± 0.0	0.56 ± 0.0	0.79 ± 0.02	0.58 ± 0.0
-	600	0.69 ± 0.0	0.73 ± 0.01	0.77 ± 0.01	0.75 ± 0.0	0.7 ± 0.0	0.71 ± 0.0	0.7 ± 0.0	0.55 ± 0.0	0.76 ± 0.01	0.58 ± 0.0
-	650	0.74 ± 0.0	0.76 ± 0.01	0.8 ± 0.01	0.79 ± 0.0	0.73 ± 0.0	0.72 ± 0.0	0.73 ± 0.0	0.54 ± 0.0	0.78 ± 0.01	0.59 ± 0.0
-	700	0.72 ± 0.0	0.73 ± 0.01	0.78 ± 0.01	0.8 ± 0.0	0.69 ± 0.0	0.73 ± 0.0	0.73 ± 0.0	0.55 ± 0.0	0.75 ± 0.01	0.59 ± 0.0
0.75	150	0.84 ± 0.0	0.79 ± 0.03	0.85 ± 0.02	0.84 ± 0.01	0.81 ± 0.0	0.83 ± 0.0	0.83 ± 0.0	0.75 ± 0.0	0.82 ± 0.02	0.67 ± 0.0
0.75	200	0.82 ± 0.0	0.77 ± 0.01	0.88 ± 0.01	0.85 ± 0.0	0.85 ± 0.0	0.77 ± 0.0	0.8 ± 0.0	0.76 ± 0.0	0.86 ± 0.03	0.61 ± 0.0
0.75	250	0.87 ± 0.0	0.86 ± 0.02	0.92 ± 0.01	0.92 ± 0.01	0.8 ± 0.0	0.8 ± 0.0	0.82 ± 0.0	0.78 ± 0.0	0.9 ± 0.02	0.66 ± 0.0
0.75	300	0.77 ± 0.0	0.78 ± 0.02	0.86 ± 0.02	0.84 ± 0.01	0.75 ± 0.0	0.73 ± 0.0	0.73 ± 0.0	0.65 ± 0.0	0.85 ± 0.02	0.65 ± 0.0
0.75	350	0.76 ± 0.0	0.76 ± 0.01	0.84 ± 0.01	0.82 ± 0.0	0.78 ± 0.0	0.72 ± 0.0	0.73 ± 0.0	0.6 ± 0.0	0.82 ± 0.02	0.63 ± 0.0
0.75	400	0.72 ± 0.0	0.76 ± 0.01	0.83 ± 0.02	0.8 ± 0.0	0.74 ± 0.0	0.71 ± 0.0	0.74 ± 0.0	0.67 ± 0.0	0.82 ± 0.02	0.68 ± 0.0
0.75	450	0.76 ± 0.0	0.77 ± 0.01	0.83 ± 0.02	0.83 ± 0.0	0.78 ± 0.0	0.74 ± 0.0	0.73 ± 0.0	0.63 ± 0.0	0.82 ± 0.01	0.64 ± 0.0
0.75	500	0.72 ± 0.0	0.73 ± 0.01	0.81 ± 0.02	0.8 ± 0.0	0.73 ± 0.0	0.69 ± 0.0	0.71 ± 0.0	0.64 ± 0.0	0.78 ± 0.02	0.63 ± 0.0
0.75	550	0.74 ± 0.0	0.73 ± 0.01	0.81 ± 0.01	0.79 ± 0.0	0.73 ± 0.0	0.67 ± 0.0	0.71 ± 0.0	0.58 ± 0.0	0.8 ± 0.01	0.65 ± 0.0
0.75	600	0.69 ± 0.0	0.71 ± 0.01	0.78 ± 0.01	0.78 ± 0.0	0.69 ± 0.0	0.67 ± 0.0	0.68 ± 0.0	0.58 ± 0.0	0.77 ± 0.02	0.64 ± 0.0
0.75	650	0.69 ± 0.0	0.72 ± 0.01	0.8 ± 0.01	0.78 ± 0.0	0.72 ± 0.0	0.69 ± 0.0	0.68 ± 0.0	0.56 ± 0.0	0.77 ± 0.02	0.64 ± 0.0
0.75	700	0.74 ± 0.0	0.7 ± 0.01	0.77 ± 0.01	0.76 ± 0.0	0.72 ± 0.0	0.67 ± 0.0	0.69 ± 0.0	0.57 ± 0.0	0.77 ± 0.02	0.65 ± 0.0
0.95	150	0.85 ± 0.0	0.85 ± 0.02	0.85 ± 0.02	0.84 ± 0.0	0.81 ± 0.0	0.83 ± 0.0	0.84 ± 0.0	0.65 ± 0.0	0.81 ± 0.04	0.69 ± 0.0
0.95	200	0.85 ± 0.0	0.76 ± 0.01	0.87 ± 0.02	0.92 ± 0.0	0.84 ± 0.0	0.81 ± 0.0	0.8 ± 0.0	0.75 ± 0.0	0.86 ± 0.02	0.61 ± 0.0
0.95	250	0.83 ± 0.0	0.86 ± 0.02	0.91 ± 0.03	0.9 ± 0.0	0.82 ± 0.0	0.82 ± 0.0	0.81 ± 0.0	0.67 ± 0.0	0.9 ± 0.02	0.65 ± 0.0
0.95	300	0.78 ± 0.0	0.79 ± 0.01	0.85 ± 0.01	0.85 ± 0.01	0.78 ± 0.0	0.81 ± 0.0	0.76 ± 0.0	0.64 ± 0.0	0.84 ± 0.02	0.63 ± 0.0
0.95	350	0.71 ± 0.0	0.8 ± 0.01	0.83 ± 0.01	0.82 ± 0.0	0.77 ± 0.0	0.74 ± 0.0	0.74 ± 0.0	0.67 ± 0.0	0.83 ± 0.01	0.62 ± 0.0
0.95	400	0.77 ± 0.0	0.75 ± 0.02	0.85 ± 0.03	0.81 ± 0.0	0.78 ± 0.0	0.72 ± 0.0	0.74 ± 0.0	0.59 ± 0.0	0.83 ± 0.02	0.69 ± 0.0
0.95	450	0.79 ± 0.0	0.75 ± 0.01	0.83 ± 0.02	0.81 ± 0.0	0.8 ± 0.0	0.75 ± 0.0	0.76 ± 0.0	0.57 ± 0.0	0.8 ± 0.02	0.63 ± 0.0
0.95	500	0.78 ± 0.0	0.77 ± 0.01	0.82 ± 0.02	0.81 ± 0.0	0.75 ± 0.0	0.73 ± 0.0	0.7 ± 0.0	0.56 ± 0.0	0.8 ± 0.02	0.64 ± 0.0
0.95	550	0.8 ± 0.0	0.78 ± 0.01	0.82 ± 0.02	0.81 ± 0.0	0.78 ± 0.0	0.72 ± 0.0	0.7 ± 0.0	0.62 ± 0.0	0.81 ± 0.01	0.65 ± 0.0
0.95	600	0.7 ± 0.0	0.69 ± 0.01	0.77 ± 0.02	0.77 ± 0.0	0.74 ± 0.0	0.72 ± 0.0	0.72 ± 0.0	0.55 ± 0.0	0.77 ± 0.01	0.64 ± 0.0
0.95	650	0.74 ± 0.0	0.76 ± 0.01	0.8 ± 0.02	0.79 ± 0.0	0.74 ± 0.0	0.7 ± 0.0	0.71 ± 0.0	0.56 ± 0.0	0.79 ± 0.01	0.63 ± 0.0
0.95	700	0.72 ± 0.0	0.73 ± 0.01	0.78 ± 0.01	0.79 ± 0.0	0.74 ± 0.0	0.72 ± 0.0	0.72 ± 0.0	0.58 ± 0.0	0.76 ± 0.01	0.65 ± 0.0

Table S5. *Results of the testing phase on hCA isoforms II, IX and XII, expressed as averaged Matthews Correlation Coefficient (MCC) on 10 experiments (with standard deviations). Different Pearson Correlation Coefficient (PCC) values were used to generate the models (no PCC, PCC = 0.95, PCC = 0.75).*

PCC	N	AB	CART	ET	GBM	KNN	LDA	LR	NB	RF	SVM
						hCA II					
-	150	0.66 ± 0.0	0.77 ± 0.02	0.82 ± 0.06	0.72 ± 0.01	0.68 ± 0.0	0.74 ± 0.0	0.65 ± 0.0	0.47 ± 0.0	0.77 ± 0.04	0.4 ± 0.0
-	200	0.76 ± 0.0	0.61 ± 0.03	0.73 ± 0.04	0.8 ± 0.01	0.56 ± 0.0	0.7 ± 0.0	0.56 ± 0.0	0.17 ± 0.0	0.71 ± 0.07	0.45 ± 0.0
-	250	0.81 ± 0.0	0.66 ± 0.03	0.85 ± 0.03	0.84 ± 0.01	0.65 ± 0.0	0.57 ± 0.0	0.6 ± 0.0	0.32 ± 0.0	0.8 ± 0.05	0.45 ± 0.0
-	300	0.68 ± 0.0	0.76 ± 0.03	0.78 ± 0.03	0.8 ± 0.0	0.62 ± 0.0	0.67 ± 0.0	0.65 ± 0.0	0.35 ± 0.0	0.73 ± 0.02	0.31 ± 0.0
-	350	0.68 ± 0.0	0.74 ± 0.02	0.82 ± 0.02	0.78 ± 0.01	0.66 ± 0.0	0.63 ± 0.0	0.56 ± 0.0	0.4 ± 0.0	0.78 ± 0.04	0.39 ± 0.0
-	400	0.7 ± 0.0	0.68 ± 0.02	0.77 ± 0.02	0.73 ± 0.0	0.63 ± 0.0	0.61 ± 0.0	0.63 ± 0.0	0.34 ± 0.0	0.75 ± 0.02	0.43 ± 0.0
-	450	0.72 ± 0.0	0.56 ± 0.02	0.8 ± 0.03	0.76 ± 0.0	0.61 ± 0.0	0.59 ± 0.0	0.57 ± 0.0	0.4 ± 0.0	0.75 ± 0.03	0.36 ± 0.0
-	500	0.59 ± 0.0	0.66 ± 0.02	0.74 ± 0.03	0.73 ± 0.0	0.51 ± 0.0	0.6 ± 0.0	0.57 ± 0.0	0.3 ± 0.0	0.73 ± 0.02	0.33 ± 0.0
-	550	0.7 ± 0.0	0.72 ± 0.04	0.83 ± 0.02	0.74 ± 0.0	0.52 ± 0.0	0.67 ± 0.0	0.65 ± 0.0	0.39 ± 0.0	0.78 ± 0.04	0.37 ± 0.0
-	600	0.62 ± 0.0	0.72 ± 0.02	0.75 ± 0.03	0.78 ± 0.0	0.5 ± 0.0	0.64 ± 0.0	0.61 ± 0.0	0.29 ± 0.0	0.72 ± 0.02	0.29 ± 0.0
-	650	0.68 ± 0.0	0.61 ± 0.01	0.77 ± 0.03	0.74 ± 0.0	0.55 ± 0.0	0.67 ± 0.0	0.65 ± 0.0	0.26 ± 0.0	0.72 ± 0.03	0.23 ± 0.0
-	700	0.55 ± 0.0	0.67 ± 0.02	0.78 ± 0.02	0.75 ± 0.0	0.58 ± 0.0	0.65 ± 0.0	0.61 ± 0.0	0.25 ± 0.0	0.73 ± 0.03	0.33 ± 0.0
0.75	150	0.74 ± 0.0	0.76 ± 0.02	0.81 ± 0.05	0.87 ± 0.0	0.79 ± 0.0	0.42 ± 0.0	0.56 ± 0.0	0.44 ± 0.0	0.76 ± 0.08	0.46 ± 0.0
0.75	200	0.65 ± 0.0	0.63 ± 0.01	0.75 ± 0.02	0.72 ± 0.02	0.67 ± 0.0	0.38 ± 0.0	0.34 ± 0.0	0.26 ± 0.0	0.72 ± 0.05	0.48 ± 0.0
0.75	250	0.79 ± 0.0	0.69 ± 0.03	0.82 ± 0.04	0.84 ± 0.0	0.71 ± 0.0	0.51 ± 0.0	0.55 ± 0.0	0.44 ± 0.0	0.8 ± 0.03	0.46 ± 0.0
0.75	300	0.61 ± 0.0	0.62 ± 0.02	0.8 ± 0.03	0.7 ± 0.01	0.67 ± 0.0	0.52 ± 0.0	0.55 ± 0.0	0.48 ± 0.0	0.73 ± 0.03	0.33 ± 0.0
0.75	350	0.65 ± 0.0	0.64 ± 0.02	0.82 ± 0.02	0.78 ± 0.01	0.71 ± 0.0	0.46 ± 0.0	0.45 ± 0.0	0.47 ± 0.0	0.79 ± 0.04	0.44 ± 0.0
0.75	400	0.63 ± 0.0	0.69 ± 0.02	0.79 ± 0.02	0.79 ± 0.0	0.65 ± 0.0	0.5 ± 0.0	0.51 ± 0.0	0.37 ± 0.0	0.77 ± 0.04	0.47 ± 0.0
0.75	450	0.68 ± 0.0	0.68 ± 0.02	0.82 ± 0.02	0.79 ± 0.01	0.62 ± 0.0	0.4 ± 0.0	0.43 ± 0.0	0.48 ± 0.0	0.78 ± 0.03	0.42 ± 0.0
0.75	500	0.59 ± 0.0	0.65 ± 0.01	0.73 ± 0.04	0.75 ± 0.0	0.57 ± 0.0	0.34 ± 0.0	0.38 ± 0.0	0.43 ± 0.0	0.7 ± 0.04	0.42 ± 0.0
0.75	550	0.62 ± 0.0	0.67 ± 0.02	0.82 ± 0.01	0.76 ± 0.0	0.58 ± 0.0	0.53 ± 0.0	0.52 ± 0.0	0.46 ± 0.0	0.78 ± 0.02	0.5 ± 0.0
0.75	600	0.59 ± 0.0	0.65 ± 0.01	0.78 ± 0.02	0.73 ± 0.0	0.58 ± 0.0	0.43 ± 0.0	0.46 ± 0.0	0.37 ± 0.0	0.73 ± 0.01	0.42 ± 0.0
0.75	650	0.64 ± 0.0	0.62 ± 0.01	0.77 ± 0.02	0.74 ± 0.0	0.52 ± 0.0	0.52 ± 0.0	0.52 ± 0.0	0.39 ± 0.0	0.74 ± 0.03	0.37 ± 0.0
0.75	700	0.56 ± 0.0	0.63 ± 0.02	0.78 ± 0.03	0.69 ± 0.0	0.61 ± 0.0	0.43 ± 0.0	0.42 ± 0.0	0.35 ± 0.0	0.74 ± 0.02	0.49 ± 0.0
0.95	150	0.68 ± 0.0	0.71 ± 0.04	0.77 ± 0.05	0.82 ± 0.01	0.95 ± 0.0	0.77 ± 0.0	0.66 ± 0.0	0.36 ± 0.0	0.75 ± 0.04	0.55 ± 0.0
0.95	200	0.7 ± 0.0	0.65 ± 0.07	0.81 ± 0.03	0.78 ± 0.04	0.73 ± 0.0	0.52 ± 0.0	0.48 ± 0.0	0.21 ± 0.0	0.69 ± 0.05	0.48 ± 0.0
0.95	250	0.87 ± 0.0	0.68 ± 0.03	0.83 ± 0.04	0.79 ± 0.0	0.73 ± 0.0	0.6 ± 0.0	0.54 ± 0.0	0.36 ± 0.0	0.78 ± 0.04	0.48 ± 0.0
0.95	300	0.64 ± 0.0	0.73 ± 0.02	0.77 ± 0.03	0.77 ± 0.0	0.76 ± 0.0	0.61 ± 0.0	0.6 ± 0.0	0.35 ± 0.0	0.72 ± 0.04	0.33 ± 0.0
0.95	350	0.67 ± 0.0	0.74 ± 0.02	0.82 ± 0.01	0.8 ± 0.01	0.7 ± 0.0	0.57 ± 0.0	0.58 ± 0.0	0.42 ± 0.0	0.77 ± 0.03	0.45 ± 0.0
0.95	400	0.69 ± 0.0	0.68 ± 0.02	0.79 ± 0.01	0.74 ± 0.0	0.65 ± 0.0	0.56 ± 0.0	0.6 ± 0.0	0.33 ± 0.0	0.75 ± 0.04	0.5 ± 0.0
0.95	450	0.71 ± 0.0	0.57 ± 0.03	0.81 ± 0.03	0.79 ± 0.0	0.65 ± 0.0	0.59 ± 0.0	0.54 ± 0.0	0.47 ± 0.0	0.78 ± 0.02	0.42 ± 0.0
0.95	500	0.55 ± 0.0	0.65 ± 0.03	0.76 ± 0.03	0.73 ± 0.0	0.61 ± 0.0	0.48 ± 0.0	0.48 ± 0.0	0.37 ± 0.0	0.73 ± 0.04	0.44 ± 0.0
0.95	550	0.64 ± 0.0	0.73 ± 0.03	0.83 ± 0.02	0.79 ± 0.0	0.64 ± 0.0	0.6 ± 0.0	0.61 ± 0.0	0.46 ± 0.0	0.79 ± 0.02	0.49 ± 0.0
0.95	600	0.62 ± 0.0	0.71 ± 0.02	0.77 ± 0.02	0.81 ± 0.0	0.6 ± 0.0	0.54 ± 0.0	0.58 ± 0.0	0.3 ± 0.0	0.73 ± 0.03	0.42 ± 0.0
0.95	650	0.6 ± 0.0	0.67 ± 0.01	0.78 ± 0.02	0.75 ± 0.0	0.61 ± 0.0	0.64 ± 0.0	0.61 ± 0.0	0.31 ± 0.0	0.74 ± 0.03	0.38 ± 0.0
0.95	700	0.66 ± 0.0	0.67 ± 0.02	0.79 ± 0.03	0.76 ± 0.0	0.69 ± 0.0	0.56 ± 0.0	0.56 ± 0.0	0.33 ± 0.0	0.75 ± 0.03	0.48 ± 0.0
						hCA IX					

PCC	N	AB	CART	ET	GBM	KNN	LDA	LR	NB	RF	SVM
-	150	0.63 ± 0.0	0.8 ± 0.04	0.77 ± 0.05	0.78 ± 0.02	0.76 ± 0.0	0.63 ± 0.0	0.58 ± 0.0	-0.01 ± 0.0	0.7 ± 0.11	0.33 ± 0.0
-	200	0.54 ± 0.0	0.78 ± 0.03	0.72 ± 0.04	0.71 ± 0.01	0.57 ± 0.0	0.36 ± 0.0	0.44 ± 0.0	0.03 ± 0.0	0.69 ± 0.03	0.31 ± 0.0
-	250	0.64 ± 0.0	0.49 ± 0.02	0.66 ± 0.02	0.68 ± 0.0	0.54 ± 0.0	0.47 ± 0.0	0.49 ± 0.0	0.26 ± 0.0	0.61 ± 0.03	0.25 ± 0.0
-	300	0.63 ± 0.0	0.56 ± 0.04	0.74 ± 0.05	0.69 ± 0.01	0.56 ± 0.0	0.55 ± 0.0	0.56 ± 0.0	0.33 ± 0.0	0.66 ± 0.02	0.39 ± 0.0
-	350	0.6 ± 0.0	0.46 ± 0.03	0.62 ± 0.03	0.68 ± 0.0	0.46 ± 0.0	0.48 ± 0.0	0.41 ± 0.0	0.32 ± 0.0	0.58 ± 0.04	0.27 ± 0.0
-	400	0.56 ± 0.0	0.48 ± 0.02	0.64 ± 0.03	0.71 ± 0.01	0.56 ± 0.0	0.53 ± 0.0	0.58 ± 0.0	0.24 ± 0.0	0.6 ± 0.03	0.35 ± 0.0
-	450	0.58 ± 0.0	0.55 ± 0.03	0.67 ± 0.04	0.71 ± 0.02	0.55 ± 0.0	0.51 ± 0.0	0.49 ± 0.0	0.24 ± 0.0	0.64 ± 0.02	0.36 ± 0.0
-	500	0.62 ± 0.0	0.59 ± 0.02	0.73 ± 0.02	0.73 ± 0.01	0.54 ± 0.0	0.54 ± 0.0	0.51 ± 0.0	0.23 ± 0.0	0.68 ± 0.02	0.33 ± 0.0
-	550	0.54 ± 0.0	0.51 ± 0.02	0.62 ± 0.02	0.6 ± 0.0	0.39 ± 0.0	0.48 ± 0.0	0.46 ± 0.0	0.22 ± 0.0	0.57 ± 0.03	0.26 ± 0.0
-	600	0.47 ± 0.0	0.47 ± 0.02	0.56 ± 0.02	0.51 ± 0.0	0.35 ± 0.0	0.42 ± 0.0	0.41 ± 0.0	0.23 ± 0.0	0.52 ± 0.03	0.21 ± 0.0
-	650	0.48 ± 0.0	0.56 ± 0.02	0.62 ± 0.04	0.61 ± 0.01	0.38 ± 0.0	0.46 ± 0.0	0.46 ± 0.0	0.25 ± 0.0	0.56 ± 0.02	0.29 ± 0.0
-	700	0.41 ± 0.0	0.38 ± 0.01	0.61 ± 0.02	0.6 ± 0.0	0.37 ± 0.0	0.36 ± 0.0	0.37 ± 0.0	0.24 ± 0.0	0.56 ± 0.04	0.25 ± 0.0
0.75	150	0.58 ± 0.0	0.55 ± 0.05	0.78 ± 0.05	0.67 ± 0.03	0.55 ± 0.0	0.57 ± 0.0	0.49 ± 0.0	0.55 ± 0.0	0.69 ± 0.07	0.46 ± 0.0
0.75	200	0.62 ± 0.0	0.76 ± 0.02	0.75 ± 0.04	0.67 ± 0.01	0.61 ± 0.0	0.58 ± 0.0	0.52 ± 0.0	0.36 ± 0.0	0.69 ± 0.06	0.4 ± 0.0
0.75	250	0.54 ± 0.0	0.62 ± 0.03	0.66 ± 0.03	0.6 ± 0.02	0.62 ± 0.0	0.41 ± 0.0	0.38 ± 0.0	0.17 ± 0.0	0.64 ± 0.03	0.27 ± 0.0
0.75	300	0.57 ± 0.0	0.63 ± 0.01	0.74 ± 0.02	0.68 ± 0.01	0.65 ± 0.0	0.41 ± 0.0	0.53 ± 0.0	0.51 ± 0.0	0.69 ± 0.04	0.41 ± 0.0
0.75	350	0.51 ± 0.0	0.48 ± 0.02	0.62 ± 0.03	0.61 ± 0.01	0.49 ± 0.0	0.39 ± 0.0	0.39 ± 0.0	0.41 ± 0.0	0.61 ± 0.04	0.29 ± 0.0
0.75	400	0.5 ± 0.0	0.57 ± 0.02	0.66 ± 0.03	0.67 ± 0.0	0.56 ± 0.0	0.47 ± 0.0	0.46 ± 0.0	0.18 ± 0.0	0.65 ± 0.01	0.37 ± 0.0
0.75	450	0.49 ± 0.0	0.6 ± 0.02	0.66 ± 0.03	0.67 ± 0.01	0.68 ± 0.0	0.42 ± 0.0	0.42 ± 0.0	0.28 ± 0.0	0.68 ± 0.03	0.38 ± 0.0
0.75	500	0.51 ± 0.0	0.6 ± 0.02	0.73 ± 0.04	0.7 ± 0.01	0.62 ± 0.0	0.37 ± 0.0	0.36 ± 0.0	0.15 ± 0.0	0.73 ± 0.03	0.39 ± 0.0
0.75	550	0.54 ± 0.0	0.52 ± 0.02	0.67 ± 0.02	0.53 ± 0.0	0.51 ± 0.0	0.36 ± 0.0	0.33 ± 0.0	0.14 ± 0.0	0.64 ± 0.03	0.36 ± 0.0
0.75	600	0.36 ± 0.0	0.47 ± 0.02	0.58 ± 0.03	0.51 ± 0.01	0.49 ± 0.0	0.31 ± 0.0	0.33 ± 0.0	0.24 ± 0.0	0.55 ± 0.03	0.31 ± 0.0
0.75	650	0.46 ± 0.0	0.56 ± 0.02	0.62 ± 0.03	0.62 ± 0.0	0.53 ± 0.0	0.37 ± 0.0	0.33 ± 0.0	0.19 ± 0.0	0.61 ± 0.02	0.38 ± 0.0
0.75	700	0.37 ± 0.0	0.45 ± 0.01	0.63 ± 0.03	0.52 ± 0.0	0.46 ± 0.0	0.27 ± 0.0	0.25 ± 0.0	0.1 ± 0.0	0.57 ± 0.02	0.33 ± 0.0
0.95	150	0.56 ± 0.0	0.71 ± 0.07	0.78 ± 0.04	0.74 ± 0.02	0.73 ± 0.0	0.5 ± 0.0	0.52 ± 0.0	0.3 ± 0.0	0.77 ± 0.05	0.4 ± 0.0
0.95	200	0.5 ± 0.0	0.5 ± 0.06	0.76 ± 0.05	0.7 ± 0.03	0.74 ± 0.0	0.43 ± 0.0	0.44 ± 0.0	0.17 ± 0.0	0.71 ± 0.05	0.39 ± 0.0
0.95	250	0.59 ± 0.0	0.59 ± 0.03	0.68 ± 0.03	0.72 ± 0.01	0.66 ± 0.0	0.49 ± 0.0	0.43 ± 0.0	0.16 ± 0.0	0.64 ± 0.03	0.24 ± 0.0
0.95	300	0.65 ± 0.0	0.53 ± 0.02	0.74 ± 0.03	0.73 ± 0.01	0.69 ± 0.0	0.56 ± 0.0	0.55 ± 0.0	0.4 ± 0.0	0.7 ± 0.04	0.43 ± 0.0
0.95	350	0.59 ± 0.0	0.48 ± 0.02	0.65 ± 0.03	0.68 ± 0.01	0.54 ± 0.0	0.37 ± 0.0	0.4 ± 0.0	0.31 ± 0.0	0.66 ± 0.03	0.35 ± 0.0
0.95	400	0.61 ± 0.0	0.58 ± 0.02	0.66 ± 0.04	0.74 ± 0.01	0.57 ± 0.0	0.54 ± 0.0	0.56 ± 0.0	0.19 ± 0.0	0.64 ± 0.03	0.38 ± 0.0
0.95	450	0.51 ± 0.0	0.55 ± 0.03	0.69 ± 0.04	0.73 ± 0.0	0.6 ± 0.0	0.41 ± 0.0	0.43 ± 0.0	0.22 ± 0.0	0.67 ± 0.04	0.4 ± 0.0
0.95	500	0.61 ± 0.0	0.62 ± 0.02	0.75 ± 0.03	0.76 ± 0.0	0.58 ± 0.0	0.45 ± 0.0	0.5 ± 0.0	0.21 ± 0.0	0.75 ± 0.03	0.35 ± 0.0
0.95	550	0.5 ± 0.0	0.55 ± 0.02	0.69 ± 0.02	0.64 ± 0.0	0.52 ± 0.0	0.44 ± 0.0	0.38 ± 0.0	0.2 ± 0.0	0.62 ± 0.02	0.36 ± 0.0
0.95	600	0.46 ± 0.0	0.53 ± 0.03	0.58 ± 0.02	0.53 ± 0.0	0.45 ± 0.0	0.35 ± 0.0	0.33 ± 0.0	0.24 ± 0.0	0.56 ± 0.02	0.32 ± 0.0
0.95	650	0.48 ± 0.0	0.57 ± 0.02	0.63 ± 0.02	0.65 ± 0.0	0.5 ± 0.0	0.42 ± 0.0	0.44 ± 0.0	0.21 ± 0.0	0.6 ± 0.03	0.38 ± 0.0
0.95	700	0.45 ± 0.0	0.41 ± 0.02	0.62 ± 0.02	0.6 ± 0.0	0.53 ± 0.0	0.33 ± 0.0	0.32 ± 0.0	0.21 ± 0.0	0.58 ± 0.03	0.35 ± 0.0
hCA XII											
-	150	0.68 ± 0.0	0.78 ± 0.06	0.66 ± 0.04	0.7 ± 0.02	0.49 ± 0.0	0.56 ± 0.0	0.64 ± 0.0	0.27 ± 0.0	0.67 ± 0.05	0.35 ± 0.0
-	200	0.62 ± 0.0	0.6 ± 0.03	0.75 ± 0.03	0.84 ± 0.0	0.56 ± 0.0	0.7 ± 0.0	0.6 ± 0.0	0.49 ± 0.0	0.7 ± 0.04	0.27 ± 0.0
-	250	0.63 ± 0.0	0.7 ± 0.04	0.82 ± 0.04	0.84 ± 0.01	0.52 ± 0.0	0.58 ± 0.0	0.68 ± 0.0	0.32 ± 0.0	0.76 ± 0.05	0.31 ± 0.0
-	300	0.48 ± 0.0	0.6 ± 0.03	0.69 ± 0.04	0.72 ± 0.01	0.44 ± 0.0	0.55 ± 0.0	0.64 ± 0.0	0.21 ± 0.0	0.65 ± 0.02	0.25 ± 0.0
-	350	0.46 ± 0.0	0.53 ± 0.03	0.67 ± 0.03	0.66 ± 0.01	0.51 ± 0.0	0.47 ± 0.0	0.53 ± 0.0	0.21 ± 0.0	0.6 ± 0.05	0.24 ± 0.0

PCC	N	AB	CART	ET	GBM	KNN	LDA	LR	NB	RF	SVM
-	400	0.46 ± 0.0	0.54 ± 0.04	0.69 ± 0.03	0.61 ± 0.01	0.44 ± 0.0	0.42 ± 0.0	0.5 ± 0.0	0.2 ± 0.0	0.66 ± 0.05	0.35 ± 0.0
-	450	0.51 ± 0.0	0.49 ± 0.03	0.67 ± 0.03	0.62 ± 0.0	0.49 ± 0.0	0.48 ± 0.0	0.49 ± 0.0	0.11 ± 0.0	0.63 ± 0.04	0.28 ± 0.0
-	500	0.48 ± 0.0	0.49 ± 0.02	0.62 ± 0.03	0.64 ± 0.01	0.52 ± 0.0	0.45 ± 0.0	0.42 ± 0.0	0.16 ± 0.0	0.6 ± 0.03	0.27 ± 0.0
-	550	0.51 ± 0.0	0.53 ± 0.02	0.63 ± 0.01	0.62 ± 0.01	0.48 ± 0.0	0.44 ± 0.0	0.43 ± 0.0	0.16 ± 0.0	0.58 ± 0.04	0.26 ± 0.0
-	600	0.39 ± 0.0	0.46 ± 0.03	0.55 ± 0.03	0.51 ± 0.01	0.41 ± 0.0	0.42 ± 0.0	0.41 ± 0.0	0.14 ± 0.0	0.52 ± 0.03	0.27 ± 0.0
-	650	0.48 ± 0.0	0.53 ± 0.01	0.61 ± 0.02	0.58 ± 0.0	0.45 ± 0.0	0.44 ± 0.0	0.45 ± 0.0	0.09 ± 0.0	0.56 ± 0.03	0.28 ± 0.0
-	700	0.43 ± 0.0	0.45 ± 0.02	0.57 ± 0.02	0.6 ± 0.0	0.37 ± 0.0	0.45 ± 0.0	0.46 ± 0.0	0.13 ± 0.0	0.51 ± 0.03	0.3 ± 0.0
0.75	150	0.7 ± 0.0	0.59 ± 0.06	0.71 ± 0.04	0.68 ± 0.01	0.64 ± 0.0	0.65 ± 0.0	0.66 ± 0.0	0.5 ± 0.0	0.64 ± 0.05	0.42 ± 0.0
0.75	200	0.64 ± 0.0	0.55 ± 0.03	0.77 ± 0.03	0.7 ± 0.01	0.7 ± 0.0	0.54 ± 0.0	0.6 ± 0.0	0.54 ± 0.0	0.72 ± 0.05	0.35 ± 0.0
0.75	250	0.74 ± 0.0	0.72 ± 0.04	0.83 ± 0.03	0.85 ± 0.01	0.6 ± 0.0	0.6 ± 0.0	0.63 ± 0.0	0.55 ± 0.0	0.79 ± 0.03	0.44 ± 0.0
0.75	300	0.53 ± 0.0	0.55 ± 0.04	0.73 ± 0.04	0.69 ± 0.01	0.49 ± 0.0	0.45 ± 0.0	0.47 ± 0.0	0.4 ± 0.0	0.7 ± 0.04	0.4 ± 0.0
0.75	350	0.52 ± 0.0	0.52 ± 0.03	0.68 ± 0.02	0.65 ± 0.0	0.57 ± 0.0	0.44 ± 0.0	0.46 ± 0.0	0.3 ± 0.0	0.65 ± 0.05	0.35 ± 0.0
0.75	400	0.44 ± 0.0	0.52 ± 0.02	0.67 ± 0.04	0.59 ± 0.0	0.48 ± 0.0	0.41 ± 0.0	0.48 ± 0.0	0.36 ± 0.0	0.65 ± 0.03	0.43 ± 0.0
0.75	450	0.52 ± 0.0	0.55 ± 0.02	0.66 ± 0.04	0.66 ± 0.0	0.57 ± 0.0	0.48 ± 0.0	0.46 ± 0.0	0.3 ± 0.0	0.63 ± 0.02	0.36 ± 0.0
0.75	500	0.43 ± 0.0	0.45 ± 0.02	0.62 ± 0.03	0.6 ± 0.0	0.46 ± 0.0	0.38 ± 0.0	0.42 ± 0.0	0.31 ± 0.0	0.57 ± 0.03	0.33 ± 0.0
0.75	550	0.48 ± 0.0	0.47 ± 0.02	0.62 ± 0.03	0.59 ± 0.0	0.47 ± 0.0	0.35 ± 0.0	0.43 ± 0.0	0.22 ± 0.0	0.6 ± 0.03	0.38 ± 0.0
0.75	600	0.38 ± 0.0	0.42 ± 0.02	0.56 ± 0.02	0.55 ± 0.0	0.37 ± 0.0	0.35 ± 0.0	0.37 ± 0.0	0.21 ± 0.0	0.54 ± 0.05	0.38 ± 0.0
0.75	650	0.38 ± 0.0	0.45 ± 0.02	0.6 ± 0.02	0.56 ± 0.0	0.45 ± 0.0	0.39 ± 0.0	0.35 ± 0.0	0.17 ± 0.0	0.54 ± 0.04	0.34 ± 0.0
0.75	700	0.47 ± 0.0	0.39 ± 0.03	0.54 ± 0.03	0.53 ± 0.01	0.45 ± 0.0	0.35 ± 0.0	0.37 ± 0.0	0.23 ± 0.0	0.54 ± 0.04	0.39 ± 0.0
0.95	150	0.71 ± 0.0	0.71 ± 0.04	0.7 ± 0.05	0.69 ± 0.01	0.63 ± 0.0	0.65 ± 0.0	0.69 ± 0.0	0.33 ± 0.0	0.61 ± 0.08	0.46 ± 0.0
0.95	200	0.7 ± 0.0	0.52 ± 0.03	0.74 ± 0.04	0.84 ± 0.0	0.68 ± 0.0	0.62 ± 0.0	0.6 ± 0.0	0.54 ± 0.0	0.73 ± 0.04	0.35 ± 0.0
0.95	250	0.66 ± 0.0	0.72 ± 0.04	0.82 ± 0.06	0.8 ± 0.01	0.65 ± 0.0	0.65 ± 0.0	0.62 ± 0.0	0.36 ± 0.0	0.8 ± 0.04	0.41 ± 0.0
0.95	300	0.56 ± 0.0	0.59 ± 0.03	0.71 ± 0.03	0.71 ± 0.01	0.56 ± 0.0	0.61 ± 0.0	0.52 ± 0.0	0.29 ± 0.0	0.68 ± 0.04	0.38 ± 0.0
0.95	350	0.42 ± 0.0	0.59 ± 0.02	0.67 ± 0.03	0.64 ± 0.01	0.54 ± 0.0	0.49 ± 0.0	0.49 ± 0.0	0.35 ± 0.0	0.66 ± 0.02	0.32 ± 0.0
0.95	400	0.54 ± 0.0	0.51 ± 0.04	0.69 ± 0.05	0.62 ± 0.01	0.56 ± 0.0	0.43 ± 0.0	0.47 ± 0.0	0.26 ± 0.0	0.66 ± 0.04	0.46 ± 0.0
0.95	450	0.58 ± 0.0	0.5 ± 0.03	0.67 ± 0.04	0.63 ± 0.01	0.59 ± 0.0	0.49 ± 0.0	0.51 ± 0.0	0.2 ± 0.0	0.61 ± 0.04	0.34 ± 0.0
0.95	500	0.55 ± 0.0	0.54 ± 0.03	0.63 ± 0.04	0.62 ± 0.01	0.51 ± 0.0	0.46 ± 0.0	0.4 ± 0.0	0.17 ± 0.0	0.6 ± 0.03	0.35 ± 0.0
0.95	550	0.59 ± 0.0	0.56 ± 0.02	0.64 ± 0.04	0.62 ± 0.01	0.56 ± 0.0	0.43 ± 0.0	0.4 ± 0.0	0.24 ± 0.0	0.61 ± 0.02	0.37 ± 0.0
0.95	600	0.4 ± 0.0	0.39 ± 0.01	0.54 ± 0.03	0.55 ± 0.0	0.47 ± 0.0	0.44 ± 0.0	0.43 ± 0.0	0.12 ± 0.0	0.53 ± 0.03	0.36 ± 0.0
0.95	650	0.48 ± 0.0	0.52 ± 0.02	0.61 ± 0.04	0.59 ± 0.0	0.47 ± 0.0	0.39 ± 0.0	0.42 ± 0.0	0.14 ± 0.0	0.58 ± 0.02	0.34 ± 0.0
0.95	700	0.44 ± 0.0	0.47 ± 0.02	0.57 ± 0.03	0.58 ± 0.0	0.49 ± 0.0	0.45 ± 0.0	0.44 ± 0.0	0.18 ± 0.0	0.53 ± 0.03	0.39 ± 0.0

Table S6. *Results of the training phase on hCA isoforms II, IX and XII, obtained by using a fixed threshold sampling approach. The results are expressed as averaged accuracy and Matthews Correlation Coefficient (MCC) values. Active class contains molecules with activity below 20 nM, inactive class contains molecules with activity above 100 nM.*

**Isoform**	**PCC**	**AB**	**CART**	**ET**	**GBM**	**KNN**	**LDA**	**LR**	**NB**	**RF**	**SVM**
						Accuracy					
II	-	0.72 ± 0.05	0.71 ± 0.05	0.78 ± 0.05	0.78 ± 0.03	0.68 ± 0.05	0.71 ± 0.05	0.71 ± 0.05	0.5 ± 0.06	0.76 ± 0.05	0.61 ± 0.02
II	0.95	0.7 ± 0.03	0.71 ± 0.03	0.78 ± 0.05	0.77 ± 0.04	0.72 ± 0.02	0.71 ± 0.06	0.7 ± 0.04	0.46 ± 0.04	0.76 ± 0.06	0.63 ± 0.02
II	0.75	0.71 ± 0.05	0.73 ± 0.03	0.77 ± 0.03	0.76 ± 0.04	0.71 ± 0.05	0.64 ± 0.04	0.64 ± 0.03	0.44 ± 0.02	0.75 ± 0.02	0.61 ± 0.02
IX	-	0.76 ± 0.04	0.77 ± 0.04	0.8 ± 0.04	0.8 ± 0.06	0.69 ± 0.05	0.71 ± 0.03	0.71 ± 0.04	0.62 ± 0.04	0.77 ± 0.03	0.66 ± 0.01
IX	0.95	0.76 ± 0.04	0.78 ± 0.03	0.82 ± 0.04	0.81 ± 0.06	0.74 ± 0.03	0.7 ± 0.03	0.7 ± 0.05	0.62 ± 0.04	0.79 ± 0.04	0.66 ± 0.01
IX	0.75	0.72 ± 0.03	0.77 ± 0.02	0.82 ± 0.05	0.78 ± 0.04	0.71 ± 0.03	0.7 ± 0.05	0.69 ± 0.05	0.62 ± 0.04	0.8 ± 0.03	0.67 ± 0.02
XII	-	0.88 ± 0.03	0.84 ± 0.02	0.9 ± 0.02	0.89 ± 0.02	0.86 ± 0.03	0.85 ± 0.04	0.85 ± 0.03	0.36 ± 0.05	0.9 ± 0.02	0.84 ± 0.02
XII	0.95	0.88 ± 0.03	0.85 ± 0.03	0.91 ± 0.03	0.89 ± 0.02	0.87 ± 0.02	0.85 ± 0.05	0.84 ± 0.04	0.35 ± 0.05	0.9 ± 0.03	0.83 ± 0.02
XII	0.75	0.88 ± 0.02	0.85 ± 0.04	0.91 ± 0.03	0.89 ± 0.02	0.85 ± 0.02	0.82 ± 0.05	0.83 ± 0.03	0.34 ± 0.04	0.89 ± 0.02	0.83 ± 0.02
						MCC					
II	-	0.42 ± 0.11	0.4 ± 0.11	0.54 ± 0.1	0.55 ± 0.07	0.34 ± 0.1	0.4 ± 0.11	0.41 ± 0.1	0.07 ± 0.13	0.51 ± 0.1	0.2 ± 0.08
II	0.95	0.38 ± 0.07	0.41 ± 0.07	0.55 ± 0.1	0.53 ± 0.08	0.42 ± 0.04	0.4 ± 0.13	0.38 ± 0.08	0.03 ± 0.12	0.5 ± 0.13	0.24 ± 0.07
II	0.75	0.41 ± 0.11	0.46 ± 0.07	0.53 ± 0.07	0.51 ± 0.08	0.39 ± 0.1	0.25 ± 0.1	0.24 ± 0.07	0.02 ± 0.1	0.49 ± 0.05	0.19 ± 0.08
IX	-	0.48 ± 0.09	0.52 ± 0.09	0.57 ± 0.09	0.58 ± 0.12	0.33 ± 0.12	0.38 ± 0.07	0.37 ± 0.09	0.12 ± 0.12	0.51 ± 0.06	0.27 ± 0.04
IX	0.95	0.5 ± 0.09	0.55 ± 0.06	0.62 ± 0.08	0.6 ± 0.12	0.46 ± 0.07	0.36 ± 0.06	0.36 ± 0.1	0.13 ± 0.1	0.56 ± 0.09	0.27 ± 0.05
IX	0.75	0.41 ± 0.07	0.52 ± 0.04	0.61 ± 0.11	0.54 ± 0.08	0.38 ± 0.06	0.34 ± 0.12	0.32 ± 0.11	0.14 ± 0.1	0.59 ± 0.06	0.29 ± 0.06
XII	-	0.55 ± 0.12	0.5 ± 0.08	0.64 ± 0.09	0.6 ± 0.08	0.48 ± 0.11	0.45 ± 0.16	0.44 ± 0.13	0.11 ± 0.08	0.64 ± 0.09	0.29 ± 0.14
XII	0.95	0.54 ± 0.13	0.5 ± 0.09	0.68 ± 0.11	0.58 ± 0.1	0.52 ± 0.06	0.43 ± 0.19	0.38 ± 0.16	0.11 ± 0.1	0.65 ± 0.12	0.26 ± 0.16
XII	0.75	0.55 ± 0.09	0.52 ± 0.15	0.66 ± 0.12	0.6 ± 0.07	0.46 ± 0.09	0.29 ± 0.21	0.32 ± 0.17	0.14 ± 0.05	0.61 ± 0.08	0.26 ± 0.16

Table S7. *Results of the testing phase on hCA isoforms II, IX and XII, expressed as averaged accuracy, Matthews Correlation Coefficient (MCC) on 10 experiments (with standard deviations). Active class contains molecules with activity below 20 nM, inactive class contains molecules with activity above 100 nM.*

**Isoform**	**PCC**	**AB**	**CART**	**ET**	**GBM**	**KNN**	**LDA**	**LR**	**NB**	**RF**	**SVM**
						Accuracy					
II	-	0.69 ± 0.08	0.73 ± 0.01	0.73 ± 0.09	0.7 ± 0.09	0.73 ± 0.01	0.74 ± 0.01	0.74 ± 0.0	0.69 ± 0.09	0.71 ± 0.09	0.68 ± 0.09
II	0.75	0.66 ± 0.1	0.68 ± 0.07	0.71 ± 0.12	0.68 ± 0.11	0.65 ± 0.06	0.61 ± 0.01	0.61 ± 0.0	0.64 ± 0.11	0.69 ± 0.11	0.64 ± 0.1
II	0.95	0.69 ± 0.1	0.74 ± 0.04	0.73 ± 0.1	0.7 ± 0.1	0.73 ± 0.04	0.7 ± 0.01	0.71 ± 0.0	0.68 ± 0.11	0.71 ± 0.1	0.68 ± 0.11
IX	-	0.7 ± 0.06	0.72 ± 0.03	0.72 ± 0.07	0.71 ± 0.07	0.71 ± 0.02	0.69 ± 0.01	0.68 ± 0.0	0.69 ± 0.06	0.71 ± 0.07	0.68 ± 0.06
IX	0.75	0.69 ± 0.05	0.71 ± 0.05	0.73 ± 0.07	0.7 ± 0.06	0.7 ± 0.05	0.66 ± 0.01	0.66 ± 0.0	0.69 ± 0.06	0.72 ± 0.07	0.69 ± 0.05
IX	0.95	0.7 ± 0.07	0.71 ± 0.06	0.73 ± 0.07	0.71 ± 0.07	0.7 ± 0.06	0.66 ± 0.01	0.66 ± 0.0	0.69 ± 0.07	0.72 ± 0.07	0.68 ± 0.06
XII	-	0.77 ± 0.18	0.85 ± 0.02	0.81 ± 0.16	0.79 ± 0.17	0.86 ± 0.0	0.86 ± 0.0	0.87 ± 0.0	0.75 ± 0.21	0.8 ± 0.16	0.76 ± 0.19
XII	0.75	0.78 ± 0.19	0.85 ± 0.02	0.81 ± 0.17	0.79 ± 0.18	0.86 ± 0.01	0.86 ± 0.0	0.85 ± 0.0	0.75 ± 0.21	0.8 ± 0.17	0.76 ± 0.2
XII	0.95	0.79 ± 0.17	0.86 ± 0.01	0.82 ± 0.15	0.8 ± 0.17	0.87 ± 0.01	0.87 ± 0.01	0.86 ± 0.0	0.76 ± 0.2	0.81 ± 0.16	0.77 ± 0.19
						MCC					
II	-	0.36 ± 0.14	0.45 ± 0.03	0.44 ± 0.17	0.39 ± 0.15	0.44 ± 0.02	0.46 ± 0.01	0.47 ± 0.0	0.38 ± 0.14	0.42 ± 0.16	0.35 ± 0.14
II	0.75	0.32 ± 0.16	0.33 ± 0.16	0.42 ± 0.2	0.36 ± 0.19	0.27 ± 0.13	0.17 ± 0.01	0.19 ± 0.0	0.29 ± 0.17	0.39 ± 0.2	0.29 ± 0.16
II	0.95	0.38 ± 0.15	0.46 ± 0.08	0.45 ± 0.17	0.41 ± 0.16	0.44 ± 0.08	0.38 ± 0.02	0.4 ± 0.0	0.38 ± 0.17	0.43 ± 0.17	0.36 ± 0.16
IX	-	0.33 ± 0.16	0.39 ± 0.07	0.39 ± 0.16	0.36 ± 0.16	0.36 ± 0.05	0.33 ± 0.02	0.31 ± 0.0	0.32 ± 0.16	0.38 ± 0.16	0.3 ± 0.15
IX	0.75	0.32 ± 0.13	0.37 ± 0.11	0.4 ± 0.16	0.35 ± 0.14	0.34 ± 0.11	0.26 ± 0.02	0.24 ± 0.0	0.32 ± 0.15	0.38 ± 0.16	0.31 ± 0.14
IX	0.95	0.34 ± 0.17	0.38 ± 0.13	0.41 ± 0.18	0.37 ± 0.17	0.34 ± 0.12	0.25 ± 0.01	0.27 ± 0.0	0.32 ± 0.17	0.39 ± 0.18	0.3 ± 0.16
XII	-	0.39 ± 0.15	0.48 ± 0.04	0.45 ± 0.16	0.41 ± 0.15	0.51 ± 0.02	0.52 ± 0.0	0.52 ± 0.0	0.4 ± 0.17	0.43 ± 0.16	0.38 ± 0.16
XII	0.75	0.4 ± 0.15	0.48 ± 0.04	0.46 ± 0.16	0.42 ± 0.15	0.49 ± 0.04	0.46 ± 0.01	0.44 ± 0.0	0.4 ± 0.17	0.44 ± 0.16	0.38 ± 0.16
XII	0.95	0.42 ± 0.15	0.5 ± 0.05	0.47 ± 0.16	0.43 ± 0.15	0.52 ± 0.04	0.52 ± 0.05	0.46 ± 0.0	0.42 ± 0.17	0.45 ± 0.15	0.4 ± 0.16

Table S8. *Comparison of testing phase results using the "fixed threshold method", which refers to the traditional method of sampling the active vs inactive class by choosing fixed bioactivity thresholds (in this case, activity thresholds to define "active" and "inactive" compounds were 20 and 100 nM, 10 and 100 nM, 20 and 200 nM and 50 and 250 nM). The Extra Tree algorithm was used for all calculations.*

Metrics	Isoform	PCC	Threshold	Accuracy	MCC	Precision	Recall
Accuracy	II	0.75	10-100nM	0.7 ± 0.09	0.42 ± 0.17	0.72 ± 0.16	0.71 ± 0.08
Accuracy	II	0.75	20-200nM	0.69 ± 0.08	0.37 ± 0.16	0.78 ± 0.13	0.72 ± 0.07
Accuracy	II	0.75	50-250nM	0.7 ± 0.08	0.33 ± 0.17	0.81 ± 0.14	0.74 ± 0.06
Accuracy	II	0.75	20-100nM	0.71 ± 0.12	0.42 ± 0.2	0.78 ± 0.24	0.74 ± 0.07
Accuracy	II	0.95	10-100nM	0.73 ± 0.07	0.46 ± 0.14	0.8 ± 0.07	0.72 ± 0.08
Accuracy	II	0.95	20-200nM	0.71 ± 0.06	0.4 ± 0.13	0.83 ± 0.07	0.73 ± 0.07
Accuracy	II	0.95	50-250nM	0.72 ± 0.05	0.37 ± 0.14	0.86 ± 0.06	0.75 ± 0.06
Accuracy	II	0.95	20-100nM	0.73 ± 0.1	0.45 ± 0.17	0.79 ± 0.22	0.75 ± 0.07
Accuracy	II	-	10-100nM	0.72 ± 0.08	0.45 ± 0.15	0.8 ± 0.08	0.72 ± 0.09
Accuracy	II	-	20-100nM	0.72 ± 0.09	0.44 ± 0.16	0.81 ± 0.17	0.74 ± 0.06
Accuracy	II	-	20-200nM	0.71 ± 0.07	0.39 ± 0.16	0.84 ± 0.06	0.72 ± 0.08
Accuracy	II	-	50-250nM	0.72 ± 0.06	0.37 ± 0.16	0.86 ± 0.05	0.75 ± 0.06
Accuracy	IX	0.75	10-100nM	0.7 ± 0.07	0.39 ± 0.15	0.84 ± 0.06	0.69 ± 0.07
Accuracy	IX	0.75	20-200nM	0.72 ± 0.06	0.39 ± 0.15	0.86 ± 0.05	0.74 ± 0.07
Accuracy	IX	0.75	50-250nM	0.75 ± 0.05	0.37 ± 0.16	0.89 ± 0.05	0.78 ± 0.06
Accuracy	IX	0.75	20-100nM	0.73 ± 0.07	0.4 ± 0.17	0.87 ± 0.06	0.74 ± 0.07
Accuracy	IX	0.95	10-100nM	0.71 ± 0.07	0.41 ± 0.13	0.84 ± 0.06	0.7 ± 0.06
Accuracy	IX	0.95	20-200nM	0.72 ± 0.08	0.42 ± 0.15	0.82 ± 0.12	0.76 ± 0.06
Accuracy	IX	0.95	50-250nM	0.74 ± 0.06	0.37 ± 0.14	0.87 ± 0.07	0.78 ± 0.05
Accuracy	IX	0.95	20-100nM	0.73 ± 0.07	0.41 ± 0.18	0.86 ± 0.05	0.75 ± 0.07
Accuracy	IX	-	10-100nM	0.7 ± 0.06	0.39 ± 0.13	0.81 ± 0.08	0.7 ± 0.06
Accuracy	IX	-	20-100nM	0.72 ± 0.07	0.4 ± 0.17	0.86 ± 0.05	0.74 ± 0.06
Accuracy	IX	-	20-200nM	0.72 ± 0.08	0.41 ± 0.14	0.8 ± 0.15	0.76 ± 0.05
Accuracy	IX	-	50-250nM	0.74 ± 0.05	0.37 ± 0.11	0.86 ± 0.1	0.78 ± 0.04
Accuracy	XII	0.75	10-100nM	0.81 ± 0.14	0.47 ± 0.17	0.88 ± 0.21	0.87 ± 0.03
Accuracy	XII	0.75	20-200nM	0.84 ± 0.09	0.5 ± 0.14	0.9 ± 0.13	0.9 ± 0.03
Accuracy	XII	0.75	50-250nM	0.86 ± 0.08	0.48 ± 0.14	0.93 ± 0.11	0.91 ± 0.02
Accuracy	XII	0.75	20-100nM	0.81 ± 0.17	0.46 ± 0.16	0.88 ± 0.23	0.89 ± 0.02
Accuracy	XII	0.95	10-100nM	0.81 ± 0.14	0.5 ± 0.17	0.88 ± 0.2	0.88 ± 0.03
Accuracy	XII	0.95	20-200nM	0.84 ± 0.15	0.56 ± 0.17	0.88 ± 0.2	0.92 ± 0.03
Accuracy	XII	0.95	50-250nM	0.85 ± 0.16	0.51 ± 0.17	0.9 ± 0.2	0.92 ± 0.02
Accuracy	XII	0.95	20-100nM	0.82 ± 0.15	0.47 ± 0.16	0.88 ± 0.21	0.89 ± 0.02
Accuracy	XII	-	10-100nM	0.82 ± 0.14	0.5 ± 0.17	0.89 ± 0.21	0.88 ± 0.03
Accuracy	XII	-	20-200nM	0.83 ± 0.16	0.54 ± 0.17	0.88 ± 0.21	0.91 ± 0.03
Accuracy	XII	-	50-250nM	0.83 ± 0.17	0.49 ± 0.16	0.89 ± 0.22	0.91 ± 0.02
Accuracy	XII	-	20-100nM	0.81 ± 0.16	0.45 ± 0.16	0.88 ± 0.22	0.89 ± 0.02

Table S9. *Standard Deviation values for the probability scores in the validation phase. The reported values refer to models built using the Extra Tree algorithm.*

N	Class	Probability score Standard Deviations (Inactive class)	Probability score Standard Deviations (Active class)
150	II	0.11	0.11
200	II	0.11	0.11
250	II	0.11	0.11
300	II	0.11	0.11
350	II	0.11	0.11
400	II	0.10	0.10
450	II	0.10	0.10
500	II	0.10	0.11
550	II	0.10	0.10
600	II	0.09	0.10
150	IX	0.10	0.10
200	IX	0.10	0.10
250	IX	0.10	0.10
300	IX	0.10	0.10
350	IX	0.10	0.10
400	IX	0.10	0.10
450	IX	0.10	0.10
500	IX	0.09	0.10
550	IX	0.09	0.10
600	IX	0.09	0.10
150	XII	0.11	0.10
200	XII	0.10	0.10
250	XII	0.10	0.10
300	XII	0.10	0.10
350	XII	0.10	0.10
400	XII	0.10	0.10
450	XII	0.10	0.10
500	XII	0.09	0.10
550	XII	0.10	0.10
600	XII	0.09	0.10

Table S10. *Results of the validation phase on two subsets extracted from the validation dataset, based on the 2D similarity calculated with Atom Pair fingerprints (*APfp*) against training dataset molecules. A mean Tc_{APfp} threshold of 0.336 was set to separate validation molecules in either the "not similar" subset or the "similar" subset.*

Prob (mean)	*N*	hCA isoform	TP	FN	FP	TN	Accuracy	MCC	Precision	Recall	Validation subset
**0.6**	350	II	142	261	52	175	0.50	0.13	0.73	0.35	*not similar*
**0.7**	350	II	210	197	92	174	0.57	0.17	0.70	0.52	*not similar*
**0.8**	350	II	126	153	17	86	0.55	0.26	0.88	0.45	*not similar*
**0.9**	350	II	135	43	5	120	0.84	0.71	0.96	0.76	*not similar*
**1**	350	II	112	17	6	58	0.88	0.75	0.95	0.87	*not similar*
**Overall**	350	II	725	671	172	613	0.61	0.29	0.81	0.52	*not similar*
**0.6**	350	II	9	18	18	10	0.35	-0.31	0.33	0.33	*similar*
**0.7**	350	II	13	31	11	18	0.42	-0.09	0.54	0.30	*similar*
**0.8**	350	II	5	14	0	24	0.67	0.41	1.00	0.26	*similar*
**0.9**	350	II	15	5	0	5	0.80	0.61	1.00	0.75	*similar*
**1**	350	II	15	1	3	12	0.87	0.75	0.83	0.94	*similar*
**Overall**	350	II	57	70	32	70	0.55	0.14	0.64	0.45	*similar*
**0.6**	450	IX	21	24	4	18	0.58	0.28	0.84	0.47	*not similar*
**0.7**	450	IX	50	12	12	26	0.76	0.49	0.81	0.81	*not similar*
**0.8**	450	IX	31	10	6	14	0.74	0.44	0.84	0.76	*not similar*
**0.9**	450	IX	25	5	6	20	0.80	0.60	0.81	0.83	*not similar*
**1**	450	IX	26	1	1	12	0.95	0.89	0.96	0.96	*not similar*
**Overall**	450	IX	153	52	29	91	0.75	0.49	0.84	0.75	*not similar*
**0.6**	450	IX	25	22	11	20	0.58	0.17	0.69	0.53	*similar*
**0.7**	450	IX	53	17	8	32	0.77	0.54	0.87	0.76	*similar*
**0.8**	450	IX	44	5	5	18	0.86	0.68	0.90	0.90	*similar*
**0.9**	450	IX	34	8	2	26	0.86	0.72	0.94	0.81	*similar*
**1**	450	IX	17	5	6	25	0.79	0.58	0.74	0.77	*similar*
**Overall**	450	IX	173	58	32	121	0.77	0.53	0.84	0.75	*similar*
**0.6**	150	XII	56	59	6	10	0.50	0.07	0.90	0.49	*not similar*
**0.7**	150	XII	104	80	6	7	0.56	0.05	0.95	0.57	*not similar*
**0.8**	150	XII	68	23	3	1	0.73	0.00	0.96	0.75	*not similar*
**0.9**	150	XII	20	16	1	7	0.61	0.33	0.95	0.56	*not similar*
**1**	150	XII	19	3	0	6	0.89	0.76	1.00	0.86	*not similar*
**Overall**	150	XII	267	181	16	31	0.60	0.15	0.94	0.60	*not similar*
**0.6**	150	XII	32	35	1	10	0.54	0.27	0.97	0.48	*similar*
**0.7**	150	XII	79	36	7	5	0.66	0.06	0.92	0.69	*similar*
**0.8**	150	XII	66	12	4	3	0.81	0.20	0.94	0.85	*similar*
**0.9**	150	XII	35	4	3	12	0.87	0.68	0.92	0.90	*similar*
**1**	150	XII	45	3	1	2	0.92	0.48	0.98	0.94	*similar*
**Overall**	150	XII	257	90	16	32	0.73	0.29	0.94	0.74	*similar*

Table S11. *List of RDKit molecular descriptors used in the machine learning experiments. Descriptors filtered by applying a Pearson Correlation Coefficient (PCC) of 0.95 and 0.75 are marked with an "X".*

**Molecular Descriptors**	**Filtered at PCC = 0.95?**	**Filtered at PCC = 0.75?**
AMW	X	X
Chi0v	X	X
Chi1n	X	X
Chi1v	X	X
Chi2n	X	X
Chi2v	X	X
Chi3n	X	X
Chi3v	X	X
Chi4n	X	X
Chi4v	X	X
ExactMW	X	X
FractionCSP3		
HallKierAlpha		
kappa1	X	X
kappa2		X
kappa3		
LabuteASA	X	X
MQN1	X	X
MQN10		X
MQN11		
MQN12	X	X
MQN13		X
MQN14		
MQN15		
MQN16		X
MQN17	X	X
MQN18		
MQN19	X	X
MQN2		X
MQN20	X	X
MQN21	X	X
MQN22	X	X
MQN23	X	X
MQN24		
MQN25	X	X
MQN26		X
MQN27		X
MQN28		
MQN29		X
MQN3		X
MQN30		X
MQN31		X
MQN32		X
MQN33		
MQN34		
MQN35		
MQN36		X
MQN37		
MQN38		
MQN39		
MQN4		
MQN40		
MQN41		
MQN42		X
MQN5		
MQN6		
MQN7		
MQN8		X
MQN9		X
NumAliphaticCarbocycles		X
NumAliphaticHeterocycles		X
NumAliphaticRings		X
NumAmideBonds		
NumAromaticCarbocycles		
NumAromaticHeterocycles		
NumAromaticRings		

**Molecular Descriptors**	**Filtered at PCC = 0.95?**	**Filtered at PCC = 0.75?**
NumAtoms	X	X
NumHBA		X
NumHBD	X	X
NumHeavyAtoms	X	X
NumHeteroAtoms		X
NumLipinskiHBA	X	X
NumLipinskiHBD		
NumRings		
NumRotatableBonds		X
NumSaturatedCarbocycles		X
NumSaturatedHeterocycles		
NumSaturatedRings		X
NumStereocenters		
NumUnspecifiedStereocenters		
peoe_VSA1		X
peoe_VSA10		
peoe_VSA11		
peoe_VSA12		
peoe_VSA13		
peoe_VSA14		
peoe_VSA2		
peoe_VSA3		
peoe_VSA4		X
peoe_VSA5		
peoe_VSA6		
peoe_VSA7		
peoe_VSA8		
peoe_VSA9		X
SlogP		
slogp_VSA1		
slogp_VSA10		
slogp_VSA11		
slogp_VSA12		
slogp_VSA2		X
slogp_VSA3		X
slogp_VSA4		
slogp_VSA5		X
slogp_VSA6		X
slogp_VSA7		
slogp_VSA8		
slogp_VSA9		
SMR		
smr_VSA1		X
smr_VSA10		
smr_VSA2		
smr_VSA3		
smr_VSA4		X
smr_VSA5		X
smr_VSA6		X
smr_VSA7	X	X
smr_VSA8		
smr_VSA9		X
TPSA		
**118 descriptors**	**92 descriptors**	**56 descriptors**

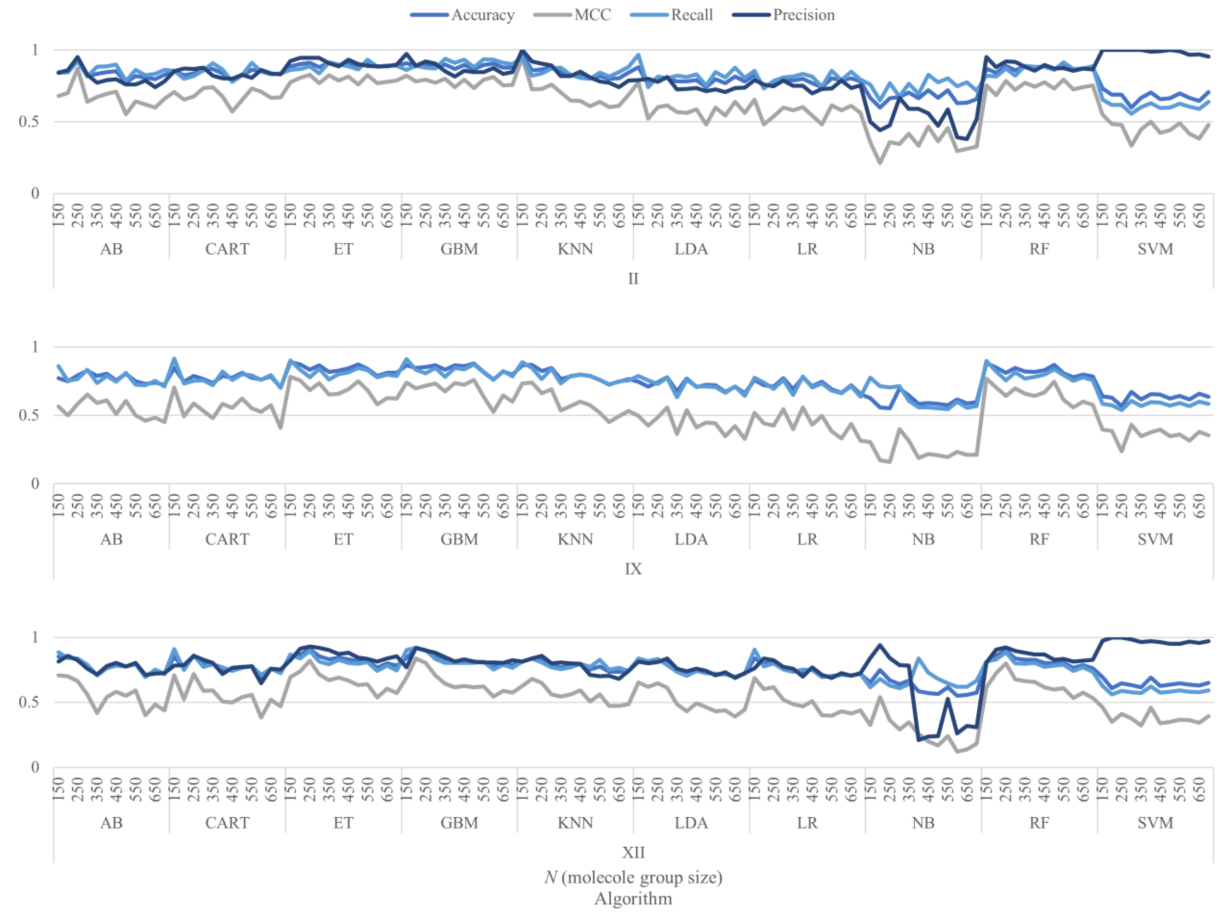

Fig. S1. Building models to predict activity classes: representation of variations of accuracy, Matthews Correlation Coefficient (MCC), precision and recall as group size (N), the algorithm used, and the investigated isoform vary for trained models in the testing phase. All models were built applying a Pearson Correlation Coefficient threshold of 0.95 to filter the most correlated features.

A)

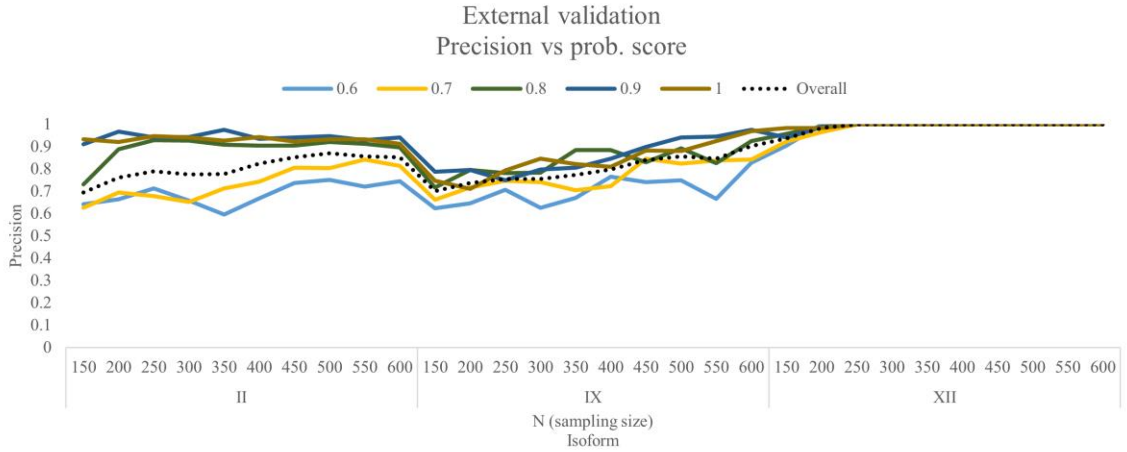

B)

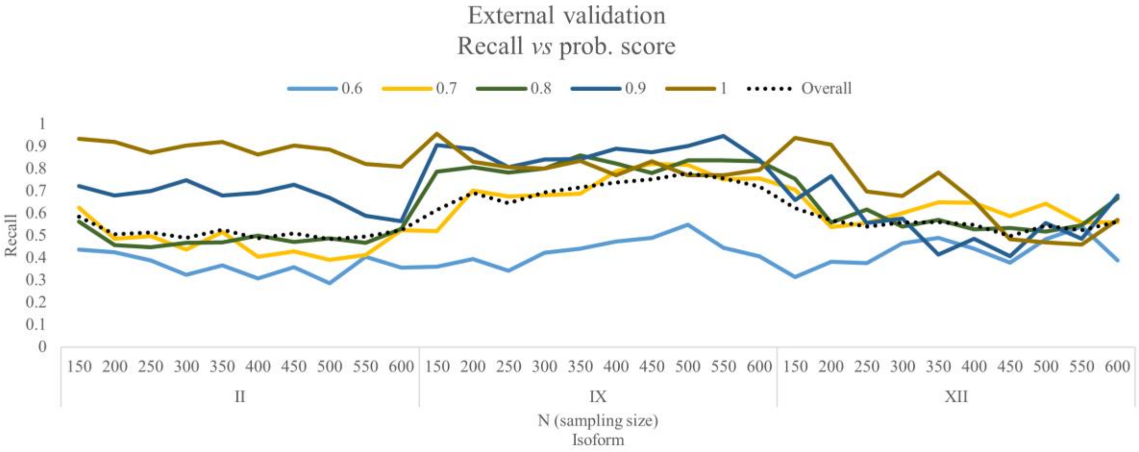

Fig. S2. External validation of the Extra Tree models: graphical representation of A) precision and B) recall at different values of the probability score. The overall precision and recall values for all probability label are reported as a black dotted line.
